# Maintenance of Species Differences in Closely Related Tetraploid Parasitic *Euphrasia* (Orobanchaceae) on an Isolated Island

**DOI:** 10.1016/j.xplc.2020.100105

**Published:** 2020-09-01

**Authors:** Hannes Becher, Max R. Brown, Gavin Powell, Chris Metherell, Nick J. Riddiford, Alex D. Twyford

**Affiliations:** 1 University of Edinburgh, School of Biological Sciences, Institute of Evolutionary Biology, Charlotte Auerbach Road, Edinburgh EH9 3FL, UK; 2 Royal Botanic Garden Edinburgh, 20A Inverleith Row, Edinburgh EH3 5LR, UK; 3 Botanical Society of Britain and Ireland, 4 High Firs Crescent, Harpenden, Hertfordshire AL5 1NA, UK; 4 Schoolton, Fair Isle, Shetland ZE2 9JU, UK

**Keywords:** incipient speciation, k-mer spectrum, allopolyploidy, tetraploid, divergence with gene flow, taxonomic complexity

## Abstract

Polyploidy is pervasive in angiosperm evolution and plays important roles in adaptation and speciation. However, polyploid groups are understudied due to complex sequence homology, challenging genome assembly, and taxonomic complexity. Here, we study adaptive divergence in taxonomically complex eyebrights (*Euphrasia*), where recent divergence, phenotypic plasticity, and hybridization blur species boundaries. We focus on three closely related tetraploid species with contrasting ecological preferences that are sympatric on Fair Isle, a small isolated island in the British Isles. Using a common garden experiment, we show a genetic component to the morphological differences present between these species. Using whole-genome sequencing and a novel k-mer approach we call “Tetmer”, we demonstrate that the species are of allopolyploid origin, with a sub-genome divergence of approximately 5%. Using ∼2 million SNPs, we show sub-genome homology across species, with a very low sequence divergence characteristic of recent speciation. This genetic variation is broadly structured by species, with clear divergence of Fair Isle heathland *Euphrasia micrantha*, while grassland *Euphrasia arctica* and coastal *Euphrasia foulaensis* are more closely related. Overall, we show that tetraploid *Euphrasia* is a system of allopolyploids of postglacial species divergence, where adaptation to novel environments may be conferred by old variants rearranged into new genetic lineages.

## Teaser

This study focuses on adaptive divergence in taxonomically complex eyebrights (*Euphrasia*), where recent origins, phenotypic plasticity, and hybridization blur species boundaries. Using a large common garden experiment, population genomics, and a novel k-mer app (Tetmer), this study reveals (1) a genetic component underpinning phenotypic differences, (2) complex patterns of genetic divergence between species, and (3) that the group shares an allopolyploid constitution.

## Introduction

Plant populations that grow in contrasting ecological conditions experience different selection pressures for adaptive traits that underlie survival and reproduction (Clausen et al., 1940, 1948; Clausen and Hiesey, 1958; Núñez-Farfán and Schlichting, 2001). This divergent ecological selection may cause adaptive divergence of populations and lead to the origin of novel ecotypes and species (McNeilly and Antonovics, 1968; Böhle et al., 1996; Baldwin and Sanderson, 1998; Nevado et al., 2016; Favre et al., 2017). The trajectory of divergence in the early stages of speciation is complex, with recent studies showing that populations may diverge in the face of ongoing gene flow that was previously thought sufficient to homogenize population differences and oppose divergence (Danley et al., 2000; Papadopulos et al., 2011; Nadeau et al., 2013; Richards et al., 2016). Although such insights have been made in different plant species, they are mostly ecological and evolutionary model systems amenable to genomic analysis (Bernasconi et al., 2009; Twyford et al., 2015). There are numerous plant groups that are underrepresented in current speciation genomic studies, and these include species that are characterized by recent polyploidy and groups with complex taxonomy where species boundaries are poorly understood.

Polyploidy, or whole-genome duplication, is common in angiosperms, with all extant species having experienced at least one round of polyploidy (Wendel et al., 2016). Whole-genome duplication is frequently linked to adaptation and speciation in plants (Wood et al., 2009; Levin, 2019). It may facilitate adaptation on its own in autopolyploids (Parisod et al., 2010; Baduel et al., 2019) or in conjunction with hybridization, creating allopolyploids. In allopolyploids, reduced recombination between homoeologous genes often facilitates the partitioning of functions (leading to subfunctionalization) and subsequent evolutionary changes (Cheng et al., 2018). Polyploidy increases genome size, which itself may affect fitness (Guignard et al., 2016), and it can increase adaptive potential, allowing organisms to colonize new environments and to tolerate stressful conditions (Lowe and Abbott, 1996; Ainouche et al., 2009; Soltis et al., 2012).

Although polyploidy is now widely appreciated as a key driver of plant diversification (Tank et al., 2015; Ren et al., 2018), there are considerable challenges in the study of polyploidy that limit our understanding of this key evolutionary process. Firstly, polyploidy is common in taxonomically complex groups and certain apomictic taxa, where species limits may be uncertain and where taxon identity is unknown (Popp et al., 2005; Guggisberg et al., 2006; Brysting et al., 2007). Secondly, comparative genomics of polyploids relies on correctly determining sequence homology, which is challenging in light of the additional gene copies from genome duplication (homoeologs). Thirdly, reference genome assembly, which is critical for many aspects of speciation genomics, such as genome scans for detecting outlier regions subject to selection (Ravinet et al., 2017; Campbell et al., 2018), is notoriously difficult in polyploids (although see Hollister et al., 2012). One way of circumventing the inference of sequence homology in a polyploid genome assembly is the use of methods based on DNA k-mers (DNA “words” of fixed length *k*). Such approaches have recently been used to infer ploidy and heterozygosity in polyploid samples (KAT, Mapleson et al., 2016; GenomeScope2 and Smudgeplots, Ranallo-Benavidez et al., 2020) and to identify genomic region(s) associated with a certain phenotype (“k-mers-based GWAS”, Voichek and Weigel, 2020). Such approaches could be extended to further characterize polyploid genome structure based on a demographic model of population divergence, without the need for a reference genome (Lohse et al., 2011, 2016).

The arctic and boreal regions of northern Europe are renowned for their diversity of polyploid taxa (Stebbins, 1984; Abbott and Brochmann, 2003; Brochmann et al., 2004), with eyebrights (*Euphrasia* L.), a genus of ∼263 species (A.D.T., unpublished data), as one of the most diverse. *Euphrasia* species are infamous for their complex morphological diversity, with many forms grading into others and being relatively indistinct. This taxonomic complexity in *Euphrasia* is a consequence of a diverse set of factors; the genus is characterized by recent rapid postglacial divergence (Gussarova et al., 2012; Wang et al., 2018), with species hybridizing extensively (Yeo, 1966; Stace, 2019). There is also variation in ploidy, and some species are highly selfing (Yeo, 1966; French et al., 2008; Stone, 2013). Moreover, *Euphrasia* are generalist facultative hemiparasites that are green and photosynthesize, but also attach to a host plant and steal nutrients and water. The growth of *Euphrasia* depends on the host species, with this phenotypic plasticity further contributing to taxonomic complexity (Brown et al., 2020). Although it is an incredibly complex genus, genomic studies of *Euphrasia* continue to reveal key insights into the nature of species differences and how hybridization, selfing, and phenotypic plasticity shape the evolution of polyploid taxa.

**Figure 1:**
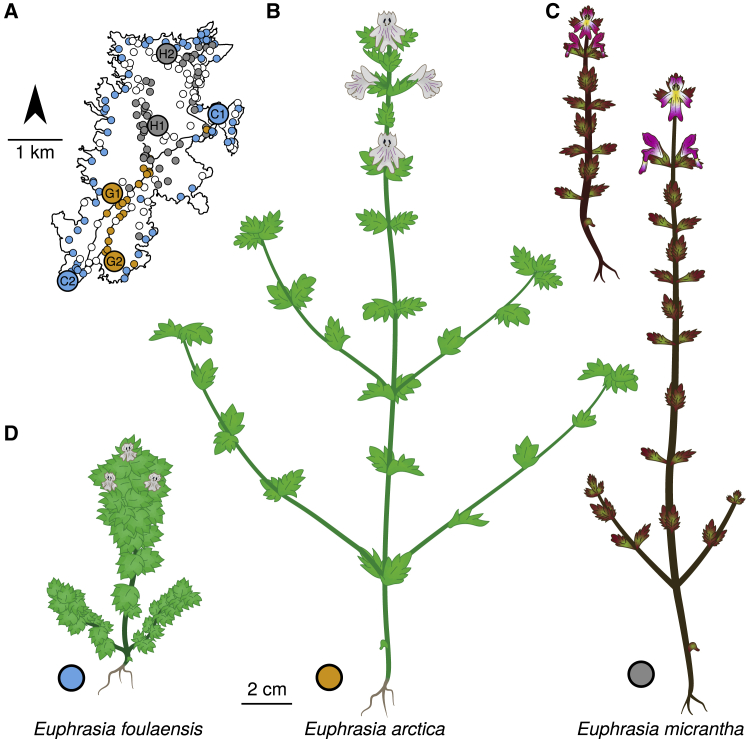
Three Focal *Euphrasia* Species and Their Distributions on Fair Isle. **(A)** Geographic distributions of *E. arctica*, *E. foulaensis*, and *E. micrantha* based on a field survey recording 282 presence (colored) or absence (white) census points. Labels indicate the populations from which seeds were sourced and traits were measured. **(B–D) (B)***E. arctica* and **(C)***E. micrantha*. The smaller form is typical for Fair Isle. **(D)***E. foulaensis*.

Given the scale of taxonomic complexity in *Euphrasia*, here we focus on co-occurring species on a single island. Fair Isle, a small island of 768 hectares, is the most remote inhabited island in the British Isles, situated halfway between Orkney (42 km away) and mainland Shetland (39 km away). Fair Isle *Euphrasia* provide an ideal study system due to their isolation from nearby gene flow and the closely intermixed habitats that support different species. The well-characterized island flora of 260 native species and 71 aliens (Quinteros Peñafiel et al., 2017) includes only tetraploid *Euphrasia:* eight *Euphrasia* species and eight putative hybrids (Riddiford et al., 2020). Here we focus on three tetraploid *Euphrasia* species that are widely distributed across the island but differ in their habitat preferences (see Figure 1). *Euphrasia arctica* is a grassland species that has vigorous growth and large flowers that are thought to be highly outcrossing. *Euphrasia foulaensis* is a coastal specialist with thickened leaves and compressed growth. It has small flowers, some of which do not open and are thought to be cleistogamous. *Euphrasia micrantha* is an upland heathland species that is slender. It often has pink-suffused leaves, stems, and flowers, and its very small flowers are highly selfing (Stone, 2013). *E. micrantha* is variable in morphology throughout its range, with the Fair Isle form smaller, usually unbranched, with magenta flowers with a less distinct lower floral “lip.” Despite the three species having different habitat preferences, the small scale of the island and fine-scale habitat differences mean species occur in sympatry (*sensu* Mallet et al., 2009) (Figure 1). To place these Fair Isle plants in a broader context, we also include diploid and tetraploid individuals from elsewhere in Great Britain.

In this study, we use field-based observations, a common garden experimental approach, and whole-genome sequencing to understand the nature of species differences in a group of taxonomically complex, sympatric, polyploid *Euphrasia* species. We address the following research questions: (1) is there a genetic component to the morphological differences between species with different habitat preferences? (2) what is the evolutionary history of polyploidization in *Euphrasia*? and (3) what is the landscape of genomic differentiation between sympatric species? To address question 2, we propose a novel k-mer-based analytical approach to infer sub-genome divergence in allopolyploids. Our results show how species differences are maintained over a fine spatial scale despite incomplete reproductive isolating barriers and reveal how genomic approaches can be used to characterize speciation histories of a non-model tetraploid group.

## Results

### Phenotypic Differences between *Euphrasia* Species Are Heritable

**Figure 2:**
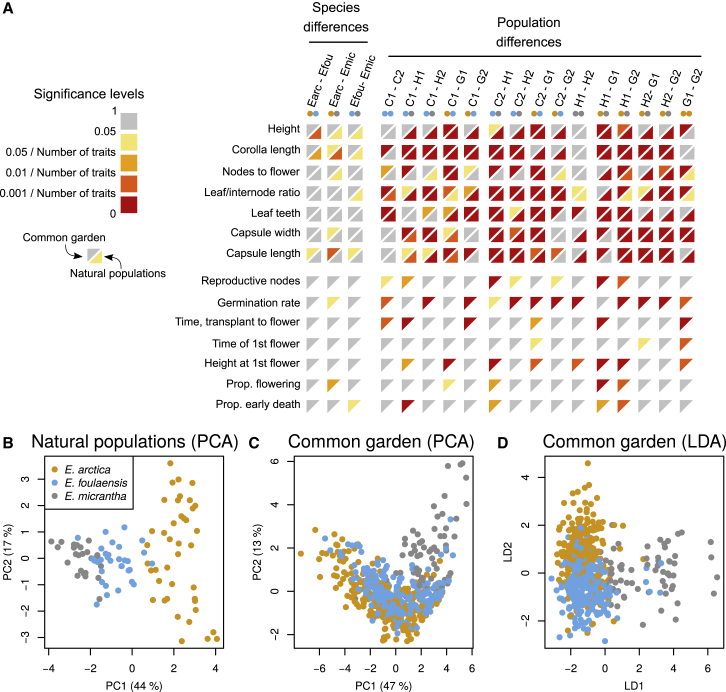
Morphological Trait Differentiation between Three Species of *Euphrasia* in a Common Garden Experiment and in Natural Populations. **(A)** Significance levels of trait differences between species (left) and populations (right) from field measurements in natural populations (bottom right triangles) and in the common garden (top left triangles, see Supplemental Text 1 for the magnitudes of differences and Supplemental Table 2 for the means and standard errors). Comparisons within rows are corrected for multiple testing. Within columns, the color scale is the *p* value corrected for the number of traits tested (seven in natural populations and 14 in the common garden). While significant trait differences are rare between species, they are numerous between populations (see Figure 1A or Supplemental Table 1 for population codes). **(B)** PCA of trait measurements from natural populations shows separate clusters per species. **(C)** PCA of trait measurements from plants grown in the common garden shows little grouping by species. **(D)** LDA separates species in the common garden.

To understand morphological differences among Fair Isle *Euphrasia* species, we compared trait variation in the wild (*n* = 180) and in a common garden experiment (*n* = 2116). We confirmed that in the wild *E. arctica* is tall (mean height 80.0 mm) and large flowered (mean corolla size 7.6 mm), *E. foulaensis* short (19.7 mm) and smaller flowered (5.9 mm), and *E. micrantha* intermediate in height (40.2 mm) and small-flowered (4.7 mm), with these trait values significantly different between all three species in mixed-effect models (*p* < 0.01; Figure 2A, Supplemental Tables 1 and 2, and Supplemental Text 1). Overall, however, many more traits showed significant differences at the population rather than the species level (Figure 2A, bottom right triangles). Based on multitrait phenotypes, individuals partly clustered by species in a principal component analysis (PCA, Figure 2B), while linear discriminant analysis (LDA) trained on 80% of the data classified all remaining individuals correctly. These results show that species and populations differ in their overall phenotype and in some key traits in natural populations.

To assess whether the phenotypic species differences have a genetic component, we analyzed morphological differentiation in a common garden setting by measuring individuals grown from seeds sourced from the same natural populations as above. Because host identity can affect performance in parasitic *Euphrasia* (Brown et al., 2020), we grew each individual with one of 12 different host species that occur on Fair Isle (Supplemental Table 2). Including host species as a random effect improved the fit of our models in terms of significantly lower Akaike Information Criterion values. Under these common garden conditions, there were fewer observable trait differences than in natural populations. The only trait showing significant differentiation between all species was capsule length, which was high in *E. arctica* (5.8 mm), intermediate in *E. foulaensis* (4.9 mm), and short in *E. micrantha* (3.7 mm) (*p* < 0.01). However, there were still some pairwise species differences, and many pairwise population differences (Figure 2A, top left triangles). Overall, the clustering of different species based on multitrait phenotypes in the common garden (Figure 2C) was much less obvious than in natural populations (Figure 2B). However, using LDA trained on 80% of the data (439 of 549 individuals without any missing data), it was possible to accurately classify 75% of the individuals in the test set (i.e., 82 of 110 individuals were assigned the species from which their seeds had been collected, Figure 2D). The success of LDA classification was high for *E. arctica* (78%) and *E. foulaensis* (75%), which misclassified individuals as the other species 22% of the time, while *E. micrantha* (62% classification success) was most commonly misclassified as *E. foulaensis* (25%). Overall, the presence of species-specific multitrait combinations in a common environment shows that there is a genetic component to the phenotypic species differences, but the lower classification success in a common garden indicates that many trait differences observed in the wild are due to plasticity.

### Complex Patterns of Plastid Genome and rDNA Relatedness

**Figure 3:**
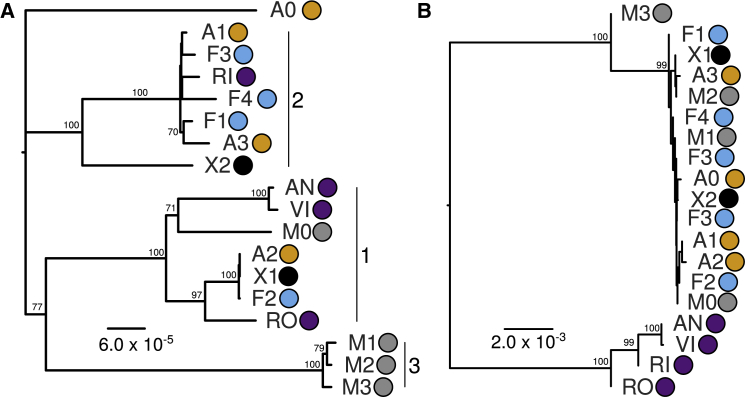
Evolutionary Relationships of British *Euphrasia* Plastid Genomes and rDNA Sequences. **(A)** Phylogenetic analysis of plastid genomes performed using a maximum-likelihood approach implemented in IQ-TREE with the K3Pu+F+I substitution model. Haplogroups are indicated by numbered vertical lines. **(B)** Phylogenetic analysis of nuclear rDNA sequences using a neighbor-joining approach implemented in Geneious. There were two rDNA haplotypes in sample F3. In both **A** and **B**, colored circles indicate species identity: orange, *E. arctica*; blue, *E. foulaensis*; gray, *E. micrantha*; black, putative hybrid individuals; and purple, diploid species. Scale bars indicate branch lengths. Numbers on branches indicate bootstrap support ≥70%.

We then generated whole-genome sequencing data for 18 *Euphrasia* individuals to investigate the genomics of species differences and polyploid history. Our samples included 12 Fair Isle tetraploids from our three focal species and two tetraploid individuals considered putative hybrids (labeled X1 and X2). The other samples were two tetraploids and four diploids from mainland Britain (see Supplemental Table 3 for details). *De novo* assembly of plastid genomes revealed complex patterns of plastid haplotype sharing and relatedness. Across samples, plastid genomes were similar in size (144 739–145 009 bp) and in sequence (>99.8% pairwise sequence identity). Fair Isle *Euphrasia* fell into three broad plastid haplogroups, each with 100% bootstrap support in our phylogenetic analysis (Figure 3A). Haplogroup 1 was composed of a mix of Fair Isle *Euphrasia* and mainland British diploid and tetraploid species. Haplogroup 2 was predominantly found in Fair Isle samples, plus the putative hybrid species *Euphrasia rivularis* sampled from England. Haplogroup 3 was exclusively composed of Fair Isle individuals of *E. micrantha*. Within each of these haplogroups, there was variable genetic divergence, with some extremely closely related haplotypes differing by a few SNPs, and some more divergent haplotypes showing structural genetic changes. Furthermore, there was plastid haplotype sharing between co-occurring samples of *E. arctica* and *E. foulaensis* on a roadside in the south of Fair Isle, as well as their putative hybrid (sample X1). Overall, these results indicate that diverse plastid haplotypes are maintained within an island population of *Euphrasia*, with Fair Isle *E. micrantha* differing from intermixed *E. arctica* and *E. foulaensis*.

*De novo* assembly and comparative analyses of *Euphrasia* rDNA revealed deep divergence between UK diploids and tetraploids, confirming previous results from internal transcribed spacer (ITS) sequencing of a broader taxonomic sample (Wang et al., 2018). Across the 5832-bp rDNA coding region, there was a mean pairwise diploid–tetraploid divergence of 2.1%, but the divergence in the ITS2 region was up to 10.8%. Within tetraploids, however, there was very limited sequence divergence, with >99.5% pairwise sequence identity between Fair Isle samples. rDNA haplotypes were shared between some individuals, such as *E. foulaensis* F2 and *E. arctica* A1, with one sample of *E. micrantha* maintaining a more divergent haplotype. These results support the recent divergence of species and populations, with extensive haplotype sharing particularly among *E. arctica* and *E. foulaensis*.

### Polyploid Genome Diversity Inferred with k-mer Analysis Methods

To assess the genomic properties of tetraploid *Euphrasia* without the need for genome assembly, we employed k-mer-based methods. In addition to using KAT, GenomeScope, and Smudgeplots, which visualize polyploid genome variations, we developed mathematical models for the shape of k-mer spectra of auto- and allotetraploids. We implemented these models in a “shiny” app, “Tetmer” (available from GitHub: https://github.com/hannesbecher/shiny-k-mers). Tetmer takes as an input a k-mer spectrum of a single tetraploid (or diploid) individual. In “Autofit” mode, it estimates the population genetic parameters θ (population-scaled mutation rate) and *T* (divergence time between homoeologous sub-genomes); it also fits the haploid (non-repetitive) genome size and a bias parameter (Vurture et al., 2017) accounting for the width of peaks in the k-mer spectrum. Tetmer can also be used in “Manual” mode to manually adjust the fit or to explore the expected shape of a k-mer spectrum under a certain model and parameter set.

Our approach is detailed in Supplemental Text 2. In brief, the characteristics of genetic variation in an autotetraploid is similar to those of four haploid samples from one panmictic population. The genetic variation within an allotetraploid, however, is more similar to that of two pairs of haploid samples from two diverged populations (between which gene flow ceased a certain time ago). This causes different distributions of genetic diversity that are evident in k-mer spectra. In autotetraploids, most variant sites contain singleton alleles (1/3 or 1/1/2), causing a prominent 1× peak. In allotetraploids, however, variant sites have mainly doubleton alleles (2/2), reflecting sub-genome divergence, and causing a prominent 2× peak.

**Figure 4:**
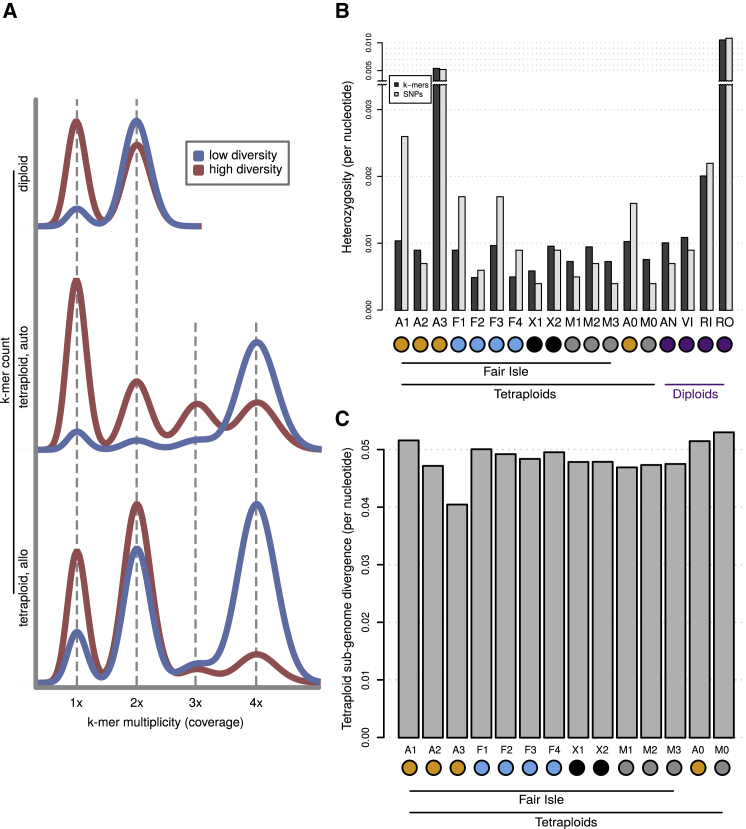
Estimates of Heterozygosity and Sub-genome Divergence in Allotetraploids Based on k-mer Spectra. **(A)** Schematic of the shapes of k-mer spectra of diploids, autotetraploids, and allotetraploids. Spectra of low-diversity species are shown in blue and high-diversity species in red. A general feature is that the higher the genetic diversity, the higher the 1× peak. Our app, Tetmer, allows these models to be fitted to empirical k-mer spectra. **(B)** Heterozygosity estimates for *Euphrasia* individuals based on k-mers (dark bars) and SNPs (light bars). **(C)** Estimates of sub-genome divergence for tetraploid *Euphrasia* individuals.

The k-mer spectra of all tetraploids analyzed showed prominent 2× peaks, clearly indicating allotetraploidy. Smudgeplots confirmed tetraploidy for all these samples (see “genome profiling” in Supplemental Text 3). We estimated the single-copy sequence in a haploid genome to be 185–225 Mb in our samples. This was generally about one-third higher than the “Genome Unique Length” reported by GenomeScope, which aims to exclude paralogous sequences. We then used Tetmer to estimate heterozygosity within and divergence between sub-genomes (Figure 4B and 4C). The heterozygosity estimates were noisy because they depend mainly on the 1× peak in the k-mer spectra (Figure 4A), which is often partly concealed by sequencing errors and contamination. We also estimated heterozygosity from SNPs called relative to a reference genome (discussed below) and by using GenomeScope on the diploids. Both approaches generated results similar to those from Tetmer. The average k-mer-based heterozygosity estimate over all samples was 0.2% and did not differ significantly between diploids and tetraploids (ANOVA, *p*_(*df*=16)_ = 0.07). Two samples, RO (*Euphrasia rostkoviana*) and A3 (*E. arctica*), had heterozygosity values considerably higher (1.1% and 0.52%, respectively), which was likely the result of recent outcrossing events in these mixed-mating species.

Finally, we used Tetmer to estimate the divergence between homoeologous sub-genomes of the tetraploids. Because the divergence estimate depends mainly on the relative size of the 2× and 4× peaks, it is more accurate than heterozygosity estimates based on the 1× peak. We found that all tetraploids showed a per-nucleotide sub-genome divergence of about 5%, one to two orders of magnitude higher than the heterozygosities observed (Figure 4C). This was similar to the heterozygosity class “aabb” report by GenomeScope, but Tetmer's estimate takes into account possible ancestral polymorphisms and heterozygosities within sub-genomes. The consistency of sub-genome divergence across samples raises the possibility of a common origin for these polyploids, involving the same or similar parental progenitors.

### Extensive Scaffold Homology between Diploids and Tetraploids

**Figure 5:**
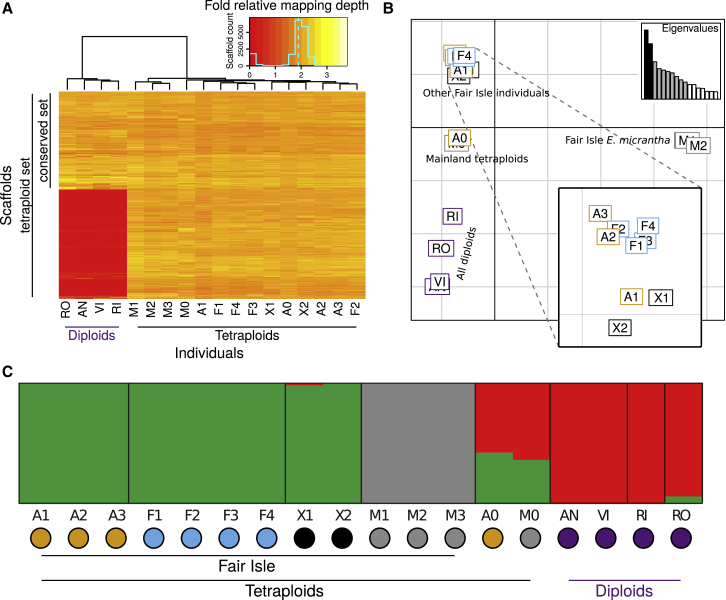
Diploid–Tetraploid Scaffold Homology and the Clustering of *Euphrasia* Populations. **(A)** Relative mapping depth in the tetraploid (2× depth in tetraploids) and conserved (2× depth all individuals) scaffold sets. Colors represent mapping coverage (see inset). Tetraploid scaffolds not contained in the conserved set have low mapping depths in diploids, indicative of absence (red). **(B)** PCA of genomic data (conserved scaffolds) separates Fair Isle *E. micrantha* individuals from other *Euphrasia*. PC2 separates tetraploids and diploids. The analysis was based on 3454 SNPs, with one SNP per scaffold. **(C)** STRUCTURE analysis shows Fair Isle *E. micrantha* as a separate genetic cluster. Analysis based on the same SNP data as in **(B)**. The plot shows the results for three genetic clusters (*K* = 3), with individuals on the x-axis and admixture proportions on the y-axis. The mainland tetraploids, M0 and A0, are inferred to be admixed. Colored outlines in **B** and dots in **C** represent taxa to match Figure 3.

To further our understanding of sub-genome divergence and polyploid history, we generated a draft genome assembly of a geographically isolated tetraploid sample of *E. arctica* from North Berwick, Scotland (individual A0). This hybrid assembly from 97× short-read Illumina and 16.7× long-read Pacific Biosciences (PacBio) data comprised 1 009 737 scaffolds, spanning 694 Mb in length. Although fragmented, the assembly was relatively complete, as indicated by the KAT completeness plot (Supplemental Figure 1) and the BUSCO completeness score of 81.7%. We then mapped genome resequencing data of all 18 diploids and tetraploid individuals to our reference assembly and classified scaffolds based on mapping depths. Only 0.1% of scaffolds (1024 scaffolds; 3 Mb) had 4× mapping depth in the tetraploids, as would be expected if they were autotetraploids, providing further support for the allopolyploid nature of the tetraploids. By contrast, 10 644 scaffolds (132 Mb) had a diploid-level (2×) mapping depth across all tetraploids, representing regions where diverged (homoeologous) sequences were assembled into separate scaffolds, which we called the “tetraploid” set. A subset of these tetraploid scaffolds had 2× mapping depth across all samples, representing regions homologous across ploidy levels (the remainder of the tetraploid scaffolds were mostly missing from diploids). We called these 3454 homologous scaffolds (46 Mb) the “conserved” set. The tetraploid and conserved sets formed the basis for subsequent population genomic analyses (Figure 5A). Overall, the consistency in mapping depth patterns between tetraploids suggests widespread sequence homology and possibly a common origin. Diploid–tetraploid homology supports the prediction that a (relative of a) British diploid was a progenitor of British tetraploids (Yeo, 1966), with a second sub-genome contributed by a divergent taxon.

### Strong Genetic Structure Despite Low Divergence

We used genetic clustering approaches to understand whether species from contrasting habitats are genetically cohesive. STRUCTURE analyses revealed that Fair Isle *E. micrantha* was a distinct genetic cluster in our sample of natural *Euphrasia* populations. Assuming three genetic clusters (*K* = 3, Figure 4C), there were clusters corresponding to Fair Isle *E. micrantha*, other Fair Isle taxa, and diploids. The mainland tetraploids (A0 and M0) were found to be admixed between clusters. At higher *K* values (Supplemental Figure 2), genetic clustering corresponded to broad taxonomic groupings, although *E. arctica* and *E. foulaensis* on Fair Isle were not separated. In PCA, Fair Isle *E. micrantha* was separated from all other species on principal component (PC) 1, while other Fair Isle species were separated from diploids on PC2, with mainland tetraploids in-between these groupings. Overall, these analyses point to divergence between Fair Isle *E. micrantha* and all other taxa being the major axis of divergence among our samples, rather than diploid–tetraploid divergence as found in other genetic analyses of *Euphrasia* (French et al., 2008; Wang et al., 2018).

**Figure 6:**
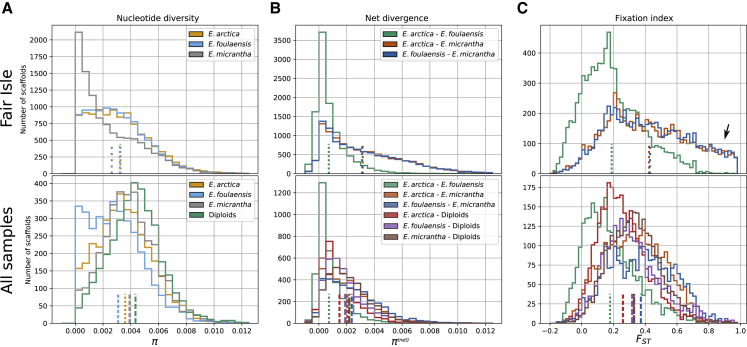
Considerable Genetic Structure with Little Differentiation between *Euphrasia* Species. Histograms show per-scaffold statistics with population means indicated by dashed lines at the bottom of each graph. The top row shows results based on the tetraploid data set for non-hybrid individuals from Fair Isle, while the bottom row is based on the conserved set, and it includes all non-hybrid individuals (with all diploids treated as one group). **(A)** Nucleotide diversity on Fair Isle is slightly lower in *E. micrantha* than in *E. arctica* and *E. foulaensis*, and overall, these values are considerably higher than per-individual heterozygosity estimates (Figure 4B), consistent with high levels of selfing. **(B)** On Fair Isle, the net divergence shows very similar patterns in the comparisons involving *E. micrantha*, while divergence between *E. arctica* and *E. foulaensis* is much lower. With wider sampling, the divergence between *E. micrantha* and the other species is lower, an indication that mainland *E. micrantha* carries alleles shared with the other species. The net divergences estimated here are of similar magnitude or lower than the nucleotide diversities shown in **(A)**. **(C)** Although net divergence tends to be low between species of *Euphrasia*, the fixation index can be extreme for some scaffolds, for example, Fair Isle comparisons including *E. micrantha* (arrow). This genetic differentiation disappears when samples from additional populations are included, indicating that allelic frequency divergence is greater at the population level than at the species level.

To better understand how variation is maintained within and between populations and species, we characterized genomic diversity and divergence based on 922 927 high-quality SNPs in the conserved and 2 166 914 SNPs in the tetraploid scaffolds. The per-species estimates of nucleotide diversity (*H*_*e*_) were considerably higher than individual heterozygosities, ranging from 0.26% to 0.32% on Fair Isle and from 0.31% to 0.43% when all non-hybrid individuals were considered. The total nucleotide diversity over all individuals (and species, *H*_*t*_) was 0.53%. A one order of magnitude difference between heterozygosity and population nucleotide diversity was observed even on Fair Isle (top of Figure 6A), where the aggregation of populations characterized by geographic isolation by distance is unlikely to play a role. Instead, these differences are likely to be the result of high levels of selfing. The per-nucleotide net divergence between species (*π*^(*net*)^ or *d*_*a*_) ranged from 0.07% (*E. arctica*–*E. foulaensis*) to 0.32% (*E. arctica*–*E. micrantha*) on Fair Isle, while this was lower (0.07%–0.24%) in the broader comparison (both comparisons excluded hybrid individuals). With the exception of comparisons including Fair Isle *E. micrantha*, these estimates of between-species divergence were lower than the diversity found within each species (Figure 6A and 6B).

Our finding of clear genetic clusters but low divergence suggests that species may be recent; however, these analyses do not indicate the amount of gene flow. We tested the extent of allele frequency differences, as measured by the fixation index (*F*_*ST*_), and found considerable genetic structure between Fair Isle *E. arctica* and *E. foulaensis*, on one hand, and *E. micrantha* on the other. In comparisons to *E. micrantha*, genetic structure was high, with mean *F*_*ST*_ values of 0.44 (*E. arctica*–*E. micrantha*) and 0.43 (*E. foulaensis*–*E. micrantha*), while 11% of scaffolds had *F*_*ST*_ > 0.8 and 38% of scaffolds had *F*_*ST*_ > 0.5. Both *F*_*ST*_ distributions showed very similar shapes (Figure 6C). Genetic structure was lower in the comparison between *E. arctica* and *E. foulaensis*, with a mean *F*_*ST*_ of 0.21, and only a minority of scaffolds with high values (0.11% > 0.8; 7.4% > 0.5). On a larger scale, including all non-hybrid individuals and treating the diploids as one group, the differentiation between species was lower and more similar across comparisons, with mean *F*_*ST*_ values ranging from 0.18 to 0.37, with the largest value for the comparison *E. foulaensis*–*E. micrantha* (Figure 6C, bottom). This is in line with previous *Euphrasia* studies that show genetic variation to be structured by region more than by species (Kolseth and Lönn, 2005; French et al., 2008).

### Genetic Relationships Cannot Be Explained by a Single Tree

**Figure 7:**
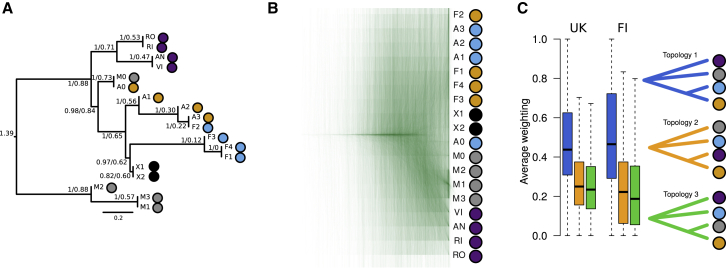
Complex Evolutionary Relationships and Extensive Discordance in *Euphrasia*. **(A)** ASTRAL consensus tree based on 3454 per-scaffold trees from the conserved scaffold set. The numbers are a node's posterior probability and its age in coalescent units. The tree is rooted at the longest branch (between Fair Isle *E. micrantha* and all other individuals). **(B)** Overlaid gene trees of the conserved scaffold set show no single clear species relationship. **(C)** Topological weighting of 3454 trees of all Fair Isle non-hybrids (FI) and all non-hybrid tetraploids (UK), both using the diploids as an outgroup, carried out with Twisst. While blue topology 1 tends to receive the highest weighting, few trees have a very high weight (near 1) for any one topology. The two alternative topologies receive similar levels of support. Colored dots represent taxa to match Figure 3.

To characterize genome-wide relationships of populations and species, we generated trees from thousands of genomic scaffolds and used these to produce a consensus tree indicative of the relationships of populations and species. The ASTRAL consensus tree had a high posterior probability (*PP* ≥ 0.97) for all nodes, except for the clade of mainland polyploids (*PP* = 0.82; Figure 7A). It did not generally show grouping by species; however, the diploids and Fair Isle *E. micrantha* were placed in distinct clades. The node ages, which are given in coalescent time, were comparatively young, with the oldest being 1.39 (in generations scaled by 2*N*_*e*_, corresponding to a coalescence probability of 75%). Such recent divergence gives many opportunities for incomplete lineage sorting. To better characterize the diverse genealogical histories present in the genome, we inspected the individual gene trees. Across 3454 scaffolds there was no clear congruence between individual trees and no general groupings by species, except some clustering of Fair Isle individuals of *E. micrantha* (M1–3; Figure 7B). This suggests that, while broadscale relationships reflect the divergence of Fair Isle *E. micrantha* from other tetraploids, and while diploids cluster separately, there is substantial gene tree incongruence and phylogenetic complexity.

To further categorize and quantify the different trees present in the genome, and to detect possible routes of gene flow, we conducted topology weighting with Twisst (Martin and van Belleghem, 2017). This approach compares individual trees to a set of reference topologies, and here we tested the three possible unrooted topologies of four groups (each tetraploid species, excluding hybrids, and all diploids as an outgroup). The most common topology placed *E. arctica* and *E. foulaensis* as closest relatives and *E. micrantha* on a separate branch, while both alternative topologies had lower weights (Figure 7C). However, few of the 3454 per-scaffold trees matched one of the three reference topologies (419, 15, and 10, respectively). The topology mean weights were similar when mainland tetraploids were included (47.2%, 25.4%, and 27.5%, with 105, 3, and 1 tree matching the reference topologies entirely). For both Fair Isle and the wider sampling, the topology with *E. arctica* and the diploids as closest relatives had a somewhat higher weight than the alternative (*E. micrantha* and *E. arctica* as closest relatives), suggesting possible gene flow between *E. arctica* and the diploids, or between *E. micrantha* and *E. foulaensis*. We tested this with Patterson's *D* statistic using the diploids as an outgroup and found that *D* deviated significantly from zero (*D* = 0.02, *p* = 0.005) only when the mainland tetraploids were included. This suggests gene flow between (mainland) *E. arctica* and the diploids, with an admixture fraction measured as *F*_*G*_ of 2.5% (Green et al., 2010). This value should be interpreted as additional admixture on top of any background level between all pairs of species.

## Discussion

Adaptive divergence is a major driver of speciation but remains poorly studied in polyploid organisms. In the present study, we use whole-genome sequencing in combination with common garden experimental approaches to understand the nature of species differences in a complex tetraploid group. We focus on an isolated island system where we can characterize genome-wide diversity and divergence of three differentially adapted eyebright species in sympatry. In our focal species, we find that phenotypic differences are underpinned by genetic differences, providing support for the recognition of species differences in this taxonomically complex group. Building upon this finding, in the discussion that follows, we first consider the technical aspects of polyploid data analysis and the new insights we have gained into polyploid evolution, before discussing the landscape of genomic differentiation and adaptation in *Euphrasia*.

### Polyploid Genome Dynamics

Polyploid-aware analyses are key to understanding the evolutionary histories of British eyebrights and many other postglacial groups such as octo- and dodecaploid *Cerastium* (Brysting et al., 2007)*,* tetra-hexaploid species of *Silene* (Popp et al., 2005), the highly variable ploidy *Primula* section *Aleuritia* (Guggisberg et al., 2006), and *Dactylorhiza* orchids (Brandrud et al., 2019). While such analyses used to involve the cloning of homoeologous sequences and comparisons of tree topologies, the use of whole-genome data without assembly is becoming increasingly popular. Several k-mer-based methods are now used routinely in genome-sequencing projects of any complexity. For instance, KAT (Mapleson et al., 2016) and GenomeScope (Vurture et al., 2017) can be used to estimate the heterozygosity of diploids and to characterize polyploid k-mer spectra, while Smudgeplots (Ranallo-Benavidez et al., 2020) can be used to determine the ploidy of a sample. However, these programs do not explicitly model the mechanism underpinning genetic diversity in allopolyploid genomes. Tetmer partly fills this gap by explicitly assuming (and allowing researchers to estimate) divergence between allotetraploid sub-genomes, with this corresponding to divergence over time between isolated populations. Tetmer can also be used to analyze autotetraploid and diploid genomes and to visualize the expected k-mer spectrum given a set of parameters. Similar to other k-mer methods, it works best on short-read data of suitable coverage (haploid peak >15×) generated from a single individual, ideally using contaminant-free samples and PCR-free sequencing libraries.

Using Tetmer on k-mer spectra of *Euphrasia* revealed considerable between-individual variation in heterozygosity within species, indicative of a mixed-mating system. This approach of analyzing divergence within an individual proves complementary to comparative genomics between diploids and tetraploids, which revealed a set of scaffolds shared between diploids and tetraploids. This points to a (relative of a) British diploid acting as a parental progenitor to the British tetraploids, with the origin of the other, highly divergent, sub-genome (∼5% divergence) unknown.

The likely absence in Britain of the second diploid ancestor—the Wang et al. (2018) barcoding study failed to find the tetraploid rDNA haplotype in British diploids—and distinct genetic clustering of the diploids suggest that allopolyploidy in *Euphrasia* is not recent. This proves problematic for dating the polyploidy event(s), which would require extant but genetically isolated diploid relatives (Doyle and Egan, 2010). A further complication is that ongoing gene flow, evidenced by plastid sharing and the presence of natural diploid–tetraploid hybrids (Yeo, 1956), blurs the split between diploids and tetraploids. Polyploid species tend to go through a process of diploidization, involving the reduction in chromosome number, homoeologous rearrangements, and gene losses, leaving paleopolyploids with prepolyploidy genome sizes (Mandáková and Lysak, 2018). This process does not seem to progress at equal pace in different polyploids. For instance, *Brassica napus*, a recent allopolyploid formed 7500–12 500 years ago with extant diploid relatives, experienced extensive homoeologous rearrangements (Chalhoub et al., 2014). By contrast, teff (*Eragrostis tef*, VanBuren et al., 2020) formed 1.1 million years ago without known diploid ancestors, and *Capsella bursa-pastoris* formed about 100 000 years ago (Douglas et al., 2015; Kryvokhyzha et al., 2019) do not show large-scale genomic rearrangements. Thus, homoeologous exchange between sub-genomes and rearrangements has major effects on interspecies gene flow, divergence, and ultimately adaptation and speciation. These questions of sub-genome structure and genomic rearrangements will be addressed with an improved genome assembly, generated by the Darwin Tree of Life project (https://www.darwintreeoflife.org), and further population sequencing.

### Landscape of Genomic Differentiation and Speciation History

Studies of speciation genomics may gain valuable insights by investigating taxa at different stages of the speciation trajectory, from the earliest stages of divergence to genetically differentiated species with strong reproductive barriers (Via, 2009; Twyford et al., 2014). We have shown that three species of eyebright adapted to contrasting environments are characterizsed by a gradient of genomic differentiation, with grassland *E. arctica* and coastal *E. foulaensis* closely related, while the dry heathland specialist *E. micrantha* is genetically and morphologically more distinct. This parallels other genomic studies of adaptive divergence where species boundaries show different degrees of permeability, underpinned by different reproductive isolating barriers (Peccoud et al., 2009; Dasmahapatra et al., 2012; Nosil et al., 2012; Renaut et al., 2012; Roda et al., 2013). Reproductive isolation between *E. arctica* and *E. foulaensis* is incomplete and there is evidence of ongoing gene flow. However, the long tail in the pairwise *F*_*ST*_ distribution reveals many differentiated scaffolds, which may represent important regions of divergence involved in adaptation to harsh coastal environments (Lowry et al., 2008; Lyu et al., 2018; Dong et al., 2020) and the maintenance of species identities (Nosil et al., 2009; Ravinet et al., 2017). By contrast, *E. micrantha* shows genome-wide divergence from the other two taxa, despite a similar degree of geographic co-occurrence. The high selfing rate of *E. micrantha* (Stone, 2013) and intrinsic postzygotic barriers (genomic incompatibilities) are likely to underlie reproductive isolation. However, our genomic analyses suggest that the genetic distinctness of *E. micrantha* may be a feature of the Fair Isle population rather than the species as a whole. This could be a consequence of regional differentiation caused by strong drift and the local fixation of genomic incompatibilities, local hybridization, or polytopic origins as seen in other polyploids (Schwarzbach and Rieseberg, 2002; Soltis et al., 2004; Ainouche et al., 2012; Lowe and Abbott, 2015).

Our data provide insights into the enigmatic relationships of eyebrights, showing that their speciation history has been shaped by allopolyploidy with consistent sub-genome divergence, that overall species divergence is extremely shallow, and no single phylogenetic tree can represent their evolutionary history. Genome-scale data for adaptive radiations have revealed many groups characterized by reticulate evolution, and further characterization of this reticulation is a main focus of speciation biology (Rieseberg et al., 2003; Cui et al., 2013; Malinsky et al., 2018; Sun et al., 2018; Feng et al., 2019). Hybridization, followed by adaptive introgression, is advantageous as it substantially reduce the waiting times for new variants to evolve. Similar to many other rapid radiations, the relationships of tetraploid eyebrights are reticulate, and reproductive isolation between them is incomplete, comparable to tetraploid species of *Arabidopsis* (Jørgensen et al., 2011). In such settings, it is easy for adaptive variants to cross species boundaries (Morjan and Rieseberg, 2004), generating novel phenotypic and genic combinations, which may lead to differential adaptation and reproductive isolation (Pardo-Diaz et al., 2012). Partial selfing may exacerbate this, as is seen in *Epipactis* orchids (Squirrell et al., 2002). This diversification process, termed “combinatorial speciation” (Marques et al., 2019), is likely to underlie adaptation and the formation of taxonomic complexity in the young, and thus mutation-limited, group of tetraploid *Euphrasia*.

## Methods

### Phenotypic Differentiation

#### Natural Populations

To establish the extent of phenotypic species differences, we measured morphological traits for *E. arctica*, *E*. *foulaensis*, and *E*. *micrantha* in natural populations on Fair Isle. For each of two populations per species (Supplemental Table 1), we measured 30 individual plants in the field at a single census point. The set of seven traits that we measured is commonly used in *Euphrasia* identification (Metherell and Rumsey, 2018) and includes plant height, corolla length, number of nodes below the first flower, length ratio of the internode below the first flower and the leaf subtending the first flower, number of leaf teeth, capsule width, and capsule length. All length measurements were made in millimeters, to the nearest 0.1 mm, using digital calipers.

#### Common Garden Experiment

We obtained seeds from wild-collected and open-pollinated plants, and pooled seeds within a population (Supplemental Table 1). We then placed seeds in individual pots filled with a peat-free soil mix, which is a bark-based substrate of neutral pH (RGBE1), in December. We set up 4320 pots, corresponding to three species of *Euphrasia* with two replicate populations, 12 different host species (see below), and 60 replicates for each combination. These pots were kept outside at the Royal Botanic Garden Edinburgh (RGBE). We recorded germination daily from the beginning of April and supplied *Euphrasia* with host plants (with seeds sourced from Fair Isle and commercial seed suppliers, see Supplemental Table 3) at intervals of about 2 weeks. At the time of first flowering, we measured the same traits as in natural populations. At the end of September (or at the time of death), for each plant, we recorded its final height, number of reproductive nodes (a measure of fitness), and length and width of one capsule. We also recorded which plants had died before the end of the experiment.

#### Morphological Trait Analysis and Data Visualization

Trait differences were assessed with linear mixed-effect models (R version 3.6.1 package lme4, https://github.com/lme4/lme4/). In the common garden study, host species was used as a random effect, and transplant time was included as a covariate if the model's log likelihood improved significantly. When analyzing trait differences between species, genotype was included as a (nested) random effect. The significance of trait differences between populations or species was assessed with a general linear hypothesis test (function “glht”) as implemented in the R package “multcomp” (version 1.4-10). The *p* values of trait differences were corrected for multiple tests at the level of each trait (rows in Figure 2A).

To visualize the individual clustering by phenotype, we carried out PCA with R's built-in function “princomp.” To assess how well individuals could be classified into species, we ran LDAs, function “lda” of the R package “MASS,” version 7.3-51.4). For plotting, we ran “lda” without cross-validation and used the function “predict” to obtain two scores for each individual, which were plotted in two dimensions.

### Genomic Sequencing and Analyses

#### Sample Processing and Sequencing

We collected individual *Euphrasia* plants in the field into desiccating silica gel. After grinding samples with a tissue mill using ceramic beads, we extracted DNA using the Qiagen DNeasy Plant Mini Kit following the manufacturer's instructions. For the *E. arctica* reference sample, DNA extraction was performed by Amplicon Express using their in-house high-molecular-weight DNA extraction protocol.

We generated short-read Illumina sequence data and long-read PacBio data for a range of *Euphrasia* samples. Full details of the sequencing protocols and specifications are provided in Supplemental Table 4.

#### Plastid Genome and rDNA Analyses

Plastid genomes were assembled from paired Illumina reads of each *Euphrasia* sample using NOVOplasty. Assembled plastids were manually curated and edited to give a standard order of the large single copy, inverted repeat 1, small single copy, and inverted repeat 2 using Geneious v.11.1. Genome annotation of *E. arctica* A1 was performed using DOGMA (Wyman et al., 2004) with manual editing and curation, and annotation was carried over to other samples using the “annotate from database” option in Geneious. Plastid genomes were aligned using MAFFT. Phylogenetic analyses were performed using IQ-TREE (Nguyen et al., 2014), with the best evolutionary model inferred using model fitting and model assessment based on Bayesian information criterion, and the level of branch support inferred via 1000 rapid bootstrap replicates. To help infer the direction of evolutionary change, we included an unpublished plastid assembly of the divergent *E. antarctica* (A.D.T. and R. Ness, unpublished data) in the initial alignment and tree building. We used this sample to root the phylogeny, and subsequently removed the long branch for better tree visualization. The final alignment of British *Euphrasia* samples included 144 899 constant sites, 174 parsimony-informative sites, and 133 singleton sites.

rDNA was assembled from the same read data as described above, except that we set the expected assembly size to 9000–20 000 bp and used a 1380-bp seed sequence of the rDNA cluster that was obtained from a run of the RepeatExplorer pipeline (Novák et al., 2013). The assembler produced variable results, with some species having fully assembled circularized arrays, while some had multiple contigs. Initial comparisons of assembled arrays suggested that some taxa were not alignable outside the rDNA coding region (results not shown). We therefore trimmed assemblies to the 5.8-kb rDNA coding region (comprising 18S, ITS1, 5.8S, ITS2, 26S). Pairwise alignments were performed with MAFFT, and a neighbor-joining tree was constructed using Geneious.

#### k-mer Analyses

We modeled the shape of k-mer spectra based on the infinite alleles model. Diploids and autotetraploids were treated as samples of two or four genomes from a single population. Allotetraploids were treated as two samples from each of the two diverged populations. The corresponding formulae were derived using the block-wise site-frequency spectrum framework (Lohse et al., 2011, 2016). Supplemental Text 2 describes this in more detail.

All models are implemented in our app, Tetmer, which can be used to fit parameters to k-mer spectra. This app is available from GitHub (https://github.com/hannesbecher/shiny-k-mers).

#### Mapping and Variant Analysis

We generated a reference assembly of *E. arctica* from Illumina paired-end and PacBio data using the hybrid assembler SPAdes (Bankevich et al., 2012). The assembly was polished with ntEdit (Warren et al., 2019). The k-mer completeness of the assembly, compared with the sequencing data, was assessed with the k-mer analysis toolkit (Mapleson et al., 2016). Inspection with blobtools (Laetsch and Blaxter, 2017) revealed that there was no contamination at the indicated sequencing depths, and the CG content was similar to that of *Euphrasia* DNA. We removed all scaffolds with an average mapping depth <40× (inspection with blobtools had shown that this threshold separated contaminations from target scaffolds).

We mapped short-read data generated from natural populations to the reference using BWA-MEM (https://arxiv.org/abs/1303.3997v2), and we computed per-scaffold mapping depths. These computations were automated using Snakemake pipelines (Köster and Rahmann, 2012). We then compared the mapping depth information across individuals. Two sets of scaffolds were selected for variant calling: the conserved dataset, which comprised 3454 scaffolds with disomic coverage in all individuals, and the tetraploid set, which comprised the conserved set and 7189 more scaffolds restricted to tetraploid individuals (totaling 10 643 scaffolds). All other scaffolds of the reference were concatenated and maintained in the assembly to avoid non-specific mapping of reads from diverged regions to our scaffold set. We called variants with freebayes (https://arxiv.org/abs/1207.3907) separately for the conserved and tetraploid scaffold sets. In the resulting VCF files, we used only biallelic SNPs with a quality value greater than 100. The VCF files were handled interactively in jupyter lab (https://github.com/jupyterlab) using the scikit-allel package (https://doi.org/10.5281/zenodo.3238280). An HTML report of the analyses is included in the Zenodo archive published alongside this article (https://doi.org/10.5281/zenodo.3774489).

#### Analysis of Population Structure

We used two complementary approaches to infer population structure. To avoid spurious signals from sites in linkage disequilibrium in these analyses, we selected one random polymorphism per scaffold from the “conserved” scaffold set. We then carried out PCA as implemented in the adegenet package version 2.1.2 (Jombart and Ahmed, 2011) and ran FastStructure (Raj et al., 2014), with *K* values ranging from 1 to 10. As FastStructure may underestimate the extent of admixture, we then analyzed the same dataset in STRUCTURE (Pritchard et al., 2000) using a subset of *K*values (*K* = 1–5) that were deemed to be most likely. STRUCTURE was run with the admixture model using three replicates per *K* value, with 100 000 burn-in generations followed by 100 000 MCMC generations. The optimal number of clusters was inferred using the ad hoc statistic delta *K* (Evanno et al., 2005). We combined multiple STRUCTURE runs in CLUMPP (Jakobsson and Rosenberg, 2007) and visualized STRUCTURE plots using a custom approach.

#### Per-Scaffold Trees

We generated per-scaffold unrooted neighbor-joining trees based on pairwise estimates of net-nucleotide divergence between individuals using the nj function of the R package ape v.5.3 (Paradis and Schliep, 2018). These trees were used for a number of downstream analyses, as follows. We generated a consensus tree using ASTRAL (Zhang et al., 2018). We rooted the tree at the longest branch, which connected Fair Isle *E. micrantha* to all the other samples. We then performed topology weighting as implemented in the package Twisst (Martin and van Belleghem, 2017). As candidate topologies, we used all three possible trees with four groups (diploid outgroup and one group per species of *Euphrasia*). We ran Twisst twice, once on the Fair Isle samples, and once on the mainland tetraploids. Finally, we carried out two sets of ABBA-BABA tests with Dsuite (Malinsky et al., https://doi.org/10.1101/634477) using all diploids as the outgroup. Firstly, we used all non-hybrid individuals from Fair Isle, grouped by species, and secondly, we added the mainland tetraploids according to their species.

## Funding

This work was funded by 10.13039/501100000270NERC grants (NE/R010609/1; NE/L011336/1; NE/N006739/1) awarded to A.D.T.

## Author Contributions

H.B. and A.D.T. designed the experiments. H.B., A.D.T., G.P., and M.R.B. set up the experiments. All authors collected the data. C.M. confirmed the species identifications. N.J.R. provided valuable support on Fair Isle. H.B. analyzed the data. H.B. and A.D.T. wrote the manuscript. All authors commented on and approved the manuscript.

## Accession Numbers

Sequence data from this article can be found in the Sequence Read Archive (SRA) under the accession numbers listed in Supplemental Table 4. Additional scripts and data can be found in the Zenodo repository with the https://doi.org/10.5281/zenodo.3774489. The genome assembly for *E. arctica* can be found under GenBank accession no. JACOMQ000000000.

## Supplemental Information

Document S1. Supplemental Figures 1 and 2, Supplemental Tables 1 and 3, Supplemental Text 1–3, and Supplemental References

Supplemental Table 2. Means and Standard Errors of Trait Measurements from Natural Populations (Fair Isle) and from the Common Garden

Supplemental Table 4. Individuals Sequenced, Heterozygosity, and Sub-genome Divergence Estimates

Document S2. Article plus Supplemental Information
