# Supplementary material for: Maintenance of Species Differences in Closely Related Tetraploid Parasitic Euphrasia (Orobanchaceae) on an Isolated Island
Source: Plant Commun. 2020 Sep 1;1(6):100105. doi: 10.1016/j.xplc.2020.100105 (PMC7748025; doi:10.1016/j.xplc.2020.100105)
Supplement: Document S2. Article plus Supplemental Information [file mmc4.pdf]

Plant Communications
Research Article

CellPress
Partner Journal

Maintenance of Species Differences in Closely Related Tetraploid Parasitic *Euphrasia* (Orobanchaceae) on an Isolated Island

Hannes Becher[1,*], Max R. Brown[1], Gavin Powell[2], Chris Metherell[3], Nick J. Riddiford[4] and Alex D. Twyford[1,2,*]

[1]University of Edinburgh, School of Biological Sciences, Institute of Evolutionary Biology, Charlotte Auerbach Road, Edinburgh EH9 3FL, UK

[2]Royal Botanic Garden Edinburgh, 20A Inverleith Row, Edinburgh EH3 5LR, UK

[3]Botanical Society of Britain and Ireland, 4 High Firs Crescent, Harpenden, Hertfordshire AL5 1NA, UK

[4]Schoolton, Fair Isle, Shetland ZE2 9JU, UK

***Correspondence: Hannes Becher (hbecher@ed.ac.uk), Alex D. Twyford (alex.Twyford@ed.ac.uk)**

https://doi.org/10.1016/j.xplc.2020.100105

ABSTRACT

Polyploidy is pervasive in angiosperm evolution and plays important roles in adaptation and speciation. However, polyploid groups are understudied due to complex sequence homology, challenging genome assembly, and taxonomic complexity. Here, we study adaptive divergence in taxonomically complex eyebrights (*Euphrasia*), where recent divergence, phenotypic plasticity, and hybridization blur species boundaries. We focus on three closely related tetraploid species with contrasting ecological preferences that are sympatric on Fair Isle, a small isolated island in the British Isles. Using a common garden experiment, we show a genetic component to the morphological differences present between these species. Using whole-genome sequencing and a novel k-mer approach we call "Tetmer", we demonstrate that the species are of allopolyploid origin, with a sub-genome divergence of approximately 5%. Using ∼2 million SNPs, we show sub-genome homology across species, with a very low sequence divergence characteristic of recent speciation. This genetic variation is broadly structured by species, with clear divergence of Fair Isle heathland *Euphrasia micrantha*, while grassland *Euphrasia arctica* and coastal *Euphrasia foulaensis* are more closely related. Overall, we show that tetraploid *Euphrasia* is a system of allopolyploids of postglacial species divergence, where adaptation to novel environments may be conferred by old variants rearranged into new genetic lineages.

Key words: incipient speciation, k-mer spectrum, allopolyploidy, tetraploid, divergence with gene flow, taxonomic complexity

Becher H., Brown M.R., Powell G., Metherell C., Riddiford N.J., and Twyford A.D. (2020). Maintenance of Species Differences in Closely Related Tetraploid Parasitic *Euphrasia* (Orobanchaceae) on an Isolated Island. Plant Comm. **1**, 100105.

INTRODUCTION

Plant populations that grow in contrasting ecological conditions experience different selection pressures for adaptive traits that underlie survival and reproduction (Clausen et al., 1940, 1948; Clausen and Hiesey, 1958; Núñez-Farfán and Schlichting, 2001). This divergent ecological selection may cause adaptive divergence of populations and lead to the origin of novel ecotypes and species (McNeilly and Antonovics, 1968; Böhle et al., 1996; Baldwin and Sanderson, 1998; Nevado et al., 2016; Favre et al., 2017). The trajectory of divergence in the early stages of speciation is complex, with recent studies showing that populations may diverge in the face of ongoing gene flow that was previously thought sufficient to homogenize population differences and oppose divergence (Danley et al., 2000; Papadopulos et al., 2011; Nadeau et al., 2013; Richards et al., 2016). Although such insights have been made in different plant species, they are mostly ecological and evolutionary model systems amenable to genomic analysis (Bernasconi et al., 2009; Twyford et al., 2015). There are numerous plant groups that are underrepresented in current

Published by the Plant Communications Shanghai Editorial Office in association with Cell Press, an imprint of Elsevier Inc., on behalf of CSPB and IPPE, CAS.

This is an open access article under the CC BY license (http://creativecommons.org/licenses/by/4.0/).

speciation genomic studies, and these include species that are characterized by recent polyploidy and groups with complex taxonomy where species boundaries are poorly understood.

Polyploidy, or whole-genome duplication, is common in angiosperms, with all extant species having experienced at least one round of polyploidy (Wendel et al., 2016). Whole-genome duplication is frequently linked to adaptation and speciation in plants (Wood et al., 2009; Levin, 2019). It may facilitate adaptation on its own in autopolyploids (Parisod et al., 2010; Baduel et al., 2019) or in conjunction with hybridization, creating allopolyploids. In allopolyploids, reduced recombination between homoeologous genes often facilitates the partitioning of functions (leading to subfunctionalization) and subsequent evolutionary changes (Cheng et al., 2018). Polyploidy increases genome size, which itself may affect fitness (Guignard et al., 2016), and it can increase adaptive potential, allowing organisms to colonize new environments and to tolerate stressful conditions (Lowe and Abbott, 1996; Ainouche et al., 2009; Soltis et al., 2012).

Although polyploidy is now widely appreciated as a key driver of plant diversification (Tank et al., 2015; Ren et al., 2018), there are considerable challenges in the study of polyploidy that limit our understanding of this key evolutionary process. Firstly, polyploidy is common in taxonomically complex groups and certain apomictic taxa, where species limits may be uncertain and where taxon identity is unknown (Popp et al., 2005; Guggisberg et al., 2006; Brysting et al., 2007). Secondly, comparative genomics of polyploids relies on correctly determining sequence homology, which is challenging in light of the additional gene copies from genome duplication (homoeologs). Thirdly, reference genome assembly, which is critical for many aspects of speciation genomics, such as genome scans for detecting outlier regions subject to selection (Ravinet et al., 2017; Campbell et al., 2018), is notoriously difficult in polyploids (although see Hollister et al., 2012). One way of circumventing the inference of sequence homology in a polyploid genome assembly is the use of methods based on DNA k-mers (DNA "words" of fixed length *k*). Such approaches have recently been used to infer ploidy and heterozygosity in polyploid samples (KAT, Mapleson et al., 2016; GenomeScope2 and Smudgeplots, Ranallo-Benavidez et al., 2020) and to identify genomic region(s) associated with a certain phenotype ("k-mers-based GWAS", Voichek and Weigel, 2020). Such approaches could be extended to further characterize polyploid genome structure based on a demographic model of population divergence, without the need for a reference genome (Lohse et al., 2011, 2016).

The arctic and boreal regions of northern Europe are renowned for their diversity of polyploid taxa (Stebbins, 1984; Abbott and Brochmann, 2003; Brochmann et al., 2004), with eyebrights (*Euphrasia* L.), a genus of ~263 species (A.D.T., unpublished data), as one of the most diverse. *Euphrasia* species are infamous for their complex morphological diversity, with many forms grading into others and being relatively indistinct. This taxonomic complexity in *Euphrasia* is a consequence of a diverse set of factors; the genus is characterized by recent rapid postglacial divergence (Gussarova et al., 2012; Wang et al., 2018), with species hybridizing extensively (Yeo, 1966; Stace, 2019). There is also variation in ploidy, and some species are highly selfing (Yeo, 1966; French et al., 2008; Stone, 2013). Moreover, *Euphrasia* are generalist facultative hemiparasites that are green and photosynthesize, but also attach to a host plant and steal nutrients and water. The growth of *Euphrasia* depends on the host species, with this phenotypic plasticity further contributing to taxonomic complexity (Brown et al., 2020). Although it is an incredibly complex genus, genomic studies of *Euphrasia* continue to reveal key insights into the nature of species differences and how hybridization, selfing, and phenotypic plasticity shape the evolution of polyploid taxa.

Given the scale of taxonomic complexity in *Euphrasia*, here we focus on co-occurring species on a single island. Fair Isle, a small island of 768 hectares, is the most remote inhabited island in the British Isles, situated halfway between Orkney (42 km away) and mainland Shetland (39 km away). Fair Isle *Euphrasia* provide an ideal study system due to their isolation from nearby gene flow and the closely intermixed habitats that support different species. The well-characterized island flora of 260 native species and 71 aliens (Quinteros Peñafiel et al., 2017) includes only tetraploid *Euphrasia:* eight *Euphrasia* species and eight putative hybrids (Riddiford et al., 2020). Here we focus on three tetraploid *Euphrasia* species that are widely distributed across the island but differ in their habitat preferences (see Figure 1). *Euphrasia arctica* is a grassland species that has vigorous growth and large flowers that are thought to be highly outcrossing. *Euphrasia foulaensis* is a coastal specialist with thickened leaves and compressed growth. It has small flowers, some of which do not open and are thought to be cleistogamous. *Euphrasia micrantha* is an upland heathland species that is slender. It often has pink-suffused leaves, stems, and flowers, and its very small flowers are highly selfing (Stone, 2013). *E. micrantha* is variable in morphology throughout its range, with the Fair Isle form smaller, usually unbranched, with magenta flowers with a less distinct lower floral "lip." Despite the three species having different habitat preferences, the small scale of the island and fine-scale habitat differences mean species occur in sympatry (*sensu* Mallet et al., 2009) (Figure 1). To place these Fair Isle plants in a broader context, we also include diploid and tetraploid individuals from elsewhere in Great Britain.

In this study, we use field-based observations, a common garden experimental approach, and whole-genome sequencing to understand the nature of species differences in a group of taxonomically complex, sympatric, polyploid *Euphrasia* species. We address the following research questions: (1) is there a genetic component to the morphological differences between species with different habitat preferences? (2) what is the evolutionary history of polyploidization in *Euphrasia*? and (3) what is the landscape of genomic differentiation between sympatric species? To address question 2, we propose a novel k-mer-based analytical approach to infer sub-genome divergence in allopolyploids. Our results show how species differences are maintained over a fine spatial scale despite incomplete reproductive isolating barriers and reveal how genomic approaches can be used to characterize speciation histories of a non-model tetraploid group.

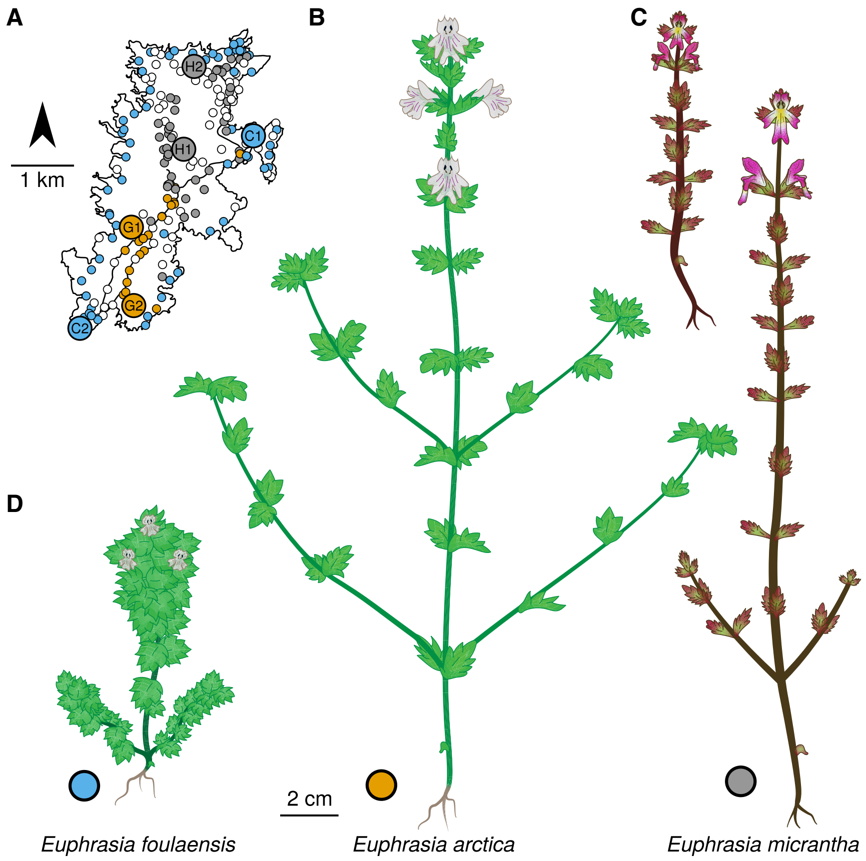

Figure 1. Three Focal *Euphrasia* Species and Their Distributions on Fair Isle.
(A) Geographic distributions of *E. arctica*, *E. foulaensis*, and *E. micrantha* based on a field survey recording 282 presence (colored) or absence (white) census points. Labels indicate the populations from which seeds were sourced and traits were measured.
(B–D) **(B)** *E. arctica* and **(C)** *E. micrantha*. The smaller form is typical for Fair Isle. **(D)** *E. foulaensis*.

RESULTS

Phenotypic Differences between *Euphrasia* Species Are Heritable

To understand morphological differences among Fair Isle *Euphrasia* species, we compared trait variation in the wild (n = 180) and in a common garden experiment (n = 2116). We confirmed that in the wild *E. arctica* is tall (mean height 80.0 mm) and large flowered (mean corolla size 7.6 mm), *E. foulaensis* short (19.7 mm) and smaller flowered (5.9 mm), and *E. micrantha* intermediate in height (40.2 mm) and small-flowered (4.7 mm), with these trait values significantly different between all three species in mixed-effect models ($p < 0.01$; Figure 2A, Supplemental Tables 1 and 2, and Supplemental Text 1). Overall, however, many more traits showed significant differences at the population rather than the species level (Figure 2A, bottom right triangles). Based on multitrait phenotypes, individuals partly clustered by species in a principal component analysis (PCA, Figure 2B), while linear discriminant analysis (LDA) trained on 80% of the data classified all remaining individuals correctly. These results show that species and populations differ in their overall phenotype and in some key traits in natural populations.

To assess whether the phenotypic species differences have a genetic component, we analyzed morphological differentiation in a common garden setting by measuring individuals grown from seeds sourced from the same natural populations as above. Because host identity can affect performance in parasitic *Euphrasia* (Brown et al., 2020), we grew each individual with one of 12 different host species that occur on Fair Isle (Supplemental Table 2). Including host species as a random effect improved the fit of our models in terms of significantly lower Akaike Information Criterion values. Under these common garden conditions, there were fewer observable trait differences than in natural populations. The only trait showing significant differentiation between all species was capsule length, which was high in *E. arctica* (5.8 mm), intermediate in *E. foulaensis* (4.9 mm), and short in *E. micrantha* (3.7 mm) ($p < 0.01$). However, there were still some pairwise species differences, and many pairwise population differences (Figure 2A, top left triangles). Overall, the clustering of different species based on multitrait phenotypes in the common garden (Figure 2C) was much less obvious than in natural populations (Figure 2B). However, using LDA trained on 80% of the data (439 of 549 individuals without any missing data), it was possible to accurately classify 75% of the individuals in the test set (i.e., 82 of 110 individuals were assigned the species from which their seeds had been collected, Figure 2D). The success of LDA classification was high for *E. arctica* (78%) and *E. foulaensis* (75%), which misclassified individuals as the other species 22% of the time, while *E. micrantha* (62% classification success) was most commonly misclassified as *E. foulaensis* (25%). Overall, the presence of species-specific multitrait combinations in a common environment shows that there is a genetic component to the phenotypic species differences, but the lower classification success in a common garden indicates that many trait differences observed in the wild are due to plasticity.

Complex Patterns of Plastid Genome and rDNA Relatedness

We then generated whole-genome sequencing data for 18 *Euphrasia* individuals to investigate the genomics of species differences and polyploid history. Our samples included 12 Fair Isle tetraploids from our three focal species and two tetraploid individuals considered putative hybrids (labeled X1 and X2). The other samples were two tetraploids and four diploids from mainland Britain (see Supplemental Table 3 for details). *De novo* assembly of plastid genomes revealed complex patterns of

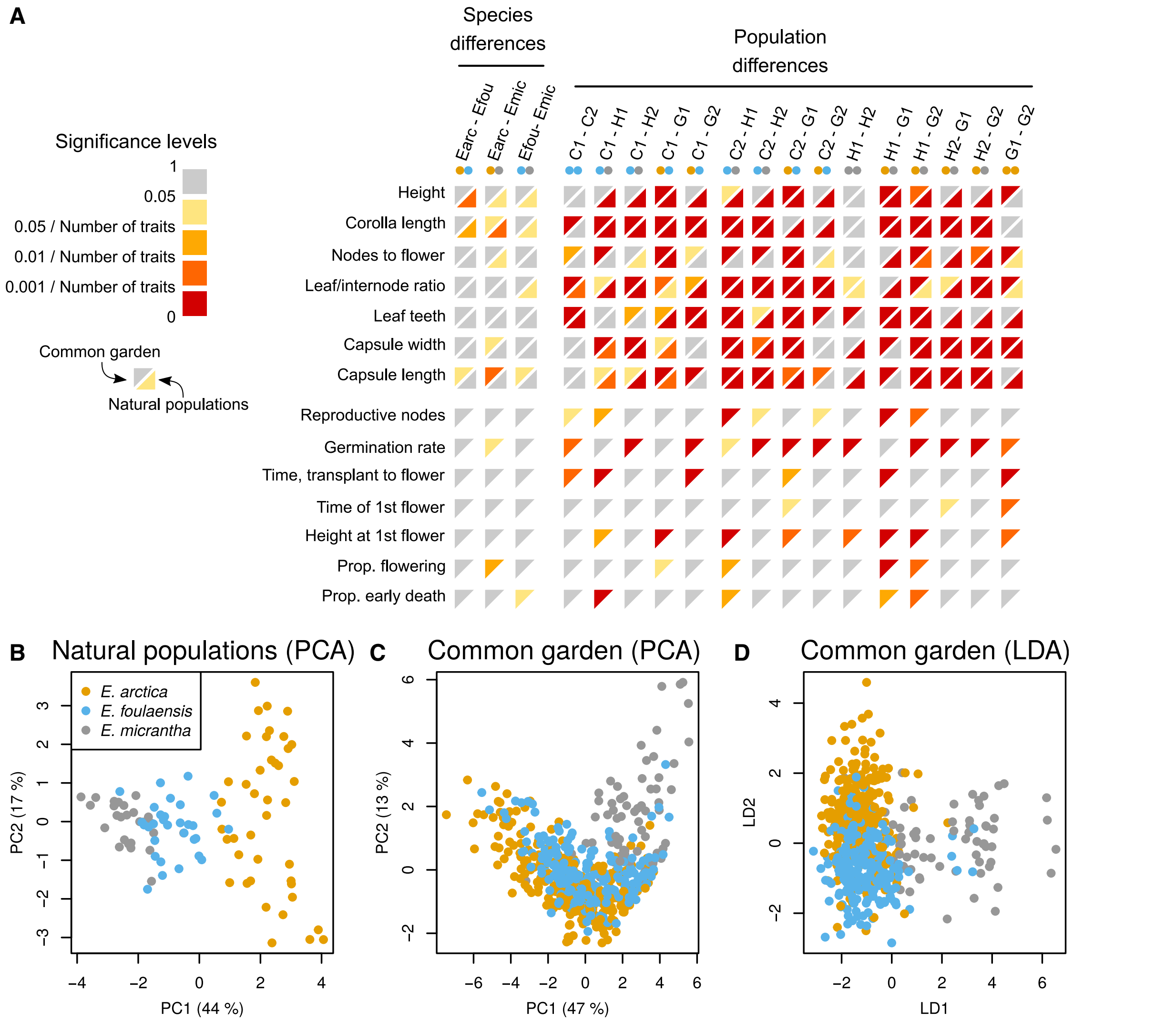

Figure 2. Morphological Trait Differentiation between Three Species of *Euphrasia* in a Common Garden Experiment and in Natural Populations.

(A) Significance levels of trait differences between species (left) and populations (right) from field measurements in natural populations (bottom right triangles) and in the common garden (top left triangles, see Supplemental Text 1 for the magnitudes of differences and Supplemental Table 2 for the means and standard errors). Comparisons within rows are corrected for multiple testing. Within columns, the color scale is the *p* value corrected for the number of traits tested (seven in natural populations and 14 in the common garden). While significant trait differences are rare between species, they are numerous between populations (see Figure 1A or Supplemental Table 1 for population codes).

(B) PCA of trait measurements from natural populations shows separate clusters per species.

(C) PCA of trait measurements from plants grown in the common garden shows little grouping by species.

(D) LDA separates species in the common garden.

plastid haplotype sharing and relatedness. Across samples, plastid genomes were similar in size (144 739–145 009 bp) and in sequence (>99.8% pairwise sequence identity). Fair Isle *Euphrasia* fell into three broad plastid haplogroups, each with 100% bootstrap support in our phylogenetic analysis (Figure 3A). Haplogroup 1 was composed of a mix of Fair Isle *Euphrasia* and mainland British diploid and tetraploid species. Haplogroup 2 was predominantly found in Fair Isle samples, plus the putative hybrid species *Euphrasia rivularis* sampled from England. Haplogroup 3 was exclusively composed of Fair Isle individuals of *E. micrantha*. Within each of these haplogroups, there was variable genetic divergence, with some extremely closely related haplotypes differing by a few SNPs, and some more divergent haplotypes showing structural genetic changes. Furthermore, there was plastid haplotype sharing between co-occurring samples of *E. arctica* and *E. foulaensis* on a roadside in the south of Fair Isle, as well as their putative hybrid (sample X1). Overall, these results indicate that diverse plastid haplotypes are maintained within an island population of *Euphrasia*, with Fair Isle *E. micrantha* differing from intermixed *E. arctica* and *E. foulaensis*.

De novo assembly and comparative analyses of *Euphrasia* rDNA revealed deep divergence between UK diploids and tetraploids, confirming previous results from internal transcribed spacer (ITS) sequencing of a broader taxonomic sample (Wang et al., 2018).

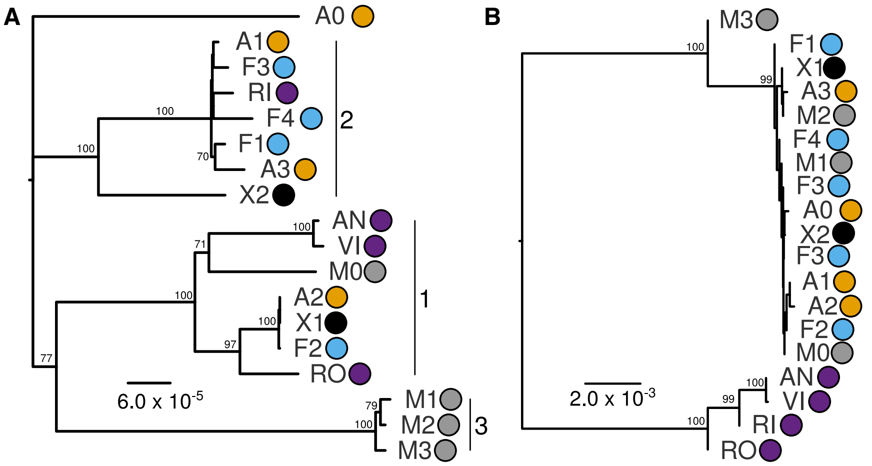

Figure 3. Evolutionary Relationships of British *Euphrasia* Plastid Genomes and rDNA Sequences.

(A) Phylogenetic analysis of plastid genomes performed using a maximum-likelihood approach implemented in IQ-TREE with the K3Pu+F+I substitution model. Haplogroups are indicated by numbered vertical lines.

(B) Phylogenetic analysis of nuclear rDNA sequences using a neighbor-joining approach implemented in Geneious. There were two rDNA haplotypes in sample F3. In both **A** and **B**, colored circles indicate species identity: orange, *E. arctica*; blue, *E. foulaensis*; gray, *E. micrantha*; black, putative hybrid individuals; and purple, diploid species. Scale bars indicate branch lengths. Numbers on branches indicate bootstrap support ≥70%.

Across the 5832-bp rDNA coding region, there was a mean pairwise diploid–tetraploid divergence of 2.1%, but the divergence in the ITS2 region was up to 10.8%. Within tetraploids, however, there was very limited sequence divergence, with >99.5% pairwise sequence identity between Fair Isle samples. rDNA haplotypes were shared between some individuals, such as *E. foulaensis* F2 and *E. arctica* A1, with one sample of *E. micrantha* maintaining a more divergent haplotype. These results support the recent divergence of species and populations, with extensive haplotype sharing particularly among *E. arctica* and *E. foulaensis*.

Polyploid Genome Diversity Inferred with k-mer Analysis Methods

To assess the genomic properties of tetraploid *Euphrasia* without the need for genome assembly, we employed k-mer-based methods. In addition to using KAT, GenomeScope, and Smudgeplots, which visualize polyploid genome variations, we developed mathematical models for the shape of k-mer spectra of auto- and allotetraploids. We implemented these models in a "shiny" app, "Tetmer" (available from GitHub: https://github.com/hannesbecher/shiny-k-mers). Tetmer takes as an input a k-mer spectrum of a single tetraploid (or diploid) individual. In "Autofit" mode, it estimates the population genetic parameters θ (population-scaled mutation rate) and T (divergence time between homoeologous sub-genomes); it also fits the haploid (non-repetitive) genome size and a bias parameter (Vurture et al., 2017) accounting for the width of peaks in the k-mer spectrum. Tetmer can also be used in "Manual" mode to manually adjust the fit or to explore the expected shape of a k-mer spectrum under a certain model and parameter set.

Our approach is detailed in Supplemental Text 2. In brief, the characteristics of genetic variation in an autotetraploid is similar to those of four haploid samples from one panmictic population. The genetic variation within an allotetraploid, however, is more similar to that of two pairs of haploid samples from two diverged populations (between which gene flow ceased a certain time ago). This causes different distributions of genetic diversity that are evident in k-mer spectra. In autotetraploids, most variant sites contain singleton alleles (1/3 or 1/1/2), causing a prominent 1× peak. In allotetraploids, however, variant sites have mainly doubleton alleles (2/2), reflecting sub-genome divergence, and causing a prominent 2× peak.

The k-mer spectra of all tetraploids analyzed showed prominent 2× peaks, clearly indicating allotetraploidy. Smudgeplots confirmed tetraploidy for all these samples (see "genome profiling" in Supplemental Text 3). We estimated the single-copy sequence in a haploid genome to be 185–225 Mb in our samples. This was generally about one-third higher than the "Genome Unique Length" reported by GenomeScope, which aims to exclude paralogous sequences. We then used Tetmer to estimate heterozygosity within and divergence between sub-genomes (Figure 4B and 4C). The heterozygosity estimates were noisy because they depend mainly on the 1× peak in the k-mer spectra (Figure 4A), which is often partly concealed by sequencing errors and contamination. We also estimated heterozygosity from SNPs called relative to a reference genome (discussed below) and by using GenomeScope on the diploids. Both approaches generated results similar to those from Tetmer. The average k-mer-based heterozygosity estimate over all samples was 0.2% and did not differ significantly between diploids and tetraploids (ANOVA, $p_{(df=16)}$ = 0.07). Two samples, RO (*Euphrasia rostkoviana*) and A3 (*E. arctica*), had heterozygosity values considerably higher (1.1% and 0.52%, respectively), which was likely the result of recent outcrossing events in these mixed-mating species.

Finally, we used Tetmer to estimate the divergence between homoeologous sub-genomes of the tetraploids. Because the divergence estimate depends mainly on the relative size of the 2× and 4× peaks, it is more accurate than heterozygosity estimates based on the 1× peak. We found that all tetraploids showed a per-nucleotide sub-genome divergence of about 5%, one to two orders of magnitude higher than the heterozygosities observed (Figure 4C). This was similar to the heterozygosity class "aabb" report by GenomeScope, but Tetmer's estimate takes into account possible ancestral polymorphisms and heterozygosities within sub-genomes. The consistency of sub-genome divergence across samples raises the possibility of a common origin for these polyploids, involving the same or similar parental progenitors.

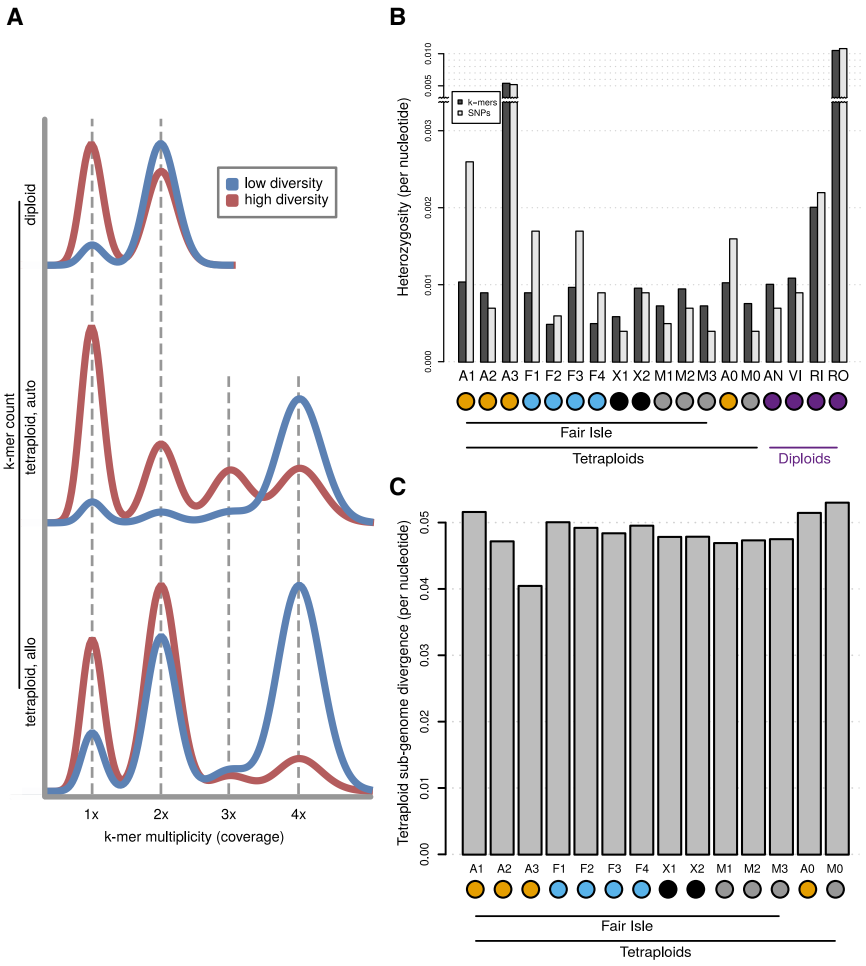

Figure 4. Estimates of Heterozygosity and Sub-genome Divergence in Allotetraploids Based on k-mer Spectra.

(A) Schematic of the shapes of k-mer spectra of diploids, autotetraploids, and allotetraploids. Spectra of low-diversity species are shown in blue and high-diversity species in red. A general feature is that the higher the genetic diversity, the higher the 1× peak. Our app, Tetmer, allows these models to be fitted to empirical k-mer spectra.

(B) Heterozygosity estimates for *Euphrasia* individuals based on k-mers (dark bars) and SNPs (light bars).

(C) Estimates of sub-genome divergence for tetraploid *Euphrasia* individuals.

Extensive Scaffold Homology between Diploids and Tetraploids

To further our understanding of sub-genome divergence and polyploid history, we generated a draft genome assembly of a geographically isolated tetraploid sample of *E. arctica* from North Berwick, Scotland (individual A0). This hybrid assembly from 97× short-read Illumina and 16.7× long-read Pacific Biosciences (PacBio) data comprised 1 009 737 scaffolds, spanning 694 Mb in length. Although fragmented, the assembly was relatively complete, as indicated by the KAT completeness plot (Supplemental Figure 1) and the BUSCO completeness score of 81.7%. We then mapped genome resequencing data of all 18 diploids and tetraploid individuals to our reference assembly and classified scaffolds based on mapping depths. Only 0.1% of scaffolds (1024 scaffolds; 3 Mb) had 4× mapping depth in the tetraploids, as would be expected if they were autotetraploids, providing further support for the allopolyploid nature of the tetraploids. By contrast, 10 644 scaffolds (132 Mb) had a diploid-level (2×) mapping depth across all tetraploids, representing regions where diverged (homoeologous) sequences were assembled into separate scaffolds, which we called the "tetraploid" set. A subset of these tetraploid scaffolds had 2× mapping depth across all samples, representing regions homologous across ploidy levels (the remainder of the tetraploid scaffolds were mostly missing from diploids). We called these 3454 homologous scaffolds (46 Mb) the "conserved" set. The tetraploid and conserved sets formed the basis for subsequent population genomic analyses (Figure 5A). Overall, the consistency in mapping depth patterns between tetraploids suggests widespread sequence homology and possibly a common origin. Diploid–tetraploid homology supports the prediction that a (relative of a) British diploid was a progenitor of British tetraploids (Yeo, 1966), with a second sub-genome contributed by a divergent taxon.

Strong Genetic Structure Despite Low Divergence

We used genetic clustering approaches to understand whether species from contrasting habitats are genetically cohesive. STRUCTURE analyses revealed that Fair Isle *E. micrantha* was a distinct genetic cluster in our sample of natural *Euphrasia* populations. Assuming three genetic clusters (K = 3, Figure 4C), there were clusters corresponding to Fair Isle *E. micrantha*, other Fair Isle taxa, and diploids. The mainland tetraploids (A0 and M0) were found to be admixed between clusters. At higher K values (Supplemental Figure 2), genetic clustering corresponded to broad taxonomic groupings, although *E. arctica* and *E. foulaensis* on Fair Isle were not separated. In PCA, Fair Isle *E. micrantha* was separated from all other species on principal component (PC) 1, while other Fair Isle species were separated from diploids on PC2, with mainland tetraploids in-between these groupings. Overall, these analyses point to divergence between Fair Isle *E. micrantha* and all other taxa being the major axis of divergence among our samples, rather than diploid–tetraploid divergence as found in other genetic analyses of *Euphrasia* (French et al., 2008; Wang et al., 2018).

To better understand how variation is maintained within and between populations and species, we characterized genomic diversity and divergence based on 922 927 high-quality SNPs in the conserved and 2 166 914 SNPs in the tetraploid scaffolds. The per-species estimates of nucleotide diversity (H_e) were considerably higher than individual heterozygosities, ranging from 0.26%

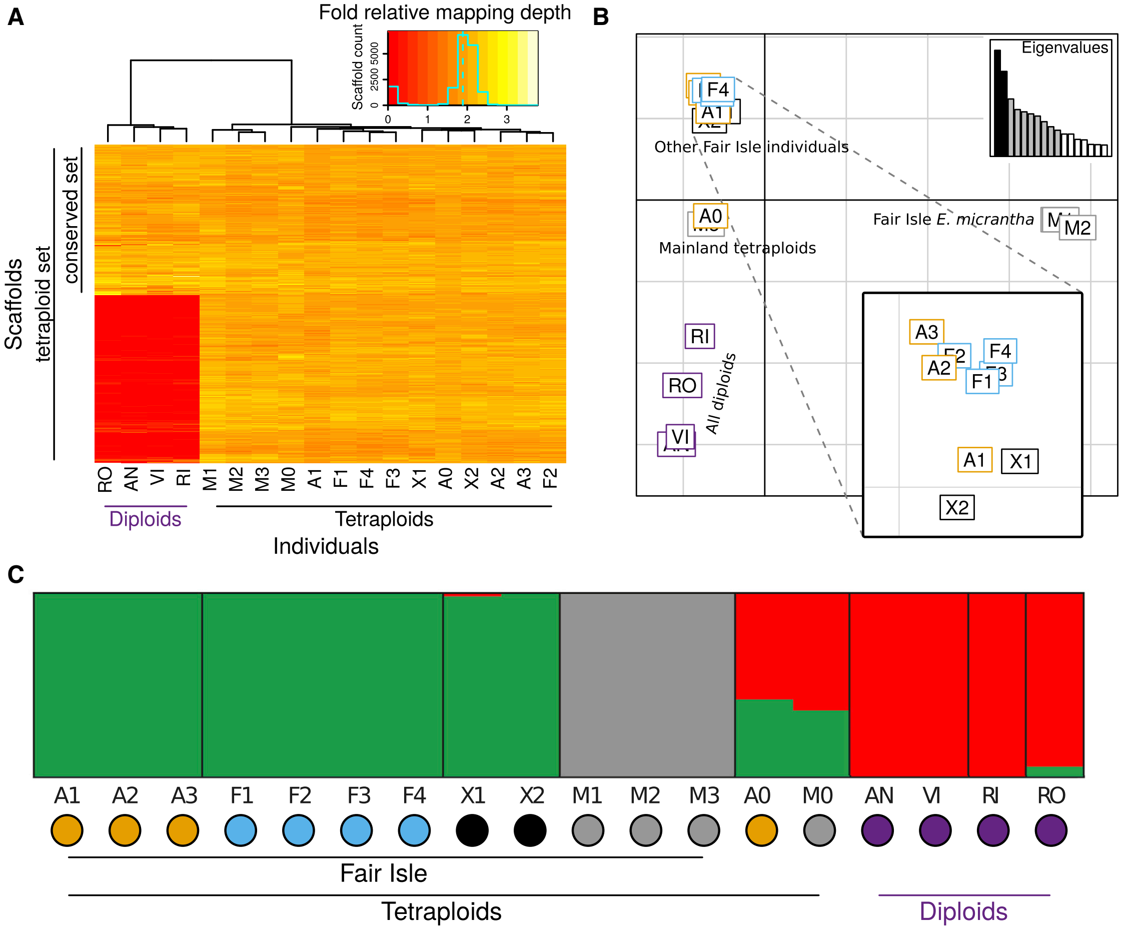

Figure 5. Diploid–Tetraploid Scaffold Homology and the Clustering of *Euphrasia* Populations.
(A) Relative mapping depth in the tetraploid (2× depth in tetraploids) and conserved (2× depth all individuals) scaffold sets. Colors represent mapping coverage (see inset). Tetraploid scaffolds not contained in the conserved set have low mapping depths in diploids, indicative of absence (red).
(B) PCA of genomic data (conserved scaffolds) separates Fair Isle *E. micrantha* individuals from other *Euphrasia*. PC2 separates tetraploids and diploids. The analysis was based on 3454 SNPs, with one SNP per scaffold.
(C) STRUCTURE analysis shows Fair Isle *E. micrantha* as a separate genetic cluster. Analysis based on the same SNP data as in **(B)**. The plot shows the results for three genetic clusters ($K = 3$), with individuals on the x-axis and admixture proportions on the y-axis. The mainland tetraploids, M0 and A0, are inferred to be admixed. Colored outlines in **B** and dots in **C** represent taxa to match Figure 3.

to 0.32% on Fair Isle and from 0.31% to 0.43% when all non-hybrid individuals were considered. The total nucleotide diversity over all individuals (and species, H_t) was 0.53%. A one order of magnitude difference between heterozygosity and population nucleotide diversity was observed even on Fair Isle (top of Figure 6A), where the aggregation of populations characterized by geographic isolation by distance is unlikely to play a role. Instead, these differences are likely to be the result of high levels of selfing. The per-nucleotide net divergence between species ($\pi^{(net)}$ or d_a) ranged from 0.07% (*E. arctica–E. foulaensis*) to 0.32% (*E. arctica–E. micrantha*) on Fair Isle, while this was lower (0.07%–0.24%) in the broader comparison (both comparisons excluded hybrid individuals). With the exception of comparisons including Fair Isle *E. micrantha*, these estimates of between-species divergence were lower than the diversity found within each species (Figure 6A and 6B).

Our finding of clear genetic clusters but low divergence suggests that species may be recent; however, these analyses do not indicate the amount of gene flow. We tested the extent of allele frequency differences, as measured by the fixation index (F_{ST}), and found considerable genetic structure between Fair Isle *E. arctica* and *E. foulaensis*, on one hand, and *E. micrantha* on the other. In comparisons to *E. micrantha*, genetic structure was high, with mean F_{ST} values of 0.44 (*E. arctica–E. micrantha*) and 0.43 (*E. foulaensis–E. micrantha*), while 11% of scaffolds had $F_{ST} > 0.8$ and 38% of scaffolds had $F_{ST} > 0.5$. Both F_{ST} distributions showed very similar shapes (Figure 6C). Genetic structure was lower in the comparison between *E. arctica* and *E. foulaensis*, with a mean F_{ST} of 0.21, and only a minority of scaffolds with high values (0.11% > 0.8; 7.4% > 0.5). On a larger scale, including all non-hybrid individuals and treating the diploids as one group, the differentiation between species was lower and more similar across comparisons, with mean F_{ST} values ranging from 0.18 to 0.37, with the largest value for the comparison *E. foulaensis–E. micrantha* (Figure 6C, bottom). This is in line with previous *Euphrasia* studies that show genetic

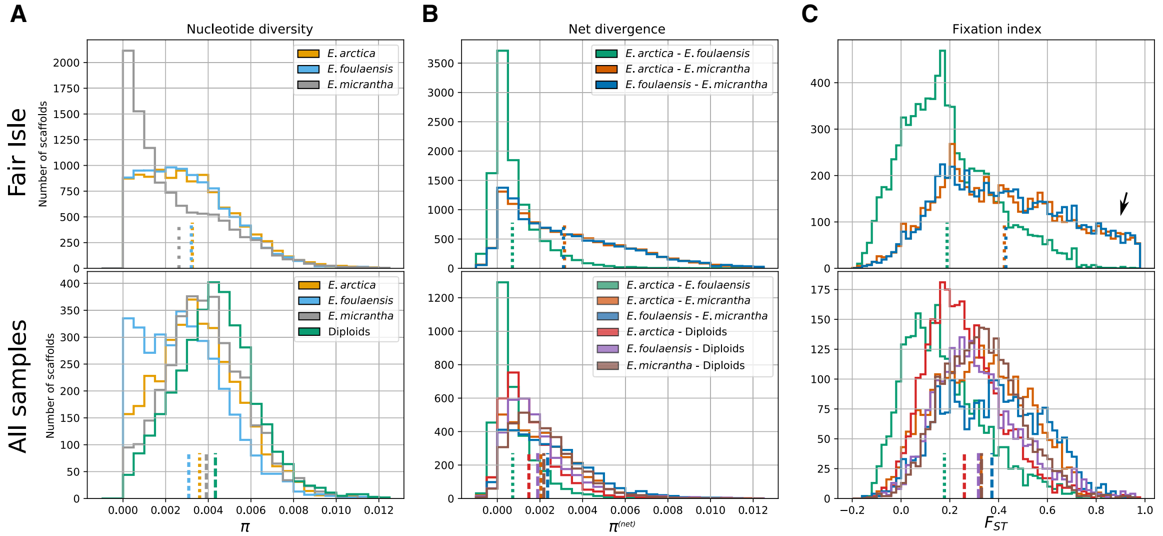

Figure 6. Considerable Genetic Structure with Little Differentiation between *Euphrasia* Species.

Histograms show per-scaffold statistics with population means indicated by dashed lines at the bottom of each graph. The top row shows results based on the tetraploid data set for non-hybrid individuals from Fair Isle, while the bottom row is based on the conserved set, and it includes all non-hybrid individuals (with all diploids treated as one group).

(A) Nucleotide diversity on Fair Isle is slightly lower in *E. micrantha* than in *E. arctica* and *E. foulaensis*, and overall, these values are considerably higher than per-individual heterozygosity estimates (Figure 4B), consistent with high levels of selfing.

(B) On Fair Isle, the net divergence shows very similar patterns in the comparisons involving *E. micrantha*, while divergence between *E. arctica* and *E. foulaensis* is much lower. With wider sampling, the divergence between *E. micrantha* and the other species is lower, an indication that mainland *E. micrantha* carries alleles shared with the other species. The net divergences estimated here are of similar magnitude or lower than the nucleotide diversities shown in **(A)**.

(C) Although net divergence tends to be low between species of *Euphrasia*, the fixation index can be extreme for some scaffolds, for example, Fair Isle comparisons including *E. micrantha* (arrow). This genetic differentiation disappears when samples from additional populations are included, indicating that allelic frequency divergence is greater at the population level than at the species level.

variation to be structured by region more than by species (Kolseth and Lönn, 2005; French et al., 2008).

Genetic Relationships Cannot Be Explained by a Single Tree

To characterize genome-wide relationships of populations and species, we generated trees from thousands of genomic scaffolds and used these to produce a consensus tree indicative of the relationships of populations and species. The ASTRAL consensus tree had a high posterior probability ($PP \geq 0.97$) for all nodes, except for the clade of mainland polyploids ($PP = 0.82$; Figure 7A). It did not generally show grouping by species; however, the diploids and Fair Isle *E. micrantha* were placed in distinct clades. The node ages, which are given in coalescent time, were comparatively young, with the oldest being 1.39 (in generations scaled by $2N_e$, corresponding to a coalescence probability of 75%). Such recent divergence gives many opportunities for incomplete lineage sorting. To better characterize the diverse genealogical histories present in the genome, we inspected the individual gene trees. Across 3454 scaffolds there was no clear congruence between individual trees and no general groupings by species, except some clustering of Fair Isle individuals of *E. micrantha* (M1–3; Figure 7B). This suggests that, while broadscale relationships reflect the divergence of Fair Isle *E. micrantha* from other tetraploids, and while diploids cluster separately, there is substantial gene tree incongruence and phylogenetic complexity.

To further categorize and quantify the different trees present in the genome, and to detect possible routes of gene flow, we conducted topology weighting with Twisst (Martin and van Belleghem, 2017). This approach compares individual trees to a set of reference topologies, and here we tested the three possible unrooted topologies of four groups (each tetraploid species, excluding hybrids, and all diploids as an outgroup). The most common topology placed *E. arctica* and *E. foulaensis* as closest relatives and *E. micrantha* on a separate branch, while both alternative topologies had lower weights (Figure 7C). However, few of the 3454 per-scaffold trees matched one of the three reference topologies (419, 15, and 10, respectively). The topology mean weights were similar when mainland tetraploids were included (47.2%, 25.4%, and 27.5%, with 105, 3, and 1 tree matching the reference topologies entirely). For both Fair Isle and the wider sampling, the topology with *E. arctica* and the diploids as closest relatives had a somewhat higher weight than the alternative (*E. micrantha* and *E. arctica* as closest relatives), suggesting possible gene flow between *E. arctica* and the diploids, or between *E. micrantha* and *E. foulaensis*. We tested this with Patterson's D statistic using the diploids as an outgroup and found that D deviated significantly from zero ($D = 0.02$, $p = 0.005$) only when the mainland tetraploids were included. This suggests gene flow between (mainland) *E. arctica* and the diploids, with an admixture fraction measured as F_G of 2.5% (Green et al., 2010). This value should be interpreted

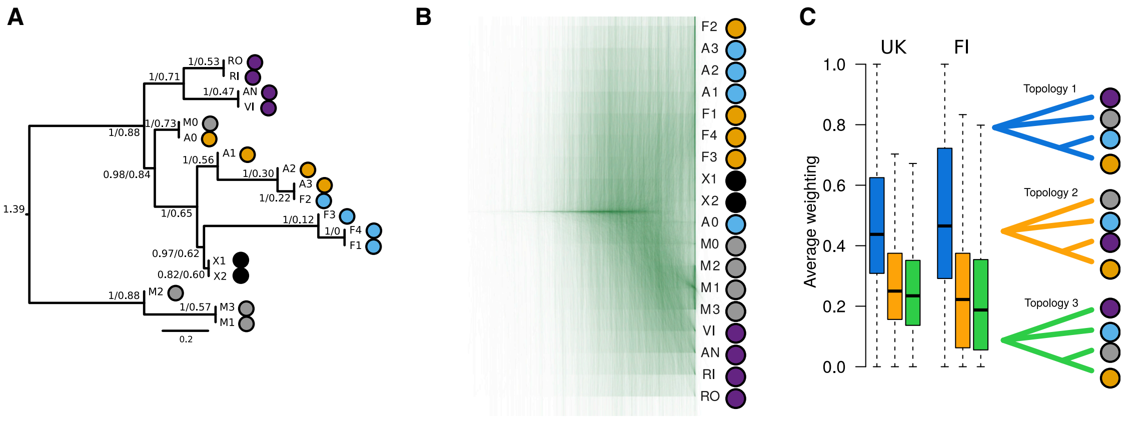

Figure 7. Complex Evolutionary Relationships and Extensive Discordance in *Euphrasia*.

(A) ASTRAL consensus tree based on 3454 per-scaffold trees from the conserved scaffold set. The numbers are a node's posterior probability and its age in coalescent units. The tree is rooted at the longest branch (between Fair Isle *E. micrantha* and all other individuals).

(B) Overlaid gene trees of the conserved scaffold set show no single clear species relationship.

(C) Topological weighting of 3454 trees of all Fair Isle non-hybrids (FI) and all non-hybrid tetraploids (UK), both using the diploids as an outgroup, carried out with Twisst. While blue topology 1 tends to receive the highest weighting, few trees have a very high weight (near 1) for any one topology. The two alternative topologies receive similar levels of support. Colored dots represent taxa to match Figure 3.

as additional admixture on top of any background level between all pairs of species.

DISCUSSION

Adaptive divergence is a major driver of speciation but remains poorly studied in polyploid organisms. In the present study, we use whole-genome sequencing in combination with common garden experimental approaches to understand the nature of species differences in a complex tetraploid group. We focus on an isolated island system where we can characterize genome-wide diversity and divergence of three differentially adapted eyebright species in sympatry. In our focal species, we find that phenotypic differences are underpinned by genetic differences, providing support for the recognition of species differences in this taxonomically complex group. Building upon this finding, in the discussion that follows, we first consider the technical aspects of polyploid data analysis and the new insights we have gained into polyploid evolution, before discussing the landscape of genomic differentiation and adaptation in *Euphrasia*.

Polyploid Genome Dynamics

Polyploid-aware analyses are key to understanding the evolutionary histories of British eyebrights and many other postglacial groups such as octo- and dodecaploid *Cerastium* (Brysting et al., 2007), tetra-hexaploid species of *Silene* (Popp et al., 2005), the highly variable ploidy *Primula* section *Aleuritia* (Guggisberg et al., 2006), and *Dactylorhiza* orchids (Brandrud et al., 2019). While such analyses used to involve the cloning of homoeologous sequences and comparisons of tree topologies, the use of whole-genome data without assembly is becoming increasingly popular. Several k-mer-based methods are now used routinely in genome-sequencing projects of any complexity. For instance, KAT (Mapleson et al., 2016) and GenomeScope (Vurture et al., 2017) can be used to estimate the heterozygosity of diploids and to characterize polyploid k-mer spectra, while Smudgeplots (Ranallo-Benavidez et al., 2020) can be used to determine the ploidy of a sample. However, these programs do not explicitly model the mechanism underpinning genetic diversity in allopolyploid genomes. Tetmer partly fills this gap by explicitly assuming (and allowing researchers to estimate) divergence between allotetraploid sub-genomes, with this corresponding to divergence over time between isolated populations. Tetmer can also be used to analyze autotetraploid and diploid genomes and to visualize the expected k-mer spectrum given a set of parameters. Similar to other k-mer methods, it works best on short-read data of suitable coverage (haploid peak >15×) generated from a single individual, ideally using contaminant-free samples and PCR-free sequencing libraries.

Using Tetmer on k-mer spectra of *Euphrasia* revealed considerable between-individual variation in heterozygosity within species, indicative of a mixed-mating system. This approach of analyzing divergence within an individual proves complementary to comparative genomics between diploids and tetraploids, which revealed a set of scaffolds shared between diploids and tetraploids. This points to a (relative of a) British diploid acting as a parental progenitor to the British tetraploids, with the origin of the other, highly divergent, sub-genome (~5% divergence) unknown.

The likely absence in Britain of the second diploid ancestor—the Wang et al. (2018) barcoding study failed to find the tetraploid rDNA haplotype in British diploids—and distinct genetic clustering of the diploids suggest that allopolyploidy in *Euphrasia* is not recent. This proves problematic for dating the polyploidy event(s), which would require extant but genetically isolated diploid relatives (Doyle and Egan, 2010). A further complication is that ongoing gene flow, evidenced by plastid sharing and the presence of natural diploid–tetraploid hybrids (Yeo, 1956), blurs the split between diploids and tetraploids. Polyploid species tend to go through a process of diploidization, involving the reduction in chromosome number,

homoeologous rearrangements, and gene losses, leaving paleopolyploids with prepolyploidy genome sizes (Mandáková and Lysak, 2018). This process does not seem to progress at equal pace in different polyploids. For instance, *Brassica napus*, a recent allopolyploid formed 7500–12 500 years ago with extant diploid relatives, experienced extensive homoeologous rearrangements (Chalhoub et al., 2014). By contrast, teff (*Eragrostis tef*, VanBuren et al., 2020) formed 1.1 million years ago without known diploid ancestors, and *Capsella bursa-pastoris* formed about 100 000 years ago (Douglas et al., 2015; Kryvokhyzha et al., 2019) do not show large-scale genomic rearrangements. Thus, homoeologous exchange between sub-genomes and rearrangements has major effects on interspecies gene flow, divergence, and ultimately adaptation and speciation. These questions of sub-genome structure and genomic rearrangements will be addressed with an improved genome assembly, generated by the Darwin Tree of Life project (https://www.darwintreeoflife.org), and further population sequencing.

Landscape of Genomic Differentiation and Speciation History

Studies of speciation genomics may gain valuable insights by investigating taxa at different stages of the speciation trajectory, from the earliest stages of divergence to genetically differentiated species with strong reproductive barriers (Via, 2009; Twyford et al., 2014). We have shown that three species of eyebright adapted to contrasting environments are characterizsed by a gradient of genomic differentiation, with grassland *E. arctica* and coastal *E. foulaensis* closely related, while the dry heathland specialist *E. micrantha* is genetically and morphologically more distinct. This parallels other genomic studies of adaptive divergence where species boundaries show different degrees of permeability, underpinned by different reproductive isolating barriers (Peccoud et al., 2009; Dasmahapatra et al., 2012; Nosil et al., 2012; Renaut et al., 2012; Roda et al., 2013). Reproductive isolation between *E. arctica* and *E. foulaensis* is incomplete and there is evidence of ongoing gene flow. However, the long tail in the pairwise F_{ST} distribution reveals many differentiated scaffolds, which may represent important regions of divergence involved in adaptation to harsh coastal environments (Lowry et al., 2008; Lyu et al., 2018; Dong et al., 2020) and the maintenance of species identities (Nosil et al., 2009; Ravinet et al., 2017). By contrast, *E. micrantha* shows genome-wide divergence from the other two taxa, despite a similar degree of geographic co-occurrence. The high selfing rate of *E. micrantha* (Stone, 2013) and intrinsic postzygotic barriers (genomic incompatibilities) are likely to underlie reproductive isolation. However, our genomic analyses suggest that the genetic distinctness of *E. micrantha* may be a feature of the Fair Isle population rather than the species as a whole. This could be a consequence of regional differentiation caused by strong drift and the local fixation of genomic incompatibilities, local hybridization, or polytopic origins as seen in other polyploids (Schwarzbach and Rieseberg, 2002; Soltis et al., 2004; Ainouche et al., 2012; Lowe and Abbott, 2015).

Our data provide insights into the enigmatic relationships of eyebrights, showing that their speciation history has been shaped by allopolyploidy with consistent sub-genome divergence, that overall species divergence is extremely shallow, and no single phylogenetic tree can represent their evolutionary history. Genome-scale data for adaptive radiations have revealed many groups characterized by reticulate evolution, and further characterization of this reticulation is a main focus of speciation biology (Rieseberg et al., 2003; Cui et al., 2013; Malinsky et al., 2018; Sun et al., 2018; Feng et al., 2019). Hybridization, followed by adaptive introgression, is advantageous as it substantially reduce the waiting times for new variants to evolve. Similar to many other rapid radiations, the relationships of tetraploid eyebrights are reticulate, and reproductive isolation between them is incomplete, comparable to tetraploid species of *Arabidopsis* (Jørgensen et al., 2011). In such settings, it is easy for adaptive variants to cross species boundaries (Morjan and Rieseberg, 2004), generating novel phenotypic and genic combinations, which may lead to differential adaptation and reproductive isolation (Pardo-Diaz et al., 2012). Partial selfing may exacerbate this, as is seen in *Epipactis* orchids (Squirrell et al., 2002). This diversification process, termed ''combinatorial speciation'' (Marques et al., 2019), is likely to underlie adaptation and the formation of taxonomic complexity in the young, and thus mutation-limited, group of tetraploid *Euphrasia*.

METHODS

Phenotypic Differentiation

Natural Populations

To establish the extent of phenotypic species differences, we measured morphological traits for *E. arctica*, *E. foulaensis*, and *E. micrantha* in natural populations on Fair Isle. For each of two populations per species (Supplemental Table 1), we measured 30 individual plants in the field at a single census point. The set of seven traits that we measured is commonly used in *Euphrasia* identification (Metherell and Rumsey, 2018) and includes plant height, corolla length, number of nodes below the first flower, length ratio of the internode below the first flower and the leaf subtending the first flower, number of leaf teeth, capsule width, and capsule length. All length measurements were made in millimeters, to the nearest 0.1 mm, using digital calipers.

Common Garden Experiment

We obtained seeds from wild-collected and open-pollinated plants, and pooled seeds within a population (Supplemental Table 1). We then placed seeds in individual pots filled with a peat-free soil mix, which is a bark-based substrate of neutral pH (RGBE1), in December. We set up 4320 pots, corresponding to three species of *Euphrasia* with two replicate populations, 12 different host species (see below), and 60 replicates for each combination. These pots were kept outside at the Royal Botanic Garden Edinburgh (RGBE). We recorded germination daily from the beginning of April and supplied *Euphrasia* with host plants (with seeds sourced from Fair Isle and commercial seed suppliers, see Supplemental Table 3) at intervals of about 2 weeks. At the time of first flowering, we measured the same traits as in natural populations. At the end of September (or at the time of death), for each plant, we recorded its final height, number of reproductive nodes (a measure of fitness), and length and width of one capsule. We also recorded which plants had died before the end of the experiment.

Morphological Trait Analysis and Data Visualization

Trait differences were assessed with linear mixed-effect models (R version 3.6.1 package lme4, https://github.com/lme4/lme4/). In the common garden study, host species was used as a random effect, and transplant time was included as a covariate if the model's log likelihood improved significantly. When analyzing trait differences between species, genotype was included as a (nested) random effect. The significance of trait differences between populations or species was assessed with a general linear hypothesis test (function ''glht'') as implemented in the R package

"multcomp" (version 1.4-10). The *p* values of trait differences were corrected for multiple tests at the level of each trait (rows in Figure 2A).

To visualize the individual clustering by phenotype, we carried out PCA with R's built-in function "princomp." To assess how well individuals could be classified into species, we ran LDAs, function "lda" of the R package "MASS," version 7.3-51.4). For plotting, we ran "lda" without cross-validation and used the function "predict" to obtain two scores for each individual, which were plotted in two dimensions.

Genomic Sequencing and Analyses

Sample Processing and Sequencing

We collected individual *Euphrasia* plants in the field into desiccating silica gel. After grinding samples with a tissue mill using ceramic beads, we extracted DNA using the Qiagen DNeasy Plant Mini Kit following the manufacturer's instructions. For the *E. arctica* reference sample, DNA extraction was performed by Amplicon Express using their in-house high-molecular-weight DNA extraction protocol.

We generated short-read Illumina sequence data and long-read PacBio data for a range of *Euphrasia* samples. Full details of the sequencing protocols and specifications are provided in Supplemental Table 4.

Plastid Genome and rDNA Analyses

Plastid genomes were assembled from paired Illumina reads of each *Euphrasia* sample using NOVOplasty. Assembled plastids were manually curated and edited to give a standard order of the large single copy, inverted repeat 1, small single copy, and inverted repeat 2 using Geneious v.11.1. Genome annotation of *E. arctica* A1 was performed using DOGMA (Wyman et al., 2004) with manual editing and curation, and annotation was carried over to other samples using the "annotate from database" option in Geneious. Plastid genomes were aligned using MAFFT. Phylogenetic analyses were performed using IQ-TREE (Nguyen et al., 2014), with the best evolutionary model inferred using model fitting and model assessment based on Bayesian information criterion, and the level of branch support inferred via 1000 rapid bootstrap replicates. To help infer the direction of evolutionary change, we included an unpublished plastid assembly of the divergent *E. antarctica* (A.D.T. and R. Ness, unpublished data) in the initial alignment and tree building. We used this sample to root the phylogeny, and subsequently removed the long branch for better tree visualization. The final alignment of British *Euphrasia* samples included 144 899 constant sites, 174 parsimony-informative sites, and 133 singleton sites.

rDNA was assembled from the same read data as described above, except that we set the expected assembly size to 9000–20 000 bp and used a 1380-bp seed sequence of the rDNA cluster that was obtained from a run of the RepeatExplorer pipeline (Novák et al., 2013). The assembler produced variable results, with some species having fully assembled circularized arrays, while some had multiple contigs. Initial comparisons of assembled arrays suggested that some taxa were not alignable outside the rDNA coding region (results not shown). We therefore trimmed assemblies to the 5.8-kb rDNA coding region (comprising 18S, ITS1, 5.8S, ITS2, 26S). Pairwise alignments were performed with MAFFT, and a neighbor-joining tree was constructed using Geneious.

k-mer Analyses

We modeled the shape of k-mer spectra based on the infinite alleles model. Diploids and autotetraploids were treated as samples of two or four genomes from a single population. Allotetraploids were treated as two samples from each of the two diverged populations. The corresponding formulae were derived using the block-wise site-frequency spectrum framework (Lohse et al., 2011, 2016). Supplemental Text 2 describes this in more detail.

All models are implemented in our app, Tetmer, which can be used to fit parameters to k-mer spectra. This app is available from GitHub (https://github.com/hannesbecher/shiny-k-mers).

Mapping and Variant Analysis

We generated a reference assembly of *E. arctica* from Illumina paired-end and PacBio data using the hybrid assembler SPAdes (Bankevich et al., 2012). The assembly was polished with ntEdit (Warren et al., 2019). The k-mer completeness of the assembly, compared with the sequencing data, was assessed with the k-mer analysis toolkit (Mapleson et al., 2016). Inspection with blobtools (Laetsch and Blaxter, 2017) revealed that there was no contamination at the indicated sequencing depths, and the CG content was similar to that of *Euphrasia* DNA. We removed all scaffolds with an average mapping depth <40× (inspection with blobtools had shown that this threshold separated contaminations from target scaffolds).

We mapped short-read data generated from natural populations to the reference using BWA-MEM (https://arxiv.org/abs/1303.3997v2), and we computed per-scaffold mapping depths. These computations were automated using Snakemake pipelines (Köster and Rahmann, 2012). We then compared the mapping depth information across individuals. Two sets of scaffolds were selected for variant calling: the conserved dataset, which comprised 3454 scaffolds with disomic coverage in all individuals, and the tetraploid set, which comprised the conserved set and 7189 more scaffolds restricted to tetraploid individuals (totaling 10 643 scaffolds). All other scaffolds of the reference were concatenated and maintained in the assembly to avoid non-specific mapping of reads from diverged regions to our scaffold set. We called variants with freebayes (https://arxiv.org/abs/1207.3907) separately for the conserved and tetraploid scaffold sets. In the resulting VCF files, we used only biallelic SNPs with a quality value greater than 100. The VCF files were handled interactively in jupyter lab (https://github.com/jupyterlab) using the scikit-allel package (https://doi.org/10.5281/zenodo.3238280). An HTML report of the analyses is included in the Zenodo archive published alongside this article (https://doi.org/10.5281/zenodo.3774489).

Analysis of Population Structure

We used two complementary approaches to infer population structure. To avoid spurious signals from sites in linkage disequilibrium in these analyses, we selected one random polymorphism per scaffold from the "conserved" scaffold set. We then carried out PCA as implemented in the adegenet package version 2.1.2 (Jombart and Ahmed, 2011) and ran FastStructure (Raj et al., 2014), with *K* values ranging from 1 to 10. As FastStructure may underestimate the extent of admixture, we then analyzed the same dataset in STRUCTURE (Pritchard et al., 2000) using a subset of *K*values (K = 1–5) that were deemed to be most likely. STRUCTURE was run with the admixture model using three replicates per *K* value, with 100 000 burn-in generations followed by 100 000 MCMC generations. The optimal number of clusters was inferred using the ad hoc statistic delta *K* (Evanno et al., 2005). We combined multiple STRUCTURE runs in CLUMPP (Jakobsson and Rosenberg, 2007) and visualized STRUCTURE plots using a custom approach.

Per-Scaffold Trees

We generated per-scaffold unrooted neighbor-joining trees based on pairwise estimates of net-nucleotide divergence between individuals using the nj function of the R package ape v.5.3 (Paradis and Schliep, 2018). These trees were used for a number of downstream analyses, as follows. We generated a consensus tree using ASTRAL (Zhang et al., 2018). We rooted the tree at the longest branch, which connected Fair Isle *E. micrantha* to all the other samples. We then performed topology weighting as implemented in the package Twisst (Martin and van Belleghem, 2017). As candidate topologies, we used all three possible trees with four groups (diploid outgroup and one group per species of *Euphrasia*). We ran Twisst twice, once on the Fair Isle samples, and once on the mainland tetraploids. Finally, we carried out two sets of ABBA-BABA tests with Dsuite (Malinsky et al., https://doi.org/10.1101/634477) using all diploids as the outgroup. Firstly, we used all non-hybrid individuals from Fair Isle, grouped by species, and secondly, we added the mainland tetraploids according to their species.

ACCESSION NUMBERS

Sequence data from this article can be found in the Sequence Read Archive (SRA) under the accession numbers listed in Supplemental Table 4. Additional scripts and data can be found in the Zenodo repository with the https://doi.org/10.5281/zenodo.3774489. The genome assembly for *E. arctica* can be found under GenBank accession no. JACOMQ000000000.

SUPPLEMENTAL INFORMATION

Supplemental Information is available at *Plant Communications Online*.

FUNDING

This work was funded by NERC grants (NE/R010609/1; NE/L011336/1; NE/N006739/1) awarded to A.D.T.

AUTHOR CONTRIBUTIONS

H.B. and A.D.T. designed the experiments. H.B., A.D.T., G.P., and M.R.B. set up the experiments. All authors collected the data. C.M. confirmed the species identifications. N.J.R. provided valuable support on Fair Isle. H.B. analyzed the data. H.B. and A.D.T. wrote the manuscript. All authors commented on and approved the manuscript.

ACKNOWLEDGMENTS

We are extremely grateful to the Fair Isle Bird Observatory for providing accommodation and infrastructure, and the RBGE for providing plant growing facilities. We thank Edinburgh Genomics for generating Illumina data and the Centre for Genomic Research at the University of Liverpool for generating PacBio data. We also wish to thank Molly Carter and Hazel Metherell for assistance in the field, Cristina Rosique for help in setting up the common garden experiment, and Mabon Elis for help with preparing Figure 1. We thank Richard Ennos and Simon Martin, who commented on an earlier version of the manuscript. We also thank Kamil Jaron for supplying the Smudgeplots of the four diploids. No conflict of interest declared.

Received: April 28, 2020
Revised: July 24, 2020
Accepted: August 27, 2020
Published: September 1, 2020

REFERENCES

Abbott, R.J., and Brochmann, C. (2003). History and evolution of the arctic flora: in the footsteps of Eric Hultén. Mol. Ecol. **12**:299–313.

Ainouche, M.L., Fortune, P.M., Salmon, A., Parisod, C., Grandbastien, M.-A., Fukunaga, K., Ricou, M., and Misset, M.-T. (2009). Hybridization, polyploidy and invasion: lessons from *Spartina* (Poaceae). Biol. Invasions **11**:1159–1173.

Ainouche, M., Chelaifa, H., Ferreira, J., Bellot, S., Ainouche, A., and Salmon, A. (2012). Polyploid evolution in *Spartina*: dealing with highly redundant hybrid genomes. In Polyploidy and Genome Evolution, P.S. Soltis and D.E. Soltis, eds. (Berlin, Heidelberg: Springer Berlin Heidelberg), pp. 225–243.

Baduel, P., Quadrana, L., Hunter, B., Bomblies, K., and Colot, V. (2019). Relaxed purifying selection in autopolyploids drives transposable element over-accumulation which provides variants for local adaptation. Nat. Commun. **10**:5818.

Baldwin, B.G., and Sanderson, M.J. (1998). Age and rate of diversification of the Hawaiian silversword alliance (Compositae). Proc. Natl. Acad. Sci. U S A **95**:9402–9406.

Bankevich, A., Nurk, S., Antipov, D., Gurevich, A.A., Dvorkin, M., Kulikov, A.S., Lesin, V.M., Nikolenko, S.I., Pham, S., Prjibelski, A.D., et al. (2012). SPAdes: a new genome assembly algorithm and its applications to single-cell sequencing. J. Comput. Biol. **19**:455–477.

Bernasconi, G., Antonovics, J., Biere, A., et al. (2009). Silene as a model system in ecology and evolution. Heredity (Edinb) **103**:5–14.

Böhle, U.R., Hilger, H.H., and Martin, W.F. (1996). Island colonization and evolution of the insular woody habit in *Echium* L. (Boraginaceae). Proc. Natl. Acad. Sci. U S A **93**:11740–11745.

Brandrud, M.K., Baar, J., Lorenzo, M.T., Athanasiadis, A., Bateman, R.M., Chase, M.W., Hedrén, M., and Paun, O. (2019). Phylogenomic relationships of diploids and the origins of allotetraploids in *Dactylorhiza* (Orchidaceae). Syst. Biol. **69**:91–109.

Brochmann, C., Brysting, A.K., Alsos, I.G., Borgen, L., Grundt, H.H., Scheen, A.-C., and Elven, R. (2004). Polyploidy in arctic plants. Biol. J. Linn. Soc. **82**:521–536.

Brown, M.R., Frachon, N., Wong, E.L.Y., Metherell, C., and Twyford, A.D. (2020). Life history evolution, species differences, and phenotypic plasticity in hemiparasitic eyebrights (*Euphrasia*). Am. J. Bot. **107**:456–465.

Brysting, A.K., Oxelman, B., Huber, K.T., Moulton, V., and Brochmann, C. (2007). Untangling complex histories of genome mergings in high polyploids. Syst. Biol. **56**:467–476.

Campbell, C.R., Poelstra, J.W., and Yoder, A.D. (2018). What is Speciation Genomics? The roles of ecology, gene flow, and genomic architecture in the formation of species. Biol. J. Linn. Soc. **124**:561–583.

Chalhoub, B., Denoeud, F., Liu, S., Parkin, I.A.P., Tang, H., Wang, X., Chiquet, J., Belcram, H., Tong, C., Samans, B., et al. (2014). Early allopolyploid evolution in the post-Neolithic *Brassica napus* oilseed genome. Science **345**:950–953.

Cheng, F., Wu, J., Cai, X., Liang, J., Freeling, M., and Wang, X. (2018). Gene retention, fractionation and subgenome differences in polyploid plants. Nat. Plants **4**:258–268.

Clausen, J., and Hiesey, W.M. (1958). Experimental Studies on the Nature of Species, Volume IV: Genetic Structure of Ecological Races (Washington (DC): Carnegie Institution of Washington).

Clausen, J., Keck, D.D., and Hiesey, W.M. (1940). Experimental Studies on the Nature of Species, Volume I: Effect of Varied Environments on Western North American Plants (Washington (DC): Carnegie Institution of Washington).

Clausen, J., Keck, D.D., and Hiesey, W.M. (1948). Experimental Studies on the Nature of Species, Volume III: Environmental Responses of Climatic Races in Achillea (Washington (DC): Carnegie Institution of Washington).

Cui, R., Schumer, M., Kruesi, K., Walter, R., Andolfatto, P., and Rosenthal, G.G. (2013). Phylogenomics reveals extensive reticulate evolution in *Xiphophorus* fishes. Evolution (N. Y). **67**:2166–2179.

Danley, P.D., Markert, J.A., Arnegard, M.E., and Kocher, T.D. (2000). Divergence with gene flow in the rock-dwelling cichlids of Lake Malawi. Evolution (N. Y). **54**:1725–1737.

Dasmahapatra, K.K., Walters, J.R., Briscoe, A.D., Davey, J.W., Whibley, A., Nadeau, N.J., Zimin, A.V., Hughes, D.S.T., Ferguson, L.C., Martin, S.H., et al. (2012). Butterfly genome reveals promiscuous exchange of mimicry adaptations among species. Nature **487**:94–98.

Dong, Y., Hu, G., Yu, J., Thu, S.W., Grover, C.E., Zhu, S., and Wendel, J.F. (2020). Salt-tolerance diversity in diploid and polyploid cotton (*Gossypium*) species. Plant J. **101**:1135–1151.

Douglas, G.M., Gos, G., Steige, K.A., Salcedo, A., Holm, K., Josephs, E.B., Arunkumar, R., Ågren, J.A., Hazzouri, K.M., Wang, W., et al. (2015). Hybrid origins and the earliest stages of diploidization in the highly successful recent polyploid *Capsella bursa-pastoris*. Proc. Natl. Acad. Sci. U S A **112**:2806–2811.

Doyle, J.J., and Egan, A.N. (2010). Dating the origins of polyploidy events. New Phytol. **186**:73–85.

Evanno, G., Regnaut, S., and Goudet, J. (2005). Detecting the number of clusters of individuals using the software structure: a simulation study. Mol. Ecol. **14**:2611–2620.

Favre, A., Widmer, A., and Karrenberg, S. (2017). Differential adaptation drives ecological speciation in campions (*Silene*): evidence from a multi-site transplant experiment. New Phytol. **213**:1487–1499.

Feng, S., Ru, D., Sun, Y., Mao, K., Milne, R., and Liu, J. (2019). Trans-lineage polymorphism and nonbifurcating diversification of the genus *Picea*. New Phytol. **222**:576–587.

French, G.C., Hollingsworth, P.M., Silverside, A.J., and Ennos, R.A. (2008). Genetics, taxonomy and the conservation of British *Euphrasia*. Conserv. Genet. **9**:1547–1562.

Green, R.E., Krause, J., Briggs, A.W., Maricic, T., Stenzel, U., Kircher, M., Patterson, N., Li, H., Zhai, W., Fritz, M.H.-Y., et al. (2010). A draft sequence of the Neandertal genome. Science **328**:710–722.

Guggisberg, A., Mansion, G., Kelso, S., and Conti, E. (2006). Evolution of biogeographic patterns, ploidy levels, and breeding systems in a diploid–polyploid species complex of *Primula*. New Phytol. **171**:617–632.

Guignard, M.S., Nichols, R.A., Knell, R.J., Macdonald, A., Romila, C.-A., Trimmer, M., Leitch, I.J., and Leitch, A.R. (2016). Genome size and ploidy influence angiosperm species' biomass under nitrogen and phosphorus limitation. New Phytol. **210**:1195–1206.

Gussarova, G., Alsos, I.G., and Brochmann, C. (2012). Annual plants colonizing the Arctic? Phylogeography and genetic variation in the *Euphrasia minima* complex (Orobanchaceae). Taxon **61**:146–160.

Hollister, J.D., Arnold, B.J., Svedin, E., Xue, K.S., Dilkes, B.P., and Bomblies, K. (2012). Genetic adaptation associated with genome-doubling in autotetraploid Arabidopsis arenosa. PLOS Genet. **8**:e1003093.

Jakobsson, M., and Rosenberg, N.A. (2007). CLUMPP: a cluster matching and permutation program for dealing with label switching and multimodality in analysis of population structure. Bioinformatics **23**:1801–1806.

Jombart, T., and Ahmed, I. (2011). adegenet 1.3-1: new tools for the analysis of genome-wide SNP data. Bioinformatics **27**:3070–3071.

Jørgensen, M.H., Ehrich, D., Schmickl, R., Koch, M.A., and Brysting, A.K. (2011). Interspecific and interploidal gene flow in Central European *Arabidopsis* (Brassicaceae). BMC Evol. Biol. **11**:346.

Kolseth, A.-K., and Lönn, M. (2005). Genetic structure of *Euphrasia stricta* on the Baltic island of Gotland, Sweden. Ecography (Cop.) **28**:443–452.

Köster, J., and Rahmann, S. (2012). Snakemake—a scalable bioinformatics workflow engine. Bioinformatics **28**:2520–2522.

Kryvokhyzha, D., Salcedo, A., Eriksson, M.C., Duan, T., Tawari, N., Chen, J., Guerrina, M., Kreiner, J.M., Kent, T.V., Lagercrantz, U., et al. (2019). Parental legacy, demography, and admixture influenced the evolution of the two subgenomes of the tetraploid *Capsella bursa-pastoris* (Brassicaceae). PLoS Genet. **15**:e1007949.

Laetsch, D.R., and Blaxter, M.L. (2017). BlobTools: Interrogation of genome assemblies. F1000Res. **6**:1287.

Levin, D.A. (2019). Plant speciation in the age of climate change. Ann. Bot. **124**:769–775.

Lohse, K., Harrison, R.J., and Barton, N.H. (2011). A general method for calculating likelihoods under the coalescent process. Genetics **189**:977–987.

Lohse, K., Chmelik, M., Martin, S.H., and Barton, N.H. (2016). Efficient Strategies for calculating blockwise likelihoods under the coalescent. Genetics **202**:775–786.

Lowe, A.J., and Abbott, R.J. (1996). Origins of the new allopolyploid species Senecio cambrensis (Asteraceae) and its relationship to the canary islands endemic Senecio teneriffae. Am. J. Bot. **83**:1365–1372.

Lowe, A.J., and Abbott, R.J. (2015). Hybrid swarms: catalysts for multiple evolutionary events in *Senecio* in the British Isles. Plant Ecol. Divers. **8**:449–463.

Lowry, D.B., Rockwood, R.C., and Willis, J.H. (2008). Ecological reproductive isolation of coast and inland races of *Mimulus guttatus*. Evolution (N. Y). **62**:2196–2214.

Lyu, H., He, Z., Wu, C.-I., and Shi, S. (2018). Convergent adaptive evolution in marginal environments: unloading transposable elements as a common strategy among mangrove genomes. New Phytol. **217**:428–438.

Malinsky, M., Svardal, H., Tyers, A.M., Miska, E.A., Genner, M.J., Turner, G.F., and Durbin, R. (2018). Whole-genome sequences of Malawi cichlids reveal multiple radiations interconnected by gene flow. Nat. Ecol. Evol. **2**:1940–1955.

Mallet, J., Meyer, A., Nosil, P., and Feder, J.L. (2009). Space, sympatry and speciation. J. Evol. Biol. **22**:2332–2341.

Mandáková, T., and Lysak, M.A. (2018). Post-polyploid diploidization and diversification through dysploid changes. Curr. Opin. Plant Biol. **42**:55–65.

Mapleson, D., Garcia Accinelli, G., Kettleborough, G., Wright, J., and Clavijo, B.J. (2016). KAT: a K-mer analysis toolkit to quality control NGS datasets and genome assemblies. Bioinformatics **33**:574–576.

Marques, D.A., Meier, J.I., and Seehausen, O. (2019). A Combinatorial view on speciation and adaptive radiation. Trends Ecol. Evol. **34**:531–544.

Martin, S.H., and van Belleghem, S.M. (2017). Exploring evolutionary relationships across the genome using topology weighting. Genetics **206**:429–438.

McNeilly, T., and Antonovics, J. (1968). Evolution in closely adjacent plant populations IV. Barriers to gene flow. Heredity (Edinb). **23**:205–218.

Metherell, C., and Rumsey, F.J. (2018). In Eyebrights (Euphrasia) of the UK and Ireland, J. Edmondson, ed. (Bristol: Botanical Society of Britain and Ireland).

Morjan, C.L., and Rieseberg, L.H. (2004). How species evolve collectively: implications of gene flow and selection for the spread of advantageous alleles. Mol. Ecol. **13**:1341–1356.

Nadeau, N.J., Martin, S.H., Kozak, K.M., Salazar, C., Dasmahapatra, K.K., Davey, J.W., Baxter, S.W., Blaxter, M.L., Mallet, J., and Jiggins, C.D. (2013). Genome-wide patterns of divergence and gene flow across a butterfly radiation. Mol. Ecol. **22**:814–826.

Nevado, B., Atchison, G.W., Hughes, C.E., and Filatov, D.A. (2016). Widespread adaptive evolution during repeated evolutionary radiations in New World lupins. Nat. Commun. **7**:12384.

Nguyen, L.-T., Schmidt, H.A., von Haeseler, A., and Minh, B.Q. (2014). IQ-TREE: a fast and effective stochastic algorithm for estimating maximum-likelihood phylogenies. Mol. Biol. Evol. **32**:268–274.

Nosil, P., Funk, D.J., and Ortiz-Barrientos, D. (2009). Divergent selection and heterogeneous genomic divergence. Mol. Ecol. **18**:375–402.

Nosil, P., Gompert, Z., Farkas, T.E., Comeault, A.A., Feder, J.L., Buerkle, C.A., and Parchman, T.L. (2012). Genomic consequences of multiple speciation processes in a stick insect. Proc. R. Soc. B Biol. Sci. **279**:5058–5065.

Novák, P., Neumann, P., Pech, J., Steinhaisl, J., and Macas, J. (2013). RepeatExplorer: a Galaxy-based web server for genome-wide characterization of eukaryotic repetitive elements from next-generation sequence reads. Bioinformatics **29**:792–793.

Núñez-Farfán, J., and Schlichting, C.D. (2001). Evolution in changing environments: the "synthetic" work of Clausen, Keck, and Hiesey. Q. Rev. Biol. **76**:433–457.

Papadopulos, A.S.T., Baker, W.J., Crayn, D., Butlin, R.K., Kynast, R.G., Hutton, I., and Savolainen, V. (2011). Speciation with gene flow on Lord Howe island. Proc. Natl. Acad. Sci. U S A **108**:13188–13193.

Paradis, E., and Schliep, K. (2018). ape 5.0: an environment for modern phylogenetics and evolutionary analyses in R. Bioinformatics **35**:526–528.

Pardo-Diaz, C., Salazar, C., Baxter, S.W., Merot, C., Figueiredo-Ready, W., Joron, M., McMillan, W.O., and Jiggins, C.D. (2012). Adaptive introgression across species boundaries in *Heliconius* butterflies. PLoS Genet. **8**:e1002752.

Parisod, C., Holderegger, R., and Brochmann, C. (2010). Evolutionary consequences of autopolyploidy. New Phytol. **186**:5–17.

Peccoud, J., Ollivier, A., Plantegenest, M., and Simon, J.-C. (2009). A continuum of genetic divergence from sympatric host races to species in the pea aphid complex. Proc. Natl. Acad. Sci. U S A **106**:7495–7500.

Popp, M., Erixon, P., Eggens, F., and Oxelman, B. (2005). Origin and evolution of a circumpolar polyploid species complex in Silene (Caryophyllaceae) inferred from low copy nuclear RNA polymerase introns, rDNA, and chloroplast DNA. Syst. Bot. **30**:302–313.

Pritchard, J.K., Stephens, M., and Donnelly, P. (2000). Inference of population structure using multilocus genotype data. Genetics **155**:945–959.

Quinteros Peñafiel, C.V., Riddiford, N.J., and Twyford, A.D. (2017). A floristic survey of Fair Isle. New J. Bot. **7**:101–111.

Raj, A., Stephens, M., and Pritchard, J.K. (2014). fastSTRUCTURE: Variational inference of population structure in large SNP data sets. Genetics **197**:573–589.

Ranallo-Benavidez, T.R., Jaron, K.S., and Schatz, M.C. (2020). GenomeScope 2.0 and Smudgeplot for reference-free profiling of polyploid genomes. Nat. Commun. **11**:1432.

Ravinet, M., Faria, R., Butlin, R.K., Galindo, J., Bierne, N., Rafajlović, M., Noor, M.A.F., Mehlig, B., and Westram, A.M. (2017). Interpreting the genomic landscape of speciation: a road map for finding barriers to gene flow. J. Evol. Biol. **30**:1450–1477.

Ren, R., Wang, H., Guo, C., Zhang, N., Zeng, L., Chen, Y., Ma, H., and Qi, J. (2018). Widespread whole genome duplications contribute to genome complexity and species diversity in Angiosperms. Mol. Plant **11**:414–428.

Renaut, S., Maillet, N., Normandeau, E., Sauvage, C., Derome, N., Rogers, S.M., and Bernatchez, L. (2012). Genome-wide patterns of divergence during speciation: the lake whitefish case study. Philos. Trans. R. Soc. B Biol. Sci. **367**:354–363.

Richards, T.J., Walter, G.M., McGuigan, K., and Ortiz-Barrientos, D. (2016). Divergent natural selection drives the evolution of reproductive isolation in an Australian wildflower. Evolution (N. Y). **70**:1993–2003.

Riddiford, N.J., Quinteros Peñafiel, C.V., Metherell, C., Ferguson-Smyth, C.C., and Twyford, A.D. (2020). A floristic survey of Fair Isle II: new and notable records and the status of Euphrasia. Br. Irish Bot. **2**:144–153.

Rieseberg, L.H., Raymond, O., Rosenthal, D.M., Lai, Z., Livingstone, K., Nakazato, T., Durphy, J.L., Schwarzbach, A.E., Donovan, L.A., and Lexer, C. (2003). Major ecological transitions in wild sunflowers facilitated by hybridization. Science **301**:1211–1216.

Roda, F., Ambrose, L., Walter, G.M., Liu, H.L., Schaul, A., Lowe, A., Pelser, P.B., Prentis, P., Rieseberg, L.H., and Ortiz-Barrientos, D. (2013). Genomic evidence for the parallel evolution of coastal forms in the Senecio lautus complex. Mol. Ecol. **22**:2941–2952.

Schwarzbach, A.E., and Rieseberg, L.H. (2002). Likely multiple origins of a diploid hybrid sunflower species. Mol. Ecol. **11**:1703–1715.

Soltis, D.E., Soltis, P.S., Pires, J.C., Kovarik, A., Tate, J.A., and Mavrodiev, E. (2004). Recent and recurrent polyploidy in *Tragopogon* (Asteraceae): cytogenetic, genomic and genetic comparisons. Biol. J. Linn. Soc. **82**:485–501.

Soltis, D.E., Buggs, R.J.A., Barbazuk, W.B., Chamala, S., Chester, M., Gallagher, J.P., Schnable, P.S., and Soltis, P.S. (2012). The early stages of polyploidy: rapid and repeated evolution in *Tragopogon*. In Polyploidy and Genome Evolution, P.S. Soltis and D.E. Soltis, eds. (Berlin, Heidelberg: Springer Berlin Heidelberg), pp. 271–292.

Squirrell, J., Hollingsworth, P.M., Bateman, R.M., Tebbitt, M.C., and Hollingsworth, M.L. (2002). Taxonomic complexity and breeding system transitions: conservation genetics of the *Epipactis leptochila* complex (Orchidaceae). Mol. Ecol. **11**:1957–1964.

Stace, C. (2019). New Flora of the British Isles, 4th edn (Middlewood Green, Suffolk: C & M Floristics).

Stebbins, G.L. (1984). Polyploidy and the distribution of the arctic-alpine flora: new evidence and a new approach. Bot. Helv. **94**:1–13.

Stone, H. (2013). Evolution and conservation of tetraploid Euphrasia L. in Britain, http://hdl.handle.net/1842/7740.

Sun, Y., Abbott, R.J., Lu, Z., Mao, K., Zhang, L., Wang, X., Ru, D., and Liu, J. (2018). Reticulate evolution within a spruce (*Picea*) species complex revealed by population genomic analysis. Evolution (N. Y). **72**:2669–2681.

Tank, D.C., Eastman, J.M., Pennell, M.W., Soltis, P.S., Soltis, D.E., Hinchliff, C.E., Brown, J.W., Sessa, E.B., and Harmon, L.J. (2015). Nested radiations and the pulse of angiosperm diversification: increased diversification rates often follow whole genome duplications. New Phytol. **207**:454–467.

Twyford, A.D., Kidner, C.A., and Ennos, R.A. (2014). Genetic differentiation and species cohesion in two widespread Central American *Begonia* species. Heredity (Edinb) **112**:382–390.

Twyford, A.D., Kidner, C.A., and Ennos, R.A. (2015). Maintenance of species boundaries in a Neotropical radiation of Begonia. Mol. Ecol. **24**:4982–4993.

VanBuren, R., Man Wai, C., Wang, X., Pardo, J., Yocca, A.E., Wang, H., Chaluvadi, S.R., Han, G., Bryant, D., Edger, P.P., et al. (2020). Exceptional subgenome stability and functional divergence in the allotetraploid Ethiopian cereal teff. Nat. Commun. **11**:884.

Via, S. (2009). Natural selection in action during speciation. Proc. Natl. Acad. Sci. U S A **106**:9939–9946.

Voichek, Y., and Weigel, D. (2020). Identifying genetic variants underlying phenotypic variation in plants without complete genomes. Nat. Genet. **52**:534–540.

Vurture, G.W., Sedlazeck, F.J., Nattestad, M., Underwood, C.J., Fang, H., Gurtowski, J., and Schatz, M.C. (2017). GenomeScope: fast reference-free genome profiling from short reads. Bioinformatics **33**:2202–2204.

Wang, X., Gussarova, G., Ruhsam, M., de Vere, N., Metherell, C., Hollingsworth, P.M., and Twyford, A.D. (2018). DNA barcoding a taxonomically complex hemiparasitic genus reveals deep divergence between ploidy levels but lack of species-level resolution. AoB Plants **10**:ply026.

Warren, R.L., Coombe, L., Mohamadi, H., Zhang, J., Jaquish, B., Isabel, N., Jones, S.J.M., Bousquet, J., Bohlmann, J., and Birol, I. (2019). ntEdit: scalable genome sequence polishing. Bioinformatics **35**:4430–4432.

Wendel, J.F., Jackson, S.A., Meyers, B.C., and Wing, R.A. (2016). Evolution of plant genome architecture. Genome Biol. **17**:37.

Wood, T.E., Takebayashi, N., Barker, M.S., Mayrose, I., Greenspoon, P.B., and Rieseberg, L.H. (2009). The frequency of polyploid speciation in vascular plants. Proc. Natl. Acad. Sci. **106**:13875–13879.

Wyman, S.K., Jansen, R.K., and Boore, J.L. (2004). Automatic annotation of organellar genomes with DOGMA. Bioinformatics **20**:3252–3255.

Yeo, P. (1956). Hybridization between diploid and tetraploid species of *Euphrasia*. Watsonia **3**:253–269.

Yeo, P. (1966). The breeding relationships of some European *Euphrasiae*. Watsonia **6**:216–245.

Zhang, C., Rabiee, M., Sayyari, E., and Mirarab, S. (2018). ASTRAL-III: polynomial time species tree reconstruction from partially resolved gene trees. BMC Bioinformatics **19**:153.

Plant Communications, Volume *1*

Supplemental Information

Maintenance of Species Differences in Closely Related Tetraploid Parasitic *Euphrasia* (Orobanchaceae) on an Isolated Island

Hannes Becher, Max R. Brown, Gavin Powell, Chris Metherell, Nick J. Riddiford, and Alex D. Twyford

Supplemental Items to Becher et al. 2020 “Maintenance of species differences in closely related tetraploid parasitic *Euphrasia* (Orobanchaceae) on an isolated island”

By Hannes Becher, Max R. Brown, Gavin Powell, Chris Metherell, Nick J. Riddiford, and Alex D. Twyford

Contents

Supplemental figure 1 II
Supplemental figure 2 III
Supplemental table 1 IV
Supplemental table 2 IV
Supplemental table 3 IV
Supplemental table 4 IV
Supplemental text 1 – Magnitude and significance of trait value differences V
Order of traits V
Trait differences in natural populations, species level V
Trait differences in natural populations, population level VII
Trait differences in the common garden, species level X
Trait differences in the common garden, population level XIII
Supplemental text 2 – Models for k-mer spectra XVIII
Usage example – autotetraploid XIX
Usage example – allotetraploid XX
Supplemental text 3 – Genome profiling XXII
Euphrasia anglica (AN, diploid) XXIII
Euphrasia vigursii (VI, diploid) XXIV
Euphrasia rivularis (RI, diploid) XXV
Euphrasia rostkoviana (RO, diploid) XXVI
Euphrasia arctica (A0, tetraploid) XXVII
Euphrasia arctica (A1, tetraploid) XXVIII
Euphrasia arctica (A2, tetraploid) XXIX
Euphrasia arctica (A3, tetraploid) XXX
Euphrasia foulaensis (F1, tetraploid) XXXI

Euphrasia foulaensis (F2, tetraploid) XXXII
Euphrasia foulaensis (F3, tetraploid) XXXIII
Euphrasia foulaensis (F4, tetraploid) XXXIV
Euphrasia micrantha (M0, tetraploid) XXXV
Euphrasia micrantha (M1, tetraploid) XXXVI
Euphrasia micrantha (M2, tetraploid) XXXVII
Euphrasia micrantha (M3, tetraploid) XXXVIII
Euphrasia sp. (X1, tetraploid) XXXIX
Euphrasia sp. (X2, tetraploid) XL

SUPPLEMENTAL FIGURE 1

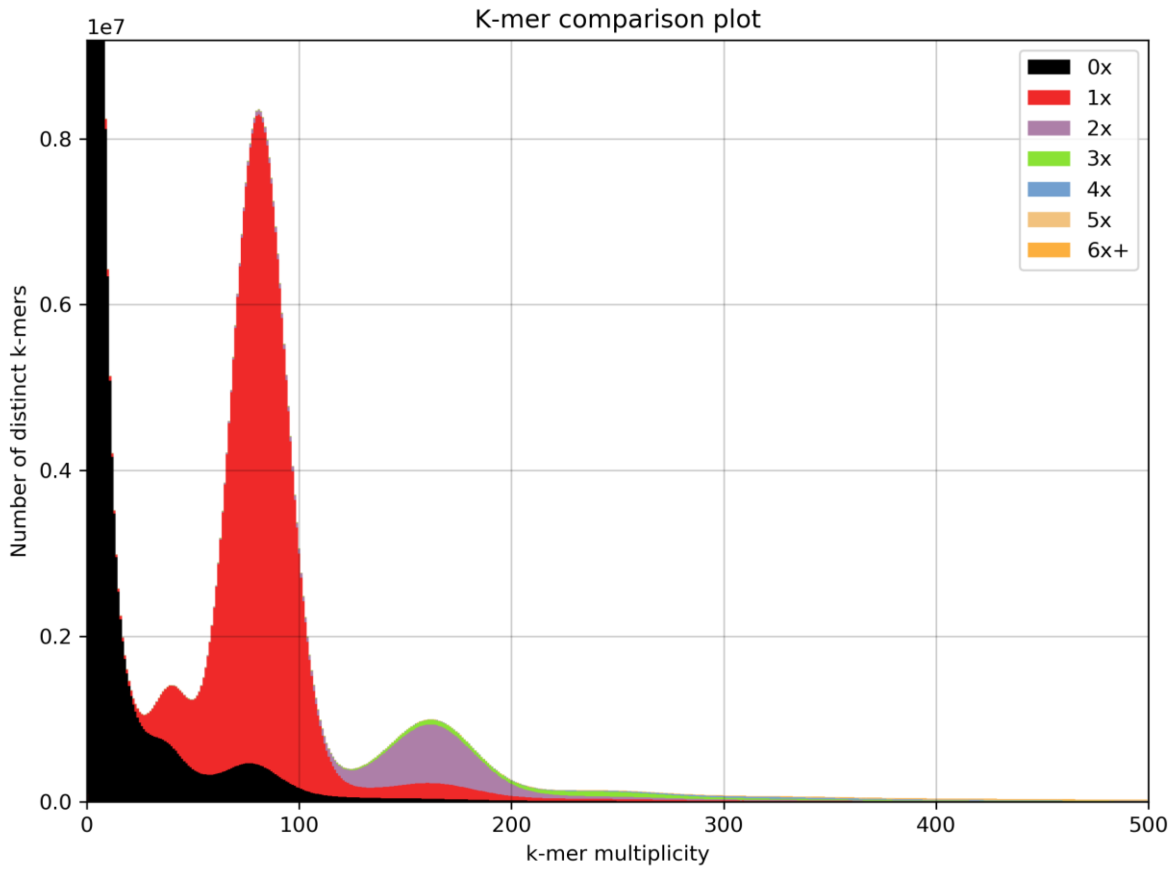

A k-mer completeness plot for our assembly of *E. arctica* generated with KAT.

SUPPLEMENTAL FIGURE 2

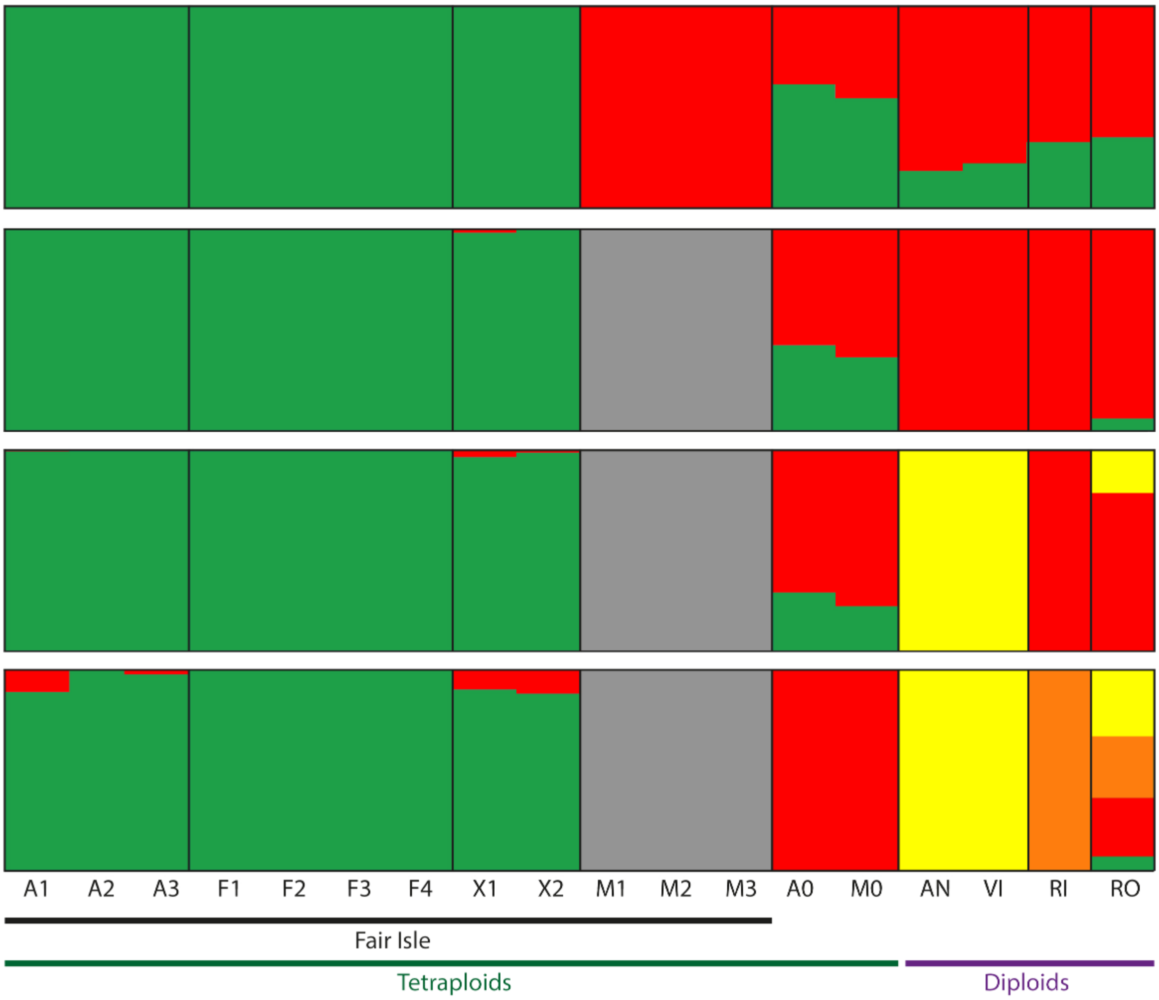

Output of STRUCTURE runs with K values 2-5.

SUPPLEMENTAL TABLE 1

Locations and descriptions of populations sampled on Fair Isle.

	Population code	latitude	longitude	Description
E. arctica	G1	59.524622	-1.636012	Road side, near school
E. arctica	G2	59.517135	-1.64082	Road side, near chapel
E. foulaensis	C1	59.535645	-1.602904	Coastal turf, Buness peninsula
E. foulaensis	C2	59.513855	-1.650254	Coastal turf, South light
E. micrantha	H1	59.537202	-1.629517	Heath land, North of airstrip
E. micrantha	H2	59.548057	-1.615796	Heath land, by bend in road to N light

SUPPLEMENTAL TABLE 2

Means and standard errors of trait measurements from natural populations (Fair Isle) and from the common garden.

Submitted separately.

SUPPLEMENTAL TABLE 3

Origins of host seed used in the common garden experiment.

Species	Source	Notes
Holcus lanatus	Emorsgate Seeds*	Seeds ordered in early 2019
Lolium perenne	Emorsgate Seeds*	Seeds ordered in early 2019
Trifolium repens	Emorsgate Seeds*	Seeds ordered in early 2019
Armeria maritima	Scotia Seeds**	Seeds ordered in early 2019
Juniperus vulgaris	Fair Isle, wild collected	cuttings taken in September 2018
Calluna vulgaris	Fair Isle, wild collected	cuttings taken in September 2018
Plantago maritima	Fair Isle, wild collected	seeds collected in August 2018
Plantago lanceolata	Fair Isle, wild collected	seeds collected in August 2018
Plantago coronopus	Fair Isle, wild collected	seeds collected in August 2018
Rumex crispus	Fair Isle, wild collected	seeds collected in August 2018
Luzula multiflora	Fair Isle, wild collected	seeds collected in August 2018
Rumex acetosa	Fair Isle, wild collected	seeds collected in August 2018

* EMORSGATE SEEDS, Limes Farm, Tilney All Saints, King's Lynn, Norfolk, PE34 4RT	** Scotia Seeds, Mavisbank, Farnell, Brechin, Angus, DD9 6TR	

SUPPLEMENTAL TABLE 4

Individuals sequenced, heterozygosity, and sub-genome divergence estimates.

Submitted separately.

SUPPLEMENTAL TEXT 1 – MAGNITUDE AND SIGNIFICANCE OF TRAIT VALUE DIFFERENCES

ORDER OF TRAITS

The order of traits in the following tables corresponds to Figure 1D. Traits marked with * were recorded from natural populations.

1. Time transplant to flower in days
2. Time of 1st flower in Julian days
3. Height at 1st flower in mm
4. *Final height in mm
5. Number of reproductive nodes
6. *Corolla length in mm
7. *Number of nodes blow the flower
8. *Ration of the length of the leaf subtending the lowest flower and the internode beneath
9. *Number of leaf teeth
10. *Capsule width in mm
11. *Capsule length in mm
12. Germination rate
13. Proportion flowering of those germinated
14. Proportion early death out of those germinated

TRAIT DIFFERENCES IN NATURAL POPULATIONS, SPECIES LEVEL

```
4
        Simultaneous Tests for General Linear Hypotheses

Fit: lmer(formula = log(height) ~ Category + (1 | Population), data = datcomp)

Linear Hypotheses:
                       Estimate Std. Error t value Pr(>|t|)
arc - fou == 0  1.39687    0.08663  16.125  0.00127 **
arc - mic == 0  0.71272    0.08663   8.227  0.00779 **
fou - mic == 0 -0.68415    0.08663  -7.897  0.00846 **
---
Signif. codes:  0 '*' 0.001 '' 0.01 '*' 0.05 '.' 0.1 ' ' 1
(Adjusted p values reported -- single-step method)
```

```
6

        Simultaneous Tests for General Linear Hypotheses

Fit: lmer(formula = corolla.size ~ Category + (1 | Population), data = datcomp)

Linear Hypotheses:
                       Estimate Std. Error t value Pr(>|t|)
arc - fou == 0   1.7196     0.1312  13.105  0.00193 **
arc - mic == 0   2.9225     0.1483  19.706  < 0.001 ***
fou - mic == 0   1.2029     0.1495   8.045  0.00807 **
---
Signif. codes:  0 '*' 0.001 '' 0.01 '*' 0.05 '.' 0.1 ' ' 1
(Adjusted p values reported -- single-step method)
```

7

```
        Simultaneous Tests for General Linear Hypotheses

Fit: lmer(formula = nd.to.fl ~ Category + (1 | Population), data =
datcomp[!rownames(datcomp) %in%
       c("17", "113"), ])

Linear Hypotheses:
                       Estimate Std. Error t value Pr(>|t|)
arc - fou == 0   1.5556     0.5485   2.836   0.1280
arc - mic == 0   2.4028     0.5491   4.376   0.0442 *
fou - mic == 0   0.8472     0.5480   1.546   0.3897
---
Signif. codes:  0 ‘*’ 0.001 ‘’ 0.01 ‘*’ 0.05 ‘.’ 0.1 ‘ ’ 1
(Adjusted p values reported -- single-step method)
```

8

```
        Simultaneous Tests for General Linear Hypotheses

Fit: lmer(formula = log(lir) ~ Category + (1 | Population), data = datcomp)

Linear Hypotheses:
                       Estimate Std. Error t value Pr(>|t|)
arc - fou == 0  -0.7981     0.2801  -2.850   0.1266
arc - mic == 0   0.7305     0.2803   2.606   0.1540
fou - mic == 0   1.5286     0.2798   5.464   0.0244 *
---
Signif. codes:  0 ‘*’ 0.001 ‘’ 0.01 ‘*’ 0.05 ‘.’ 0.1 ‘ ’ 1
(Adjusted p values reported -- single-step method)
```

9

```
        Simultaneous Tests for General Linear Hypotheses

Fit: lmer(formula = leaf.teeth ~ Category + (1 | Population), data = datcomp)

Linear Hypotheses:
                       Estimate Std. Error t value Pr(>|t|)
arc - fou == 0   1.1742     0.5699   2.060    0.245
arc - mic == 0   1.5244     0.5702   2.674    0.146
fou - mic == 0   0.3502     0.5697   0.615    0.823
(Adjusted p values reported -- single-step method)
```

10

```
        Simultaneous Tests for General Linear Hypotheses

Fit: lmer(formula = capsule.width ~ Category + (1 | Population), data =
datcomp[!rownames(datcomp) %in%
       c("17", "113"), ])

Linear Hypotheses:
                       Estimate Std. Error t value Pr(>|t|)
arc - fou == 0  -0.1279     0.3423  -0.374    0.928
arc - mic == 0   0.5693     0.3410   1.669    0.348
fou - mic == 0   0.6972     0.3419   2.039    0.250
(Adjusted p values reported -- single-step method)
```

11

```
        Simultaneous Tests for General Linear Hypotheses
```

```
Fit: lmer(formula = capsule.width ~ Category + (1 | Population), data =
datcomp[!rownames(datcomp) %in%
       c("17", "113"), ])

Linear Hypotheses:
                       Estimate Std. Error t value Pr(>|t|)
arc - fou == 0  -0.1279     0.3423  -0.374    0.928
arc - mic == 0   0.5693     0.3410   1.669    0.348
fou - mic == 0   0.6972     0.3419   2.039    0.250
(Adjusted p values reported -- single-step method)
```

TRAIT DIFFERENCES IN NATURAL POPULATIONS, POPULATION LEVEL

4

```
        Simultaneous Tests for General Linear Hypotheses

Fit: lm(formula = log(height) ~ Population, data = datcomp)

Linear Hypotheses:
                     Estimate Std. Error t value Pr(>|t|)
G1 - G2 == 0 -0.04677    0.07041  -0.664   0.9856
G1 - C2 == 0  1.41311    0.07041  20.070   <0.001 ***
G1 - C1 == 0  1.33386    0.07041  18.944   <0.001 ***
G1 - H2 == 0  0.78494    0.07041  11.148   <0.001 ***
G1 - H1 == 0  0.59373    0.07041   8.432   <0.001 ***
G2 - C2 == 0  1.45988    0.07041  20.734   <0.001 ***
G2 - C1 == 0  1.38063    0.07041  19.608   <0.001 ***
G2 - H2 == 0  0.83171    0.07041  11.812   <0.001 ***
G2 - H1 == 0  0.64050    0.07041   9.097   <0.001 ***
C2 - C1 == 0 -0.07925    0.07041  -1.126   0.8702
C2 - H2 == 0 -0.62817    0.07041  -8.922   <0.001 ***
C2 - H1 == 0 -0.81938    0.07041 -11.637   <0.001 ***
C1 - H2 == 0 -0.54892    0.07041  -7.796   <0.001 ***
C1 - H1 == 0 -0.74013    0.07041 -10.512   <0.001 ***
H2 - H1 == 0 -0.19121    0.07041  -2.716   0.0772 .
---
Signif. codes:  0 '*' 0.001 '' 0.01 '*' 0.05 '.' 0.1 ' ' 1
(Adjusted p values reported -- single-step method)
```

6

```
        Simultaneous Tests for General Linear Hypotheses

Fit: lm(formula = corolla.size ~ Population, data = datcomp)

Linear Hypotheses:
                     Estimate Std. Error t value Pr(>|t|)
G1 - G2 == 0  0.08214    0.15825   0.519    0.995
G1 - C2 == 0  1.61671    0.16293   9.923   <0.001 ***
G1 - C1 == 0  1.89286    0.15825  11.961   <0.001 ***
G1 - H2 == 0  3.06071    0.20430  14.981   <0.001 ***
G1 - H1 == 0  2.89756    0.17600  16.464   <0.001 ***
G2 - C2 == 0  1.53457    0.16293   9.419   <0.001 ***
G2 - C1 == 0  1.81071    0.15825  11.442   <0.001 ***
G2 - H2 == 0  2.97857    0.20430  14.579   <0.001 ***
G2 - H1 == 0  2.81541    0.17600  15.997   <0.001 ***
```

```
C2 - C1 == 0  0.27614    0.16293   1.695    0.533
C2 - H2 == 0  1.44400    0.20794   6.944   <0.001 ***
C2 - H1 == 0  1.28084    0.18021   7.107   <0.001 ***
C1 - H2 == 0  1.16786    0.20430   5.716   <0.001 ***
C1 - H1 == 0  1.00470    0.17600   5.709   <0.001 ***
H2 - H1 == 0 -0.16316    0.21833  -0.747    0.975
---
Signif. codes:  0 ‘*’ 0.001 ‘’ 0.01 ‘*’ 0.05 ‘.’ 0.1 ‘ ’ 1
(Adjusted p values reported -- single-step method)
```

7

```
         Simultaneous Tests for General Linear Hypotheses

Fit: lm(formula = nd.to.fl ~ Population, data = datcomp[!rownames(datcomp) %in%
       c("17", "113"), ])

Linear Hypotheses:
                     Estimate Std. Error t value Pr(>|t|)
G1 - G2 == 0   1.2881     0.3719   3.463  0.00864 **
G1 - C2 == 0   2.3548     0.3719   6.331  < 0.001 ***
G1 - C1 == 0   2.0548     0.3719   5.525  < 0.001 ***
G1 - H2 == 0   2.9214     0.3719   7.855  < 0.001 ***
G1 - H1 == 0   3.1835     0.3750   8.489  < 0.001 ***
G2 - C2 == 0   1.0667     0.3654   2.919  0.04525 *
G2 - C1 == 0   0.7667     0.3654   2.098  0.29328
G2 - H2 == 0   1.6333     0.3654   4.469  < 0.001 ***
G2 - H1 == 0   1.8954     0.3686   5.142  < 0.001 ***
C2 - C1 == 0  -0.3000     0.3654  -0.821  0.96334
C2 - H2 == 0   0.5667     0.3654   1.551  0.63200
C2 - H1 == 0   0.8287     0.3686   2.248  0.22144
C1 - H2 == 0   0.8667     0.3654   2.372  0.17224
C1 - H1 == 0   1.1287     0.3686   3.062  0.03012 *
H2 - H1 == 0   0.2621     0.3686   0.711  0.98041
---
Signif. codes:  0 ‘*’ 0.001 ‘’ 0.01 ‘*’ 0.05 ‘.’ 0.1 ‘ ’ 1
(Adjusted p values reported -- single-step method)
```

8

```
         Simultaneous Tests for General Linear Hypotheses

Fit: lm(formula = log(lir) ~ Population, data = datcomp)

Linear Hypotheses:
                     Estimate Std. Error t value Pr(>|t|)
G1 - G2 == 0   0.3861     0.1105   3.493  0.00797 **
G1 - C2 == 0  -0.8379     0.1068  -7.846  < 0.001 ***
G1 - C1 == 0  -0.3711     0.1085  -3.419  0.01012 *
G1 - H2 == 0   0.7628     0.1095   6.965  < 0.001 ***
G1 - H1 == 0   1.0839     0.1085   9.985  < 0.001 ***
G2 - C2 == 0  -1.2240     0.1068 -11.462  < 0.001 ***
G2 - C1 == 0  -0.7572     0.1085  -6.976  < 0.001 ***
G2 - H2 == 0   0.3767     0.1095   3.440  0.00949 **
G2 - H1 == 0   0.6978     0.1085   6.428  < 0.001 ***
C2 - C1 == 0   0.4668     0.1047   4.457  < 0.001 ***
C2 - H2 == 0   1.6007     0.1057  15.140  < 0.001 ***
C2 - H1 == 0   1.9218     0.1047  18.350  < 0.001 ***
C1 - H2 == 0   1.1339     0.1075  10.548  < 0.001 ***
C1 - H1 == 0   1.4550     0.1065  13.660  < 0.001 ***
H2 - H1 == 0   0.3211     0.1075   2.987  0.03774 *
---
Signif. codes:  0 ‘*’ 0.001 ‘’ 0.01 ‘*’ 0.05 ‘.’ 0.1 ‘ ’ 1
(Adjusted p values reported -- single-step method)
```

9

```
	 Simultaneous Tests for General Linear Hypotheses

Fit: lm(formula = leaf.teeth ~ Population, data = datcomp)

Linear Hypotheses:
                    Estimate Std. Error t value Pr(>|t|)    
G1 - G2 == 0  0.85755    0.15126   5.669   <0.001 ***
G1 - C2 == 0  1.05385    0.14750   7.145   <0.001 ***
G1 - C1 == 0  2.15385    0.14993  14.366   <0.001 ***
G1 - H2 == 0  1.93162    0.15126  12.770   <0.001 ***
G1 - H1 == 0  1.97527    0.14993  13.175   <0.001 ***
G2 - C2 == 0  0.19630    0.14603   1.344    0.760    
G2 - C1 == 0  1.29630    0.14848   8.730   <0.001 ***
G2 - H2 == 0  1.07407    0.14983   7.169   <0.001 ***
G2 - H1 == 0  1.11772    0.14848   7.528   <0.001 ***
C2 - C1 == 0  1.10000    0.14465   7.604   <0.001 ***
C2 - H2 == 0  0.87778    0.14603   6.011   <0.001 ***
C2 - H1 == 0  0.92143    0.14465   6.370   <0.001 ***
C1 - H2 == 0 -0.22222    0.14848  -1.497    0.667    
C1 - H1 == 0 -0.17857    0.14713  -1.214    0.830    
H2 - H1 == 0  0.04365    0.14848   0.294    1.000    
---
Signif. codes:  0 '*' 0.001 '' 0.01 '*' 0.05 '.' 0.1 ' ' 1
(Adjusted p values reported -- single-step method)
```

10

```
	 Simultaneous Tests for General Linear Hypotheses

Fit: lm(formula = capsule.width ~ Population, data = datcomp[!rownames(datcomp) 
%in% 
    c("17", "113"), ])

Linear Hypotheses:
                     Estimate Std. Error t value Pr(>|t|)    
G1 - G2 == 0 -0.706981   0.094030  -7.519   <0.001 ***
G1 - C2 == 0 -0.537500   0.095276  -5.642   <0.001 ***
G1 - C1 == 0 -0.421627   0.099709  -4.229   <0.001 ***
G1 - H2 == 0  0.003929   0.086725   0.045   1.0000    
G1 - H1 == 0  0.432005   0.089888   4.806   <0.001 ***
G2 - C2 == 0  0.169481   0.100690   1.683   0.5438    
G2 - C1 == 0  0.285354   0.104895   2.720   0.0771 .  
G2 - H2 == 0  0.710909   0.092641   7.674   <0.001 ***
G2 - H1 == 0  1.138986   0.095608  11.913   <0.001 ***
C2 - C1 == 0  0.115873   0.106013   1.093   0.8828    
C2 - H2 == 0  0.541429   0.093905   5.766   <0.001 ***
C2 - H1 == 0  0.969505   0.096833  10.012   <0.001 ***
C1 - H2 == 0  0.425556   0.098400   4.325   <0.001 ***
C1 - H1 == 0  0.853632   0.101199   8.435   <0.001 ***
H2 - H1 == 0  0.428077   0.088434   4.841   <0.001 ***
---
Signif. codes:  0 '*' 0.001 '' 0.01 '*' 0.05 '.' 0.1 ' ' 1
(Adjusted p values reported -- single-step method)
```

11

```
	 Simultaneous Tests for General Linear Hypotheses

Fit: lm(formula = capsule.width ~ Population, data = datcomp[!rownames(datcomp) 
%in% 
    c("17", "113"), ])

Linear Hypotheses:
                     Estimate Std. Error t value Pr(>|t|)
```

```
G1 - G2 == 0 -0.706981   0.094030  -7.519   <0.001 ***
G1 - C2 == 0 -0.537500   0.095276  -5.642   <0.001 ***
G1 - C1 == 0 -0.421627   0.099709  -4.229   <0.001 ***
G1 - H2 == 0  0.003929   0.086725   0.045   1.0000
G1 - H1 == 0  0.432005   0.089888   4.806   <0.001 ***
G2 - C2 == 0  0.169481   0.100690   1.683   0.5438
G2 - C1 == 0  0.285354   0.104895   2.720   0.0771 .
G2 - H2 == 0  0.710909   0.092641   7.674   <0.001 ***
G2 - H1 == 0  1.138986   0.095608  11.913   <0.001 ***
C2 - C1 == 0  0.115873   0.106013   1.093   0.8829
C2 - H2 == 0  0.541429   0.093905   5.766   <0.001 ***
C2 - H1 == 0  0.969505   0.096833  10.012   <0.001 ***
C1 - H2 == 0  0.425556   0.098400   4.325   <0.001 ***
C1 - H1 == 0  0.853632   0.101199   8.435   <0.001 ***
H2 - H1 == 0  0.428077   0.088434   4.841   <0.001 ***
---
Signif. codes:  0 ‘*’ 0.001 ‘’ 0.01 ‘*’ 0.05 ‘.’ 0.1 ‘ ’ 1
(Adjusted p values reported -- single-step method)
```

TRAIT DIFFERENCES IN THE COMMON GARDEN, SPECIES LEVEL

1

```
        Simultaneous Tests for General Linear Hypotheses

Fit: lmer(formula = Fd ~ Euphrasia.Taxon + (1 | gerGT) + (1 | Host),
       data = FdAll, REML = T)

Linear Hypotheses:
                       Estimate Std. Error t value Pr(>|t|)
arc - fou == 0    1.578      5.537   0.285    0.957
arc - mic == 0   -4.969      5.684  -0.874    0.690
fou - mic == 0   -6.547      5.716  -1.145    0.555
(Adjusted p values reported -- single-step method)
```

2

```
        Simultaneous Tests for General Linear Hypotheses

Fit: lmer(formula = Flower ~ Euphrasia.Taxon + (1 | gerGT) + (1 |
       Host), data = FdAll, REML = T)

Linear Hypotheses:
                       Estimate Std. Error t value Pr(>|t|)
arc - fou == 0  -0.8433     3.5089  -0.240    0.969
arc - mic == 0  -2.5972     3.6740  -0.707    0.777
fou - mic == 0  -1.7539     3.7106  -0.473    0.888
(Adjusted p values reported -- single-step method)
```

3

```
        Simultaneous Tests for General Linear Hypotheses

Fit: lmer(formula = log(Plant.height) ~ Euphrasia.Taxon + Transplant +
       (1 | gerGT) + (1 | Host), data = FdAll, REML = T)

Linear Hypotheses:
                       Estimate Std. Error t value Pr(>|t|)
arc - fou == 0   0.1487     0.1744   0.853    0.701
arc - mic == 0   0.2550     0.1782   1.431    0.432
fou - mic == 0   0.1062     0.1790   0.593    0.833
(Adjusted p values reported -- single-step method)
```

```
4

        Simultaneous Tests for General Linear Hypotheses

Fit: lmer(formula = log(Height) ~ Euphrasia.Taxon + Transplant + (1 |
       gerGT) + (1 | Host), data = FdAll, REML = T)

Linear Hypotheses:
                      Estimate Std. Error t value Pr(>|t|)
arc - fou == 0  0.25164    0.19112   1.317    0.478
arc - mic == 0  0.34798    0.19790   1.758    0.321
fou - mic == 0  0.09633    0.19926   0.483    0.884
(Adjusted p values reported -- single-step method)


5

        Simultaneous Tests for General Linear Hypotheses

Fit: lmer(formula = log(1 + Reproductive.nodes) ~ Euphrasia.Taxon +
       Transplant + (1 | gerGT) + (1 | Host), data = FdAll, REML = T)

Linear Hypotheses:
                      Estimate Std. Error t value Pr(>|t|)
arc - fou == 0 -0.09066    0.15157  -0.598    0.831
arc - mic == 0  0.41922    0.16520   2.538    0.163
fou - mic == 0  0.50987    0.16741   3.046    0.109
(Adjusted p values reported -- single-step method)


6

        Simultaneous Tests for General Linear Hypotheses

Fit: lmer(formula = Corolla.length ~ Euphrasia.Taxon + (1 | gerGT) +
       (1 | Host), data = FdAll, REML = T)

Linear Hypotheses:
                      Estimate Std. Error t value Pr(>|t|)
arc - fou == 0   0.4772     0.3837   1.244   0.5100
arc - mic == 0   1.7766     0.3879   4.580   0.0393 *
fou - mic == 0   1.2994     0.3888   3.342   0.0874 .
---
Signif. codes:  0 '*' 0.001 '' 0.01 '*' 0.05 '.' 0.1 ' ' 1
(Adjusted p values reported -- single-step method)


7

        Simultaneous Tests for General Linear Hypotheses

Fit: lmer(formula = Nodes.to.flower ~ Euphrasia.Taxon + (1 | gerGT) +
       (1 | Host), data = FdAll, REML = T)

Linear Hypotheses:
                      Estimate Std. Error t value Pr(>|t|)
arc - fou == 0  -0.5544     0.6536  -0.848    0.704
arc - mic == 0   0.5287     0.6601   0.801    0.728
fou - mic == 0   1.0831     0.6617   1.637    0.359
(Adjusted p values reported -- single-step method)


8

        Simultaneous Tests for General Linear Hypotheses

Fit: lmer(formula = log(lir) ~ Euphrasia.Taxon + (1 | gerGT) + (1 |
```

```
       Host), data = FdAll, REML = T)

Linear Hypotheses:
                       Estimate Std. Error t value Pr(>|t|)
arc - fou == 0  -0.3040     0.3557  -0.855    0.700
arc - mic == 0   0.4369     0.3586   1.218    0.521
fou - mic == 0   0.7410     0.3592   2.063    0.245
(Adjusted p values reported -- single-step method)
```

9

```
        Simultaneous Tests for General Linear Hypotheses

Fit: lmer(formula = log(Number.of.leaf.teeth..exc..tip.) ~ Euphrasia.Taxon +
       Transplant + (1 | gerGT) + (1 | Host), data = FdAll)

Linear Hypotheses:
                       Estimate Std. Error t value Pr(>|t|)
arc - fou == 0 -0.02808    0.23120  -0.121    0.992
arc - mic == 0  0.12424    0.23278   0.534    0.861
fou - mic == 0  0.15232    0.23312   0.653    0.804
(Adjusted p values reported -- single-step method)
```

10

```
        Simultaneous Tests for General Linear Hypotheses

Fit: lmer(formula = Capsule.width ~ Euphrasia.Taxon + (1 | gerGT) +
       (1 | Host), data = FdAll, REML = T)

Linear Hypotheses:
                       Estimate Std. Error t value Pr(>|t|)
arc - fou == 0   0.1145     0.1379   0.831   0.7127
arc - mic == 0   0.7230     0.1474   4.904   0.0328 *
fou - mic == 0   0.6084     0.1479   4.113   0.0521 .
---
Signif. codes:  0 '*' 0.001 '' 0.01 '*' 0.05 '.' 0.1 ' ' 1
(Adjusted p values reported -- single-step method)
```

11

```
        Simultaneous Tests for General Linear Hypotheses

Fit: lmer(formula = Capsule.Height ~ Euphrasia.Taxon + (1 | gerGT) +
       (1 | Host), data = FdAll, REML = T)

Linear Hypotheses:
                       Estimate Std. Error t value Pr(>|t|)
arc - fou == 0   0.7293     0.1311   5.562  0.00601 **
arc - mic == 0   1.6583     0.1766   9.388  < 0.001 ***
fou - mic == 0   0.9289     0.1789   5.193  0.00816 **
---
Signif. codes:  0 '*' 0.001 '' 0.01 '*' 0.05 '.' 0.1 ' ' 1
(Adjusted p values reported -- single-step method)
```

12

```
        Simultaneous Tests for General Linear Hypotheses

Fit: glmer(formula = cbind(X1, X2) ~ spec + (1 | GT), data = ger,
       family = binomial)

Linear Hypotheses:
                       Estimate Std. Error z value Pr(>|z|)
```

```
arc - fou == 0   0.6520     0.6027   1.082    0.525
arc - mic == 0   1.5402     0.6041   2.550    0.029 *
fou - mic == 0   0.8882     0.6039   1.471    0.305
---
Signif. codes:  0 ‘*’ 0.001 ‘’ 0.01 ‘*’ 0.05 ‘.’ 0.1 ‘ ’ 1
(Adjusted p values reported -- single-step method)
```

13

```
        Simultaneous Tests for General Linear Hypotheses

Fit: glmer(formula = cbind(X1, X2) ~ spec + (1 | GT), data = flo,
       family = binomial)

Linear Hypotheses:
                      Estimate Std. Error z value Pr(>|z|)
arc - fou == 0  -0.2370     0.1278  -1.854  0.15111
arc - mic == 0  -0.5316     0.1534  -3.465  0.00147 **
fou - mic == 0  -0.2946     0.1573  -1.873  0.14514
---
Signif. codes:  0 ‘*’ 0.001 ‘’ 0.01 ‘*’ 0.05 ‘.’ 0.1 ‘ ’ 1
(Adjusted p values reported -- single-step method)
```

14

```
        Simultaneous Tests for General Linear Hypotheses

Fit: glmer(formula = cbind(X1, X2) ~ spec + (1 | GT), data = dead,
       family = binomial)

Linear Hypotheses:
                      Estimate Std. Error z value Pr(>|z|)
arc - fou == 0  -0.1995     0.1915  -1.042  0.54653
arc - mic == 0   0.5756     0.2477   2.323  0.05156 .
fou - mic == 0   0.7751     0.2617   2.962  0.00811 **
---
Signif. codes:  0 ‘*’ 0.001 ‘’ 0.01 ‘*’ 0.05 ‘.’ 0.1 ‘ ’ 1
(Adjusted p values reported -- single-step method)
```

TRAIT DIFFERENCES IN THE COMMON GARDEN, POPULATION LEVEL

1

```
        Simultaneous Tests for General Linear Hypotheses

Fit: lmer(formula = Fd ~ gerGT + (1 | Host), data = FdAll, REML = T)

Linear Hypotheses:
                    Estimate Std. Error t value Pr(>|t|)
C2 - C1 == 0   9.0064     2.1083   4.272  < 0.001 ***
C2 - G1 == 0   7.4620     1.9491   3.828  0.00178 **
C2 - G2 == 0  -1.5780     1.8971  -0.832  0.95944
C2 - H2 == 0   0.2626     3.2781   0.080  1.00000
C2 - H1 == 0  -3.9661     2.2516  -1.761  0.47836
C1 - G1 == 0  -1.5444     1.9627  -0.787  0.96801
C1 - G2 == 0 -10.5845     1.8913  -5.596  < 0.001 ***
C1 - H2 == 0  -8.7438     3.2703  -2.674  0.07700 .
C1 - H1 == 0 -12.9726     2.2273  -5.824  < 0.001 ***
G1 - G2 == 0  -9.0401     1.7181  -5.262  < 0.001 ***
G1 - H2 == 0  -7.1994     3.1658  -2.274  0.19673
G1 - H1 == 0 -11.4282     2.0885  -5.472  < 0.001 ***
G2 - H2 == 0   1.8406     3.1534   0.584  0.99161
```

```
G2 - H1 == 0  -2.3881     2.0532  -1.163  0.84753
H2 - H1 == 0  -4.2287     3.3292  -1.270  0.79285
---
Signif. codes:  0 ‘*’ 0.001 ‘’ 0.01 ‘*’ 0.05 ‘.’ 0.1 ‘ ’ 1
(Adjusted p values reported -- single-step method)
```

2

```
        Simultaneous Tests for General Linear Hypotheses

Fit: lmer(formula = Flower ~ gerGT + (1 | Host), data = FdAll, REML = T)

Linear Hypotheses:
                     Estimate Std. Error t value Pr(>|t|)
C2 - C1 == 0   3.1182     1.8338   1.700   0.5189
C2 - G1 == 0   5.6676     1.6953   3.343   0.0104 *
C2 - G2 == 0  -0.8171     1.6499  -0.495   0.9961
C2 - H2 == 0  -2.9667     2.8516  -1.040   0.8994
C2 - H1 == 0   1.9125     1.9583   0.977   0.9215
C1 - G1 == 0   2.5494     1.7073   1.493   0.6573
C1 - G2 == 0  -3.9353     1.6450  -2.392   0.1520
C1 - H2 == 0  -6.0849     2.8450  -2.139   0.2574
C1 - H1 == 0  -1.2057     1.9372  -0.622   0.9887
G1 - G2 == 0  -6.4847     1.4944  -4.339   <0.001 ***
G1 - H2 == 0  -8.6343     2.7539  -3.135   0.0206 *
G1 - H1 == 0  -3.7551     1.8166  -2.067   0.2943
G2 - H2 == 0  -2.1496     2.7434  -0.784   0.9686
G2 - H1 == 0   2.7296     1.7859   1.528   0.6342
H2 - H1 == 0   4.8792     2.8959   1.685   0.5295
---
Signif. codes:  0 ‘*’ 0.001 ‘’ 0.01 ‘*’ 0.05 ‘.’ 0.1 ‘ ’ 1
(Adjusted p values reported -- single-step method)
```

3

```
        Simultaneous Tests for General Linear Hypotheses

Fit: lmer(formula = log(Plant.height) ~ gerGT + Transplant + (1 |
       Host), data = FdAll, REML = T)

Linear Hypotheses:
                      Estimate Std. Error t value Pr(>|t|)
C2 - C1 == 0  0.065164   0.059561   1.094    0.878
C2 - G1 == 0 -0.224798   0.054823  -4.100   <0.001 ***
C2 - G2 == 0 -0.007977   0.053325  -0.150    1.000
C2 - H2 == 0 -0.068418   0.092264  -0.742    0.975
C2 - H1 == 0  0.319884   0.063568   5.032   <0.001 ***
C1 - G1 == 0 -0.289961   0.055350  -5.239   <0.001 ***
C1 - G2 == 0 -0.073140   0.053584  -1.365    0.738
C1 - H2 == 0 -0.133582   0.092052  -1.451    0.685
C1 - H1 == 0  0.254721   0.063713   3.998   <0.001 ***
G1 - G2 == 0  0.216821   0.048379   4.482   <0.001 ***
G1 - H2 == 0  0.156380   0.089074   1.756    0.482
G1 - H1 == 0  0.544682   0.059228   9.196   <0.001 ***
G2 - H2 == 0 -0.060441   0.088838  -0.680    0.983
G2 - H1 == 0  0.327861   0.057956   5.657   <0.001 ***
H2 - H1 == 0  0.388302   0.094090   4.127   <0.001 ***
---
Signif. codes:  0 ‘*’ 0.001 ‘’ 0.01 ‘*’ 0.05 ‘.’ 0.1 ‘ ’ 1
(Adjusted p values reported -- single-step method)
```

4

```
        Simultaneous Tests for General Linear Hypotheses
```

```
Fit: lmer(formula = log(Height) ~ gerGT + Transplant + (1 | Host),
       data = FdAll, REML = T)

Linear Hypotheses:
                     Estimate Std. Error t value Pr(>|t|)
C2 - C1 == 0  0.03983    0.07882   0.505   0.9957
C2 - G1 == 0 -0.40317    0.07203  -5.597   <0.001 ***
C2 - G2 == 0 -0.06212    0.06960  -0.893   0.9452
C2 - H2 == 0 -0.07277    0.12512  -0.582   0.9917
C2 - H1 == 0  0.27149    0.08682   3.127   0.0209 *
C1 - G1 == 0 -0.44300    0.07390  -5.994   <0.001 ***
C1 - G2 == 0 -0.10195    0.07082  -1.439   0.6909
C1 - H2 == 0 -0.11259    0.12547  -0.897   0.9439
C1 - H1 == 0  0.23166    0.08753   2.647   0.0819 .
G1 - G2 == 0  0.34105    0.06360   5.362   <0.001 ***
G1 - H2 == 0  0.33040    0.12128   2.724   0.0670 .
G1 - H1 == 0  0.67466    0.08165   8.262   <0.001 ***
G2 - H2 == 0 -0.01064    0.12071  -0.088   1.0000
G2 - H1 == 0  0.33361    0.07959   4.192   <0.001 ***
H2 - H1 == 0  0.34425    0.12904   2.668   0.0778 .
---
Signif. codes:  0 ‘*’ 0.001 ‘’ 0.01 ‘*’ 0.05 ‘.’ 0.1 ‘ ’ 1
(Adjusted p values reported -- single-step method)
```

5

```
        Simultaneous Tests for General Linear Hypotheses

Fit: lmer(formula = log(1 + Reproductive.nodes) ~ gerGT + Transplant +
       (1 | Host), data = FdAll, REML = T)

Linear Hypotheses:
                     Estimate Std. Error t value Pr(>|t|)
C2 - C1 == 0  0.32497    0.09775   3.325  0.01101 *
C2 - G1 == 0  0.20349    0.08911   2.284  0.19146
C2 - G2 == 0  0.29909    0.08629   3.466  0.00684 **
C2 - H2 == 0  0.56422    0.15708   3.592  0.00426 **
C2 - H1 == 0  0.74632    0.11264   6.626  < 0.001 ***
C1 - G1 == 0 -0.12148    0.09166  -1.325  0.76016
C1 - G2 == 0 -0.02588    0.08804  -0.294  0.99968
C1 - H2 == 0  0.23925    0.15712   1.523  0.63599
C1 - H1 == 0  0.42135    0.11301   3.728  0.00269 **
G1 - G2 == 0  0.09560    0.07877   1.214  0.82148
G1 - H2 == 0  0.36073    0.15245   2.366  0.16024
G1 - H1 == 0  0.54283    0.10664   5.090  < 0.001 ***
G2 - H2 == 0  0.26514    0.15178   1.747  0.48593
G2 - H1 == 0  0.44723    0.10442   4.283  < 0.001 ***
H2 - H1 == 0  0.18210    0.16458   1.106  0.87198
---
Signif. codes:  0 ‘*’ 0.001 ‘’ 0.01 ‘*’ 0.05 ‘.’ 0.1 ‘ ’ 1
(Adjusted p values reported -- single-step method)
```

6

```
        Simultaneous Tests for General Linear Hypotheses

Fit: lmer(formula = Corolla.length ~ gerGT + (1 | Host), data = FdAll,
       REML = T)

Linear Hypotheses:
                      Estimate Std. Error t value Pr(>|t|)
C2 - C1 == 0  0.882641   0.091907   9.604   <0.001 ***
C2 - G1 == 0 -0.061978   0.084967  -0.729    0.977
C2 - G2 == 0 -0.009968   0.082697  -0.121    1.000
C2 - H2 == 0  1.908218   0.142909  13.353   <0.001 ***
C2 - H1 == 0  1.584933   0.098152  16.148   <0.001 ***
```

```
C1 - G1 == 0 -0.944619   0.085562 -11.040   <0.001 ***
C1 - G2 == 0 -0.892609   0.082448 -10.826   <0.001 ***
C1 - H2 == 0  1.025577   0.142574   7.193   <0.001 ***
C1 - H1 == 0  0.702292   0.097094   7.233   <0.001 ***
G1 - G2 == 0  0.052010   0.074899   0.694    0.982
G1 - H2 == 0  1.970196   0.138014  14.275   <0.001 ***
G1 - H1 == 0  1.646911   0.091046  18.089   <0.001 ***
G2 - H2 == 0  1.918186   0.137478  13.953   <0.001 ***
G2 - H1 == 0  1.594901   0.089506  17.819   <0.001 ***
H2 - H1 == 0 -0.323285   0.145136  -2.227    0.216
---
Signif. codes:  0 ‘*’ 0.001 ‘’ 0.01 ‘*’ 0.05 ‘.’ 0.1 ‘ ’ 1
(Adjusted p values reported -- single-step method)
```

7

```
        Simultaneous Tests for General Linear Hypotheses

Fit: lmer(formula = Nodes.to.flower ~ gerGT + (1 | Host), data = FdAll,
       REML = T)

Linear Hypotheses:
                     Estimate Std. Error t value Pr(>|t|)
C2 - C1 == 0   0.6138     0.1541   3.984   <0.001 ***
C2 - G1 == 0   1.5587     0.1424  10.946   <0.001 ***
C2 - G2 == 0   0.1655     0.1397   1.185   0.8373
C2 - H2 == 0   1.1529     0.2369   4.866   <0.001 ***
C2 - H1 == 0   1.6124     0.1641   9.827   <0.001 ***
C1 - G1 == 0   0.9449     0.1406   6.719   <0.001 ***
C1 - G2 == 0  -0.4483     0.1379  -3.251   0.0143 *
C1 - H2 == 0   0.5391     0.2359   2.286   0.1923
C1 - H1 == 0   0.9986     0.1625   6.144   <0.001 ***
G1 - G2 == 0  -1.3932     0.1247 -11.169   <0.001 ***
G1 - H2 == 0  -0.4058     0.2284  -1.776   0.4687
G1 - H1 == 0   0.0537     0.1515   0.354   0.9992
G2 - H2 == 0   0.9874     0.2268   4.355   <0.001 ***
G2 - H1 == 0   1.4469     0.1490   9.710   <0.001 ***
H2 - H1 == 0   0.4595     0.2425   1.895   0.3935
---
Signif. codes:  0 ‘*’ 0.001 ‘’ 0.01 ‘*’ 0.05 ‘.’ 0.1 ‘ ’ 1
(Adjusted p values reported -- single-step method)
```

8

```
        Simultaneous Tests for General Linear Hypotheses

Fit: lmer(formula = log(lir) ~ gerGT + (1 | Host), data = FdAll, REML = T)

Linear Hypotheses:
                     Estimate Std. Error t value Pr(>|t|)
C2 - C1 == 0  0.57830    0.07344   7.875  < 0.001 ***
C2 - G1 == 0  0.85583    0.06789  12.606  < 0.001 ***
C2 - G2 == 0  0.33111    0.06609   5.010  < 0.001 ***
C2 - H2 == 0  1.22982    0.11416  10.773  < 0.001 ***
C2 - H1 == 0  0.84126    0.07843  10.727  < 0.001 ***
C1 - G1 == 0  0.27753    0.06835   4.061  < 0.001 ***
C1 - G2 == 0 -0.24719    0.06588  -3.752  0.00234 **
C1 - H2 == 0  0.65151    0.11386   5.722  < 0.001 ***
C1 - H1 == 0  0.26296    0.07758   3.389  0.00889 **
G1 - G2 == 0 -0.52472    0.05984  -8.769  < 0.001 ***
G1 - H2 == 0  0.37398    0.11023   3.393  0.00876 **
G1 - H1 == 0 -0.01457    0.07274  -0.200  0.99995
G2 - H2 == 0  0.89871    0.10978   8.186  < 0.001 ***
G2 - H1 == 0  0.51015    0.07151   7.134  < 0.001 ***
H2 - H1 == 0 -0.38855    0.11594  -3.351  0.01020 *
---
Signif. codes:  0 ‘*’ 0.001 ‘’ 0.01 ‘*’ 0.05 ‘.’ 0.1 ‘ ’ 1
```

```
(Adjusted p values reported -- single-step method)
```

9

```
         Simultaneous Tests for General Linear Hypotheses

Fit: lmer(formula = log(Number.of.leaf.teeth..exc..tip.) ~ gerGT + 
    Transplant + (1 | Host), data = FdAll)

Linear Hypotheses:
                     Estimate Std. Error t value Pr(>|t|)    
C2 - C1 == 0  0.44314    0.04349  10.189  < 0.001 ***
C2 - G1 == 0  0.28003    0.04003   6.995  < 0.001 ***
C2 - G2 == 0  0.21931    0.03894   5.632  < 0.001 ***
C2 - H2 == 0  0.19283    0.06736   2.863  0.04608 *  
C2 - H1 == 0  0.54739    0.04642  11.793  < 0.001 ***
C1 - G1 == 0 -0.16312    0.04041  -4.036  < 0.001 ***
C1 - G2 == 0 -0.22384    0.03913  -5.721  < 0.001 ***
C1 - H2 == 0 -0.25032    0.06720  -3.725  0.00261 ** 
C1 - H1 == 0  0.10424    0.04652   2.241  0.21074    
G1 - G2 == 0 -0.06072    0.03532  -1.719  0.50654    
G1 - H2 == 0 -0.08720    0.06502  -1.341  0.75245    
G1 - H1 == 0  0.26736    0.04324   6.183  < 0.001 ***
G2 - H2 == 0 -0.02648    0.06485  -0.408  0.99845    
G2 - H1 == 0  0.32808    0.04232   7.753  < 0.001 ***
H2 - H1 == 0  0.35456    0.06869   5.162  < 0.001 ***
---
Signif. codes:  0 ‘*’ 0.001 ‘’ 0.01 ‘*’ 0.05 ‘.’ 0.1 ‘ ’ 1
(Adjusted p values reported -- single-step method)
```

10

```
         Simultaneous Tests for General Linear Hypotheses

Fit: lmer(formula = Capsule.width ~ gerGT + (1 | Host), data = FdAll, 
    REML = T)

Linear Hypotheses:
                     Estimate Std. Error t value Pr(>|t|)    
C2 - C1 == 0 -0.10501    0.06424  -1.635    0.559    
C2 - G1 == 0 -0.30154    0.05714  -5.277   <0.001 ***
C2 - G2 == 0 -0.03239    0.05679  -0.570    0.992    
C2 - H2 == 0  0.45742    0.10756   4.253   <0.001 ***
C2 - H1 == 0  0.63594    0.08252   7.707   <0.001 ***
C1 - G1 == 0 -0.19653    0.06083  -3.231    0.015 *  
C1 - G2 == 0  0.07262    0.05989   1.212    0.821    
C1 - H2 == 0  0.56243    0.10802   5.207   <0.001 ***
C1 - H1 == 0  0.74095    0.08183   9.054   <0.001 ***
G1 - G2 == 0  0.26915    0.05227   5.149   <0.001 ***
G1 - H2 == 0  0.75896    0.10532   7.206   <0.001 ***
G1 - H1 == 0  0.93748    0.07939  11.809   <0.001 ***
G2 - H2 == 0  0.48981    0.10503   4.663   <0.001 ***
G2 - H1 == 0  0.66833    0.07942   8.415   <0.001 ***
H2 - H1 == 0  0.17852    0.11704   1.525    0.632    
---
Signif. codes:  0 ‘*’ 0.001 ‘’ 0.01 ‘*’ 0.05 ‘.’ 0.1 ‘ ’ 1
(Adjusted p values reported -- single-step method)
```

11

```
         Simultaneous Tests for General Linear Hypotheses

Fit: lmer(formula = Capsule.Height ~ gerGT + (1 | Host), data = FdAll, 
    REML = T)
```

```
Linear Hypotheses:
                        Estimate Std. Error t value Pr(>|t|)
C2 - C1 == 0  0.305012   0.151896   2.008  0.32285
C2 - G1 == 0 -0.581231   0.135065  -4.303  < 0.001 ***
C2 - G2 == 0 -0.589118   0.134242  -4.388  < 0.001 ***
C2 - H2 == 0  1.220697   0.254288   4.800  < 0.001 ***
C2 - H1 == 0  1.007755   0.195165   5.164  < 0.001 ***
C1 - G1 == 0 -0.886243   0.143848  -6.161  < 0.001 ***
C1 - G2 == 0 -0.894130   0.141612  -6.314  < 0.001 ***
C1 - H2 == 0  0.915684   0.255408   3.585  0.00448 **
C1 - H1 == 0  0.702743   0.193525   3.631  0.00382 **
G1 - G2 == 0 -0.007887   0.123574  -0.064  1.00000
G1 - H2 == 0  1.801928   0.248991   7.237  < 0.001 ***
G1 - H1 == 0  1.588987   0.187762   8.463  < 0.001 ***
G2 - H2 == 0  1.809815   0.248349   7.287  < 0.001 ***
G2 - H1 == 0  1.596874   0.187872   8.500  < 0.001 ***
H2 - H1 == 0 -0.212941   0.276651  -0.770  0.97031
---
Signif. codes:  0 ‘*’ 0.001 ‘’ 0.01 ‘*’ 0.05 ‘.’ 0.1 ‘ ’ 1
(Adjusted p values reported -- single-step method)
```

SUPPLEMENTAL TEXT 2 – MODELS FOR K-MER SPECTRA

A k-mer spectrum shows the distribution of counts of each individual k-mer (a small sequence word of length *k*) in a given data set. If the data set is generated from shotgun sequencing data of *one* individual, then the k-mer spectrum will reflect the genetic diversity in that individual's genome. The number of "peaks" in such a k-mer spectrum corresponds to the individual's ploidy level. A spectrum of a polyploid individual has four peaks, which we label 1x, 2x, 3x, and 4x. Schematics of k-mer spectra are shown in Figure 4A of the main text.

Tetraploids are generally classed into either of two model categories: autotetrapolyploid or allotetraploid (but there are intermediates, see Stift et al. (2008)). Autotetraploids show tetrasomic inheritance, where recombination may happen between any pair of homologous chromosomes. Allotetrapolyploids contain two diverged (homoeologous) pairs of genomes show. They show disomic inheritance where recombination may happen only within each homologous group, maintaining (and increasing over time) the genetic divergence between these groups. This causes different shapes of autotetraploid and allotetraploid k-mer spectra. For instance, allotetraploids should show a more pronounced 2x peak than autopolyploids, resulting from diverged alleles fixed in both homoeologous groups. The expectation for the shape of a k-mer spectrum can be obtained by combining the number of k-mers expected in each peak with a suitable distribution such as the negative binomial distribution as parameterised by Vurture et al. (2017). For autopolyploids, the (relative) number of k-mers expected in each peak can be obtained by using Ewens' (1972) sampling formula for the frequency of certain allelic configurations and by accounting for how many k-mers each configuration contributes to each peak of the k-mer spectrum. For allopolyploids, the homoeologous genomes can be treated like a pair of diverged sub-populations. The expectation for allelic configurations can then be obtained by the method of Lohse et al. (2011, 2016). The terms for the relative contributions to k-mer peaks in autotetraploids are:

$\frac{4\theta}{3+\theta}$ for the 1x peak,

$\frac{6\theta}{6+5\theta+\theta^2}$ for the 2x peak,

$\frac{8\theta}{6+11\theta+6\theta^2+\theta^3}$ for the 3x peak, and

$\frac{6}{6+11\theta+6\theta^2+\theta^3}$ for the 4x peak.

They all depend on θ, the population-scaled mutation rate per k-mer ($\theta = 4N_e\mu$). From these formulae it can be shown that in an autotetraploid, the 2x peak can never contain more k-mers than the 1x peak: the inequality $\frac{4\theta}{3+\theta} < \frac{6\theta}{6+5\theta+\theta^2}$, which can be transformed into $6\theta + 17\theta^2 + 11\theta^3 + 2\theta^4 < 0$, cannot be true as long as the mutation rate is positive.

To compute the k-mer contributions in the allopolyploid model, an additional parameter T is needed, the divergence time between the two homoeologous sub-genomes (T is scaled in units of twice the effective population size N_e). The terms for the relative contributions in allopolyploids are:

$\frac{4e^{-3T\theta}\theta(-2e^{T(-2+\theta)}+e^{3T\theta}(2+\theta)^2(3+\theta)-2e^{2T\theta}(3+4\theta+\theta^2))}{(1+\theta)(2+\theta)^2(3+\theta)}$ for the 1x peak,

$\frac{2e^{-\frac{1}{2}T(4+5\theta)}(6e^{\frac{T\theta}{2}}\theta+e^{\frac{1}{2}T(4+5\theta)}(2+\theta)^2(3+\theta)+2e^{2T+\frac{3T\theta}{2}}(-6-8\theta+\theta^2+\theta^3))}{(1+\theta)(2+\theta)^2(3+\theta)}$ for the 2x peak,

$\frac{8e^{-T(3+4\theta)}\theta(-e^{T+2T\theta}+e^{3T(1+\theta)}(3+\theta))}{(1+\theta)(2+\theta)^2(3+\theta)}$ for the 3x peak, and

$\frac{2e^{-2T(1+\theta)}(\theta+2e^{T(2+\theta)}(3+\theta))}{(1+\theta)(2+\theta)^2(3+\theta)}$ for the 4x peak.

Ignoring any shared polymorphisms at the time of split, the divergence between the sub-genomes (per k-mer) can be computed as θT. This is reasonable, in particular for higher values of T, where most divergence will be due to alleles arising after the split of the sub-populations. A Mathematica notebook containing the code to derive the terms above is supplied in the Zenodo data set published alongside this paper.

In mixed maters, like many plant species, the estimate for θ obtained from one individual may be much lower than the diversity found in the population, as for H_o and H_e. Because the sub-genome divergence time, T, is scaled by N_e, which itself depends on θ, the estimate may not be meaningful as an estimate of the actual time of the split between the sub-genome progenitors. However, the average divergence per k-mer, $T\theta$, is not affected by this. When $T\theta$ is divided by *k*, gives the average per-nucleotide sub-genome divergence.

USAGE EXAMPLE – AUTOTETRAPLOID

In this study, we applied Tetmer to data from tetraploid species of *Euphrasia*, and we found that the allotetraploid model fitted well (Suppl. Text 3). We then looked to identify suitable sequencing data sets of autotetraploids (high coverage, low contamination, one single genetic individual per sequencing library). We found that the autotetraploid model fitted well to data from tetraploid yeast sequence read archive SRR3265401, resulting in a (non-repetitive) genome size estimate of 11.1 Mbp and a population-scale mutation rate per k-mer of 0.1171, which corresponds to 0.4% per nucleotide.

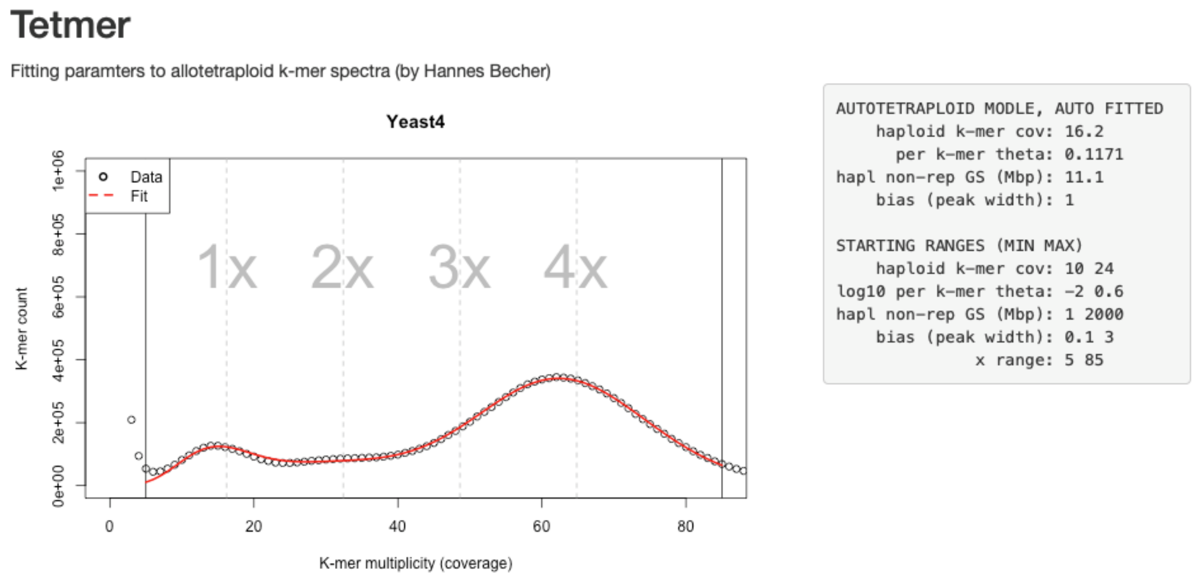

USAGE EXAMPLE – ALLOTETRAPLOID

We applied Tetmer to a public data set of *Capsella bursa-pastoris* (SRR5412136). As expected, the allotetraploid model fits well. The divergence between the sub-genomes is estimated to be 1.125 (per k-mer of length 27). This corresponds to 4.2% sub-genome divergence per nucleotide.

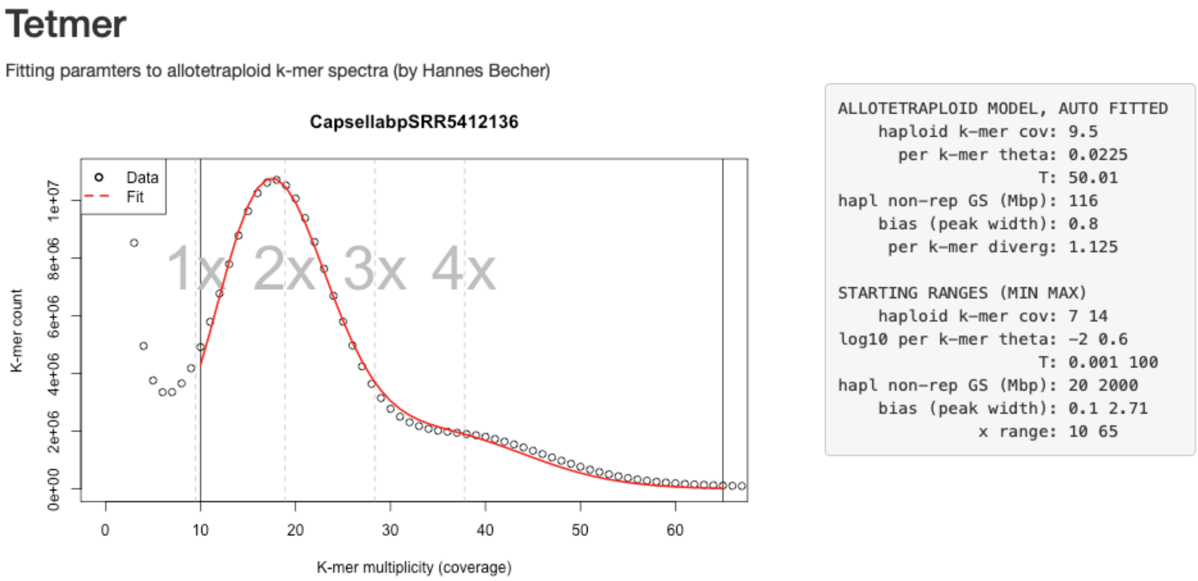

REFERENCES

Ewens, W. J. (1972). The sampling theory of selectively neutral alleles. *Theor. Popul. Biol.* **3**:87–112.

Lohse, K., Harrison, R. J., and Barton, N. H. (2011). A General Method for Calculating Likelihoods Under the Coalescent Process. *Genetics* **189**:977–987.

Lohse, K., Chmelik, M., Martin, S. H., and Barton, N. H. (2016). Efficient Strategies for Calculating Blockwise Likelihoods Under the Coalescent. *Genetics* **202**:775–786.

Stift, M., Berenos, C., Kuperus, P., and van Tienderen, P. H. (2008). Segregation models for disomic, tetrasomic and intermediate inheritance in tetraploids: a general procedure applied to Rorippa (yellow cress) microsatellite data. *Genetics* **179**:2113–23.

Vurture, G. W., Sedlazeck, F. J., Nattestad, M., Underwood, C. J., Fang, H., Gurtowski, J., and Schatz, M. C. (2017). GenomeScope: fast reference-free genome profiling from short reads.

Bioinformatics **33**:2202–2204.

SUPPLEMENTAL TEXT 3 – GENOME PROFILING

Note: All analyses were run with k-mers of 27 nt length. For Smudgeplot and Genomescope2.0, the haploid k-mer coverage was supplied as an input parameter.

The background of Tetmer is explained in Supplementary Text 2.

Smudgeplot is a package maintained by Kamil Jaron (https://github.com/KamilSJaron/smudgeplot). It analyses pairs of k-mers that differ by one nucleotide (k-mer A and k-mer B). It then plots the summed coverage of k-mers A and B on the y-axis and the proportion of the coverage of k-mer B in the total coverage on the x-axis (as indicated by the axis labels).

Euphrasia anglica (AN, diploid)

GenomeScpe2.0

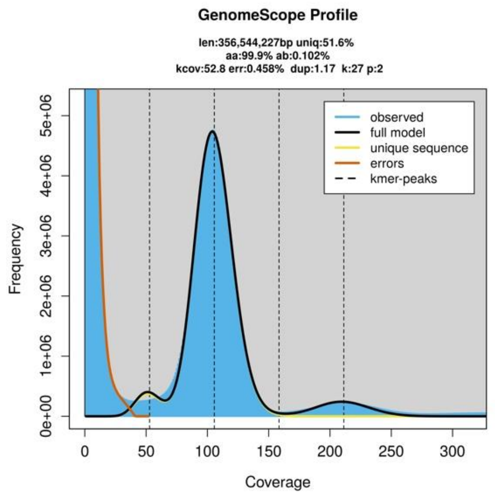


```
GenomeScope version 2.0
input file = /mnt/1TBSSD/Eukmers/numsE030
output directory = E030
p = 2
k = 27
initial kmercov estimate = 53

property                      min               max
Homozygous (aa)               99.8858%          99.911%
Heterozygous (ab)             0.0889549%        0.114179%
Genome Haploid Length         355,898,845 bp    356,544,227 bp
Genome Repeat Length          172,096,499 bp    172,408,576 bp
Genome Unique Length          183,802,346 bp    184,135,651 bp
Model Fit                     56.7642%          94.5677%
Read Error Rate               0.457507%         0.457507%
```

Smudgeplot

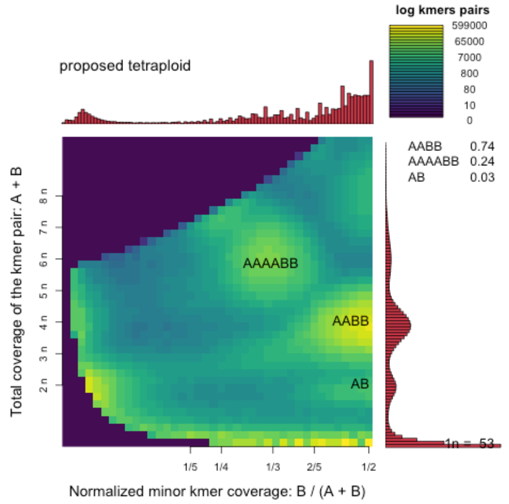

Tetmer

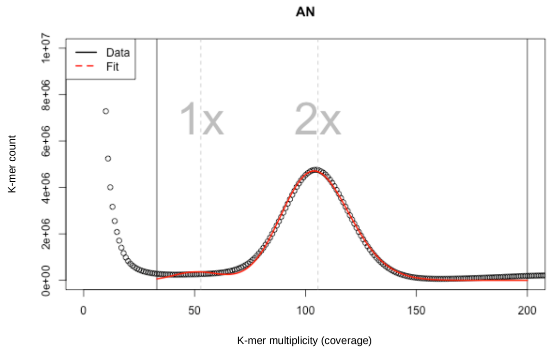


```
DIPLOID MODEL, AUTO FITTED
     haploid k-mer cov: 52.8
       per k-mer theta: 0.0273
hapl non-rep GS (Mbp): 185.3
     bias (peak width): 1.2

STARTING RANGES (MIN MAX)
     haploid k-mer cov: 19 59
log10 per k-mer theta: -2.75 0.6
hapl non-rep GS (Mbp): 20 2000
     bias (peak width): 0.1 3
              x range: 33 200
```

Euphrasia vigursii (VI, diploid)

GenomeScpe2.0

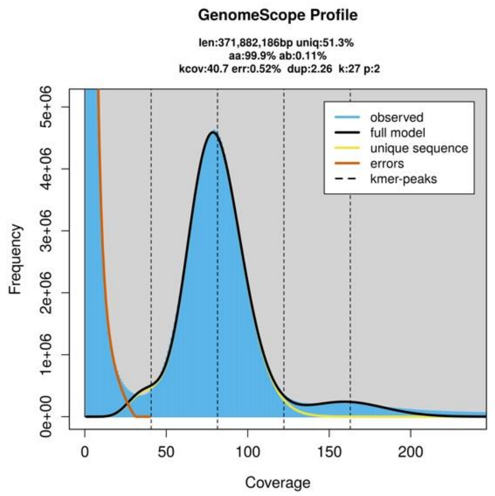


```
GenomeScope version 2.0
input file = /mnt/1TBSSD/Eukmers/numsE031
output directory = E031
p = 2
k = 27
initial kmercov estimate = 41

property                      min               max
Homozygous (aa)               99.8731%          99.9071%
Heterozygous (ab)             0.0928852%        0.126899%
Genome Haploid Length         370,710,233 bp    371,882,186 bp
Genome Repeat Length          180,417,372 bp    180,987,738 bp
Genome Unique Length          190,292,861 bp    190,894,447 bp
Model Fit                     56.9501%          96.4987%
Read Error Rate               0.519548%         0.519548%
```

Smudgeplot

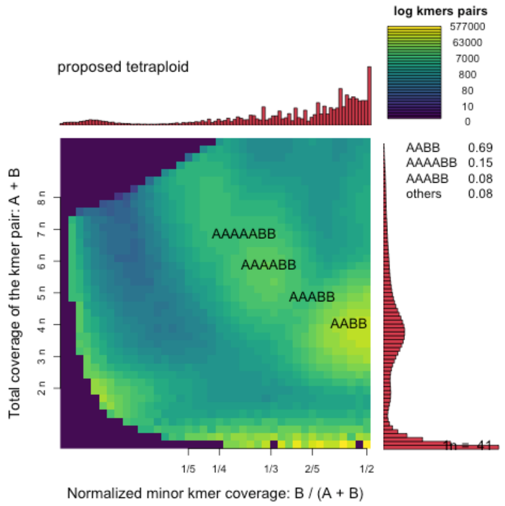

Tetmer

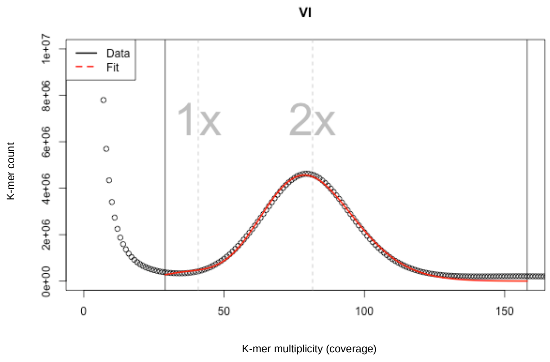


```
DIPLOID MODEL, AUTO FITTED
    haploid k-mer cov: 40.8
      per k-mer theta: 0.0294
hapl non-rep GS (Mbp): 192.6
    bias (peak width): 2.4

STARTING RANGES (MIN MAX)
    haploid k-mer cov: 10 55
log10 per k-mer theta: -2.4 0.6
hapl non-rep GS (Mbp): 20 2000
    bias (peak width): 0.1 3
              x range: 29 158
```

Euphrasia rivularis (RI, diploid)

GenomeScpe2.0

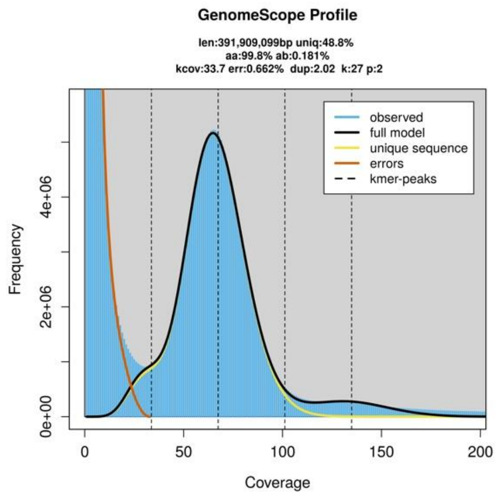


```
GenomeScope version 2.0
input file = /mnt/1TBSSD/Eukmers/numsE032
output directory = E032
p = 2
k = 27
initial kmercov estimate = 33

property                      min               max
Homozygous (aa)               99.7973%          99.8408%
Heterozygous (ab)             0.159239%         0.202729%
Genome Haploid Length         390,392,793 bp    391,909,099 bp
Genome Repeat Length          199,832,645 bp    200,608,806 bp
Genome Unique Length          190,560,148 bp    191,300,294 bp
Model Fit                     54.4646%          97.309%
Read Error Rate               0.66216%          0.66216%
```

Smudgeplot

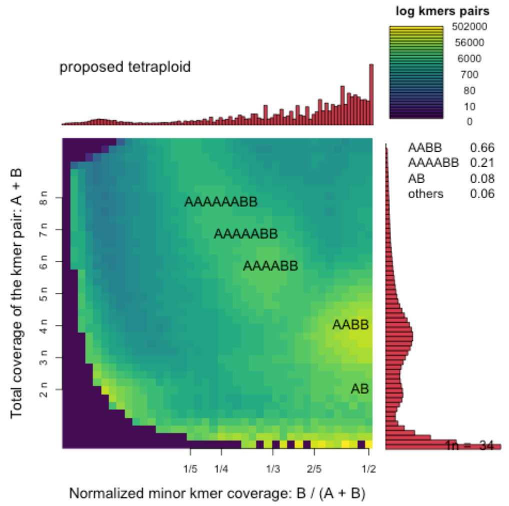

Tetmer

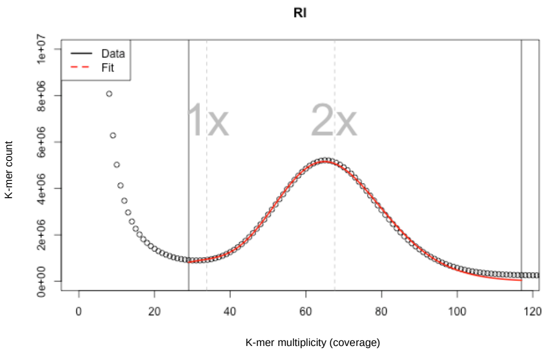


```
DIPLOID MODEL, AUTO FITTED
     haploid k-mer cov: 33.8
       per k-mer theta: 0.0542
hapl non-rep GS (Mbp): 193.1
     bias (peak width): 2.1

STARTING RANGES (MIN MAX)
     haploid k-mer cov: 10 41
log10 per k-mer theta: -2 0.6
hapl non-rep GS (Mbp): 20 2000
     bias (peak width): 0.1 3
               x range: 29 117
```

EUPHRASIA ROSTKOVIANA (RO, DIPLOID)

GENOMESCPE2.0

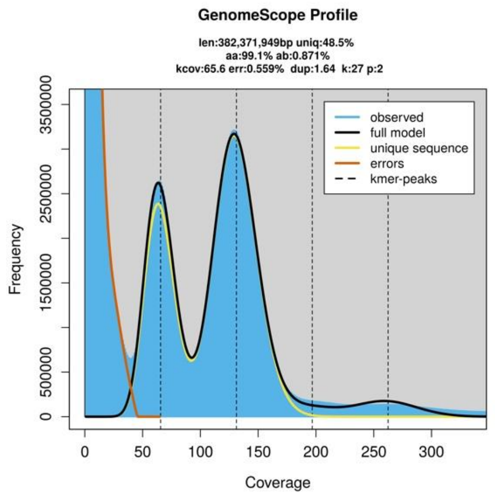


```
GenomeScope version 2.0
input file = /mnt/1TBSSD/Eukmers/numsE040
output directory = E040
p = 2
k = 27
initial kmercov estimate = 66

property                    min               max
Homozygous (aa)             99.1141%          99.1449%
Heterozygous (ab)           0.855141%         0.885937%
Genome Haploid Length       381,376,095 bp    382,371,949 bp
Genome Repeat Length        196,460,140 bp    196,973,139 bp
Genome Unique Length        184,915,956 bp    185,398,811 bp
Model Fit                   55.4459%          94.5013%
Read Error Rate             0.559427%         0.559427%
```

SMUDGEPLOT

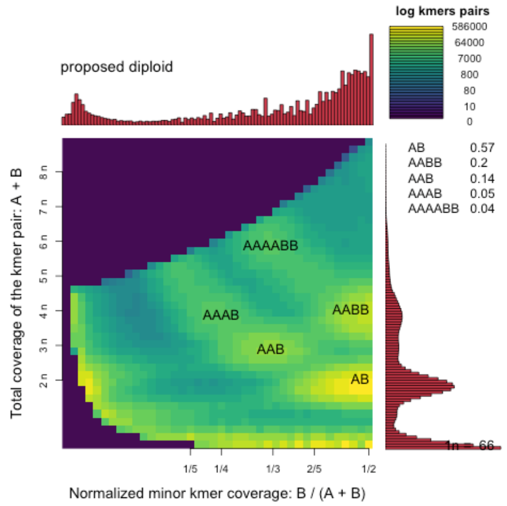

TETMER

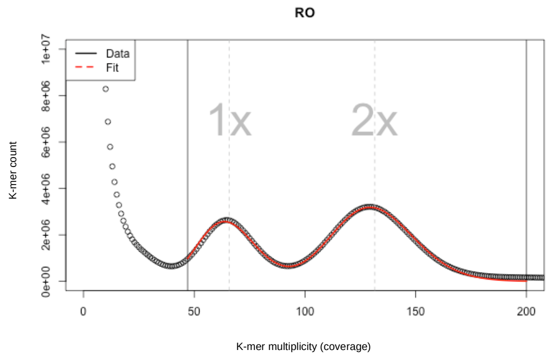


```
DIPLOID MODEL, AUTO FITTED
    haploid k-mer cov: 65.7
      per k-mer theta: 0.2841
hapl non-rep GS (Mbp): 189.5
    bias (peak width): 1.6

STARTING RANGES (MIN MAX)
    haploid k-mer cov: 10 85
log10 per k-mer theta: -2 0.6
hapl non-rep GS (Mbp): 20 2000
    bias (peak width): 0.1 3
              x range: 47 200
```

Euphrasia arctica (A0, tetraploid)

GenomeScpe2.0

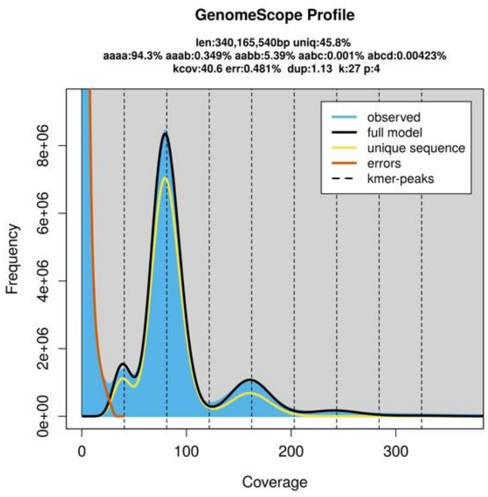


```
GenomeScope version 2.0
input file = /mnt/1TBSSD/Eukmers/numsE028
output directory = E028
p = 4
k = 27
initial kmercov estimate = 40

property                    min                 max
Homozygous (aaaa)           92.4957%            94.7871%
Heterozygous (not aaaa)     5.21291%            7.50425%
aaab                        0%                  1.04367%
aabb                        5.21291%            5.57398%
aabc                        0%                  0.382878%
abcd                        0%                  0.503721%
Genome Haploid Length       339,487,037 bp      340,165,540 bp
Genome Repeat Length        183,904,958 bp      184,272,513 bp
Genome Unique Length        155,582,079 bp      155,893,027 bp
Model Fit                   60.4511%            91.886%
Read Error Rate             0.480646%           0.480646%
```

Smudgeplot

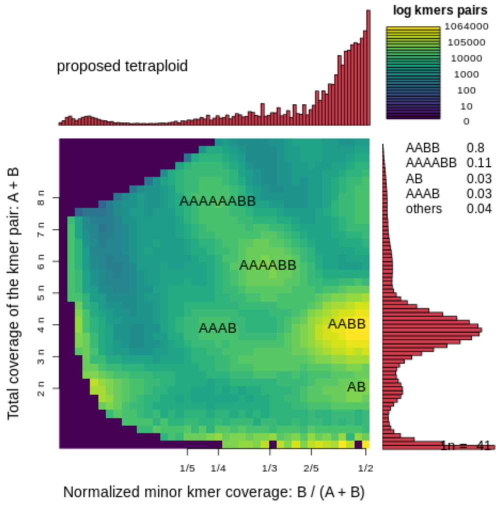

Tetmer

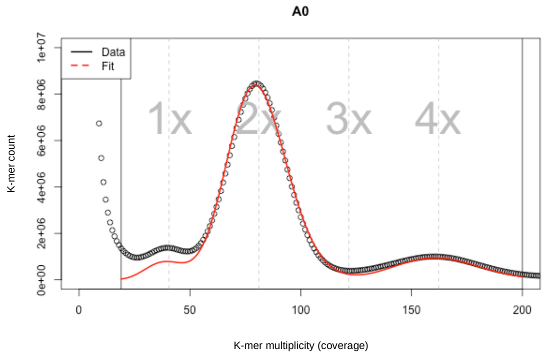


```
ALLOTETRAPLOID MODEL, AUTO FITTED
    haploid k-mer cov: 40.6
      per k-mer theta: 0.0279
                    T: 49.99
hapl non-rep GS (Mbp): 191.2
    bias (peak width): 1.2
     per k-mer diverg: 1.395

STARTING RANGES (MIN MAX)
    haploid k-mer cov: 21 53
log10 per k-mer theta: -2.95 0.6
                    T: 0.001 100
hapl non-rep GS (Mbp): 20 2000
    bias (peak width): 0.1 3
              x range: 19 200
```

Euphrasia arctica (A1, tetraploid)

GenomeScpe2.0

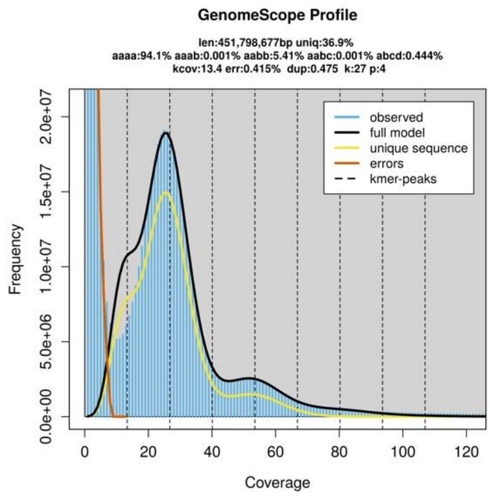


```
GenomeScope version 2.0
input file = /mnt/1TBSSD/Eukmers/numsE027
output directory = E027
p = 4
k = 27
initial kmercov estimate = 13

property                    min              max
Homozygous (aaaa)           91.5436%         95.0556%
Heterozygous (not aaaa)     4.94439%         8.45638%
aaab                        0%               0.790602%
aabb                        4.94439%         5.88006%
aabc                        0%               0.739464%
abcd                        0%               1.04626%
Genome Haploid Length       448,652,997 bp   451,798,677 bp
Genome Repeat Length        283,222,006 bp   285,207,786 bp
Genome Unique Length        165,430,991 bp   166,590,891 bp
Model Fit                   51.1329%         92.7128%
Read Error Rate             0.414595%        0.414595%
```

Smudgeplot

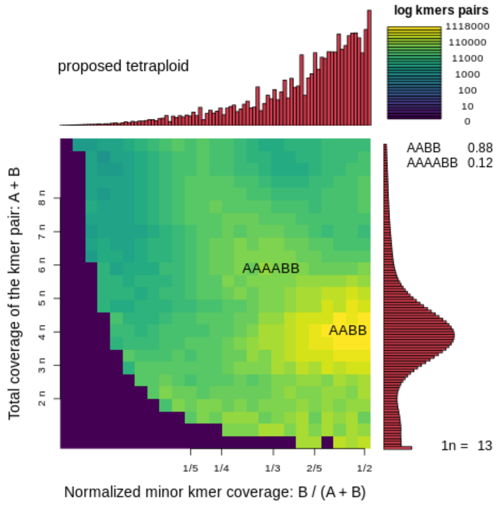

Tetmer

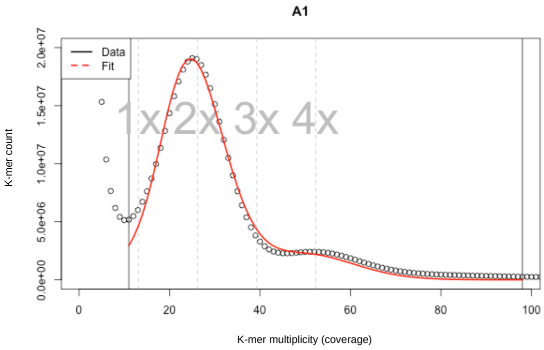


```
ALLOTETRAPLOID MODEL, AUTO FITTED
    haploid k-mer cov: 13.1
      per k-mer theta: 0.028
                    T: 50.01
hapl non-rep GS (Mbp): 224.3
    bias (peak width): 0.9
     per k-mer diverg: 1.399

STARTING RANGES (MIN MAX)
    haploid k-mer cov: 10 18
log10 per k-mer theta: -4 0.6
                    T: 0.001 100
hapl non-rep GS (Mbp): 20 2000
    bias (peak width): 0.1 3
              x range: 11 98
```

EUPHRASIA ARCTICA (A2, TETRAPLOID)

GENOMESCPE2.0

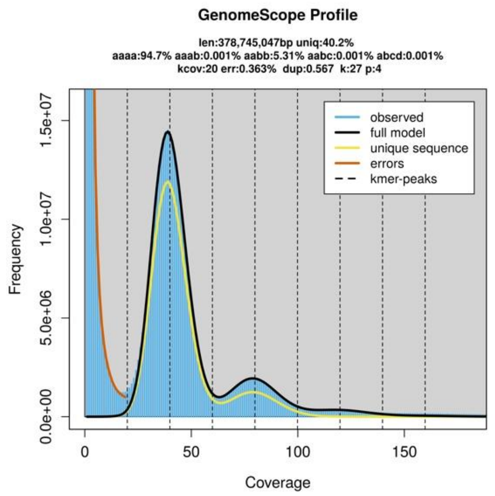


```
GenomeScope version 2.0
input file = /mnt/1TBSSD/Eukmers/numsE026
output directory = E026
p = 4
k = 27
initial kmercov estimate = 20

property                      min              max
Homozygous (aaaa)             92.7655%         94.8998%
Heterozygous (not aaaa)       5.10016%         7.23446%
aaab                          0%               0.759079%
aabb                          5.10016%         5.52219%
aabc                          0%               0.392766%
abcd                          0%               0.560428%
Genome Haploid Length         376,975,633 bp   378,745,047 bp
Genome Repeat Length          225,294,331 bp   226,351,798 bp
Genome Unique Length          151,681,302 bp   152,393,250 bp
Model Fit                     55.8344%         94.6734%
Read Error Rate               0.362751%        0.362751%
```

SMUDGEPLOT

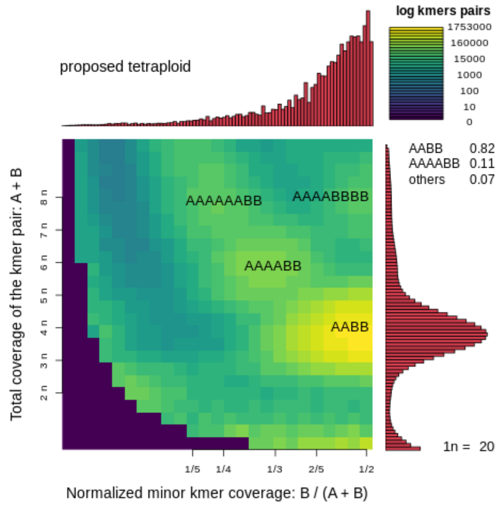

TETMER

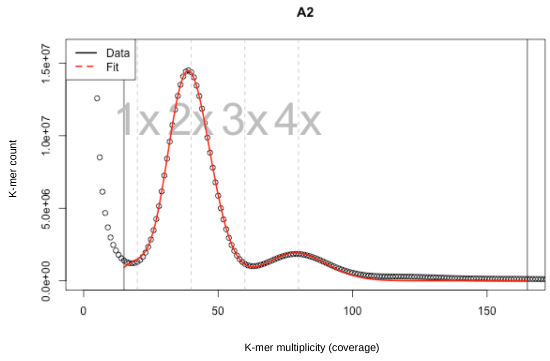


```
ALLOTETRAPLOID MODEL, AUTO FITTED
     haploid k-mer cov: 20
       per k-mer theta: 0.0244
                     T: 52.45
hapl non-rep GS (Mbp): 201.1
     bias (peak width): 0.5
      per k-mer diverg: 1.279

STARTING RANGES (MIN MAX)
     haploid k-mer cov: 10 34
log10 per k-mer theta: -3.2 0.6
                     T: 5 100
hapl non-rep GS (Mbp): 89 373
     bias (peak width): 0.1 3
               x range: 15 165
```

Euphrasia arctica (A3, tetraploid)

GenomeScpe2.0

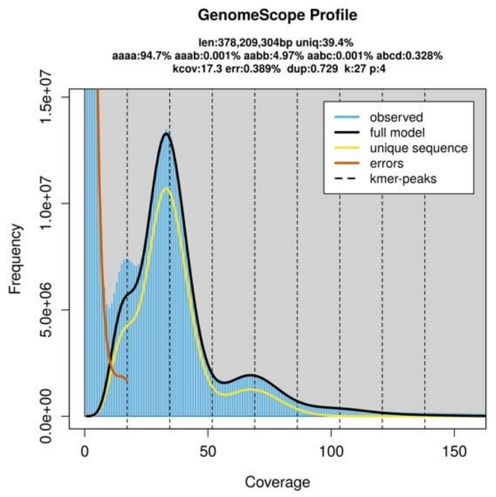


```
GenomeScope version 2.0
input file = /mnt/1TBSSD/Eukmers/numsE036
output directory = E036
p = 4
k = 27
initial kmercov estimate = 17

property                      min               max
Homozygous (aaaa)             92.8383%          95.1968%
Heterozygous (not aaaa)       4.80318%          7.16166%
aaab                          0%                0.584296%
aabb                          4.80318%          5.13048%
aabc                          0%                0.606882%
abcd                          0%                0.839998%
Genome Haploid Length         376,114,344 bp    378,209,304 bp
Genome Repeat Length          227,949,510 bp    229,219,190 bp
Genome Unique Length          148,164,835 bp    148,990,114 bp
Model Fit                     55.5124%          94.4969%
Read Error Rate               0.388542%         0.388542%
```

Smudgeplot

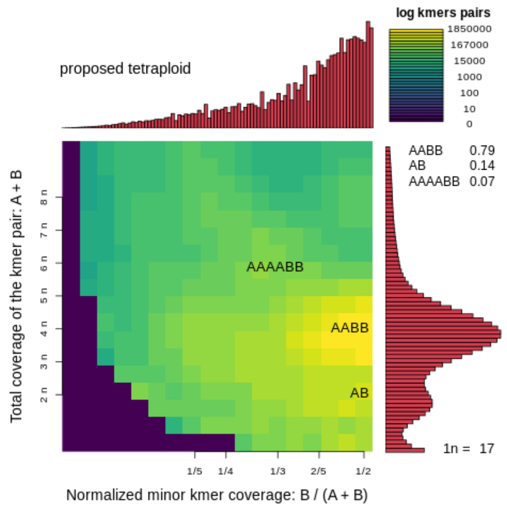

Tetmer

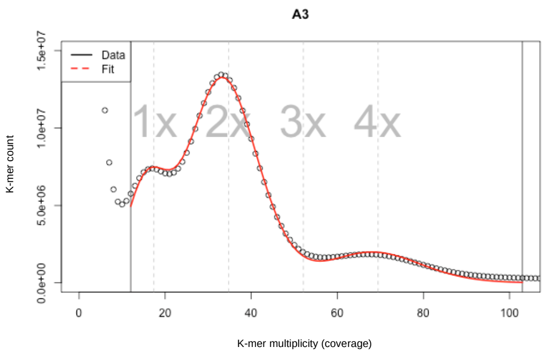


```
ALLOTETRAPLOID MODEL, AUTO FITTED
     haploid k-mer cov: 17.4
       per k-mer theta: 0.1507
                     T: 7.28
hapl non-rep GS (Mbp): 213.1
     bias (peak width): 0.7
      per k-mer diverg: 1.097

STARTING RANGES (MIN MAX)
     haploid k-mer cov: 10 24
log10 per k-mer theta: -2 0.8
                     T: 0.001 100
hapl non-rep GS (Mbp): 20 400
     bias (peak width): 0.01 0.96
              x range: 12 103
```

Euphrasia foulaensis (F1, tetraploid)

GenomeScpe2.0

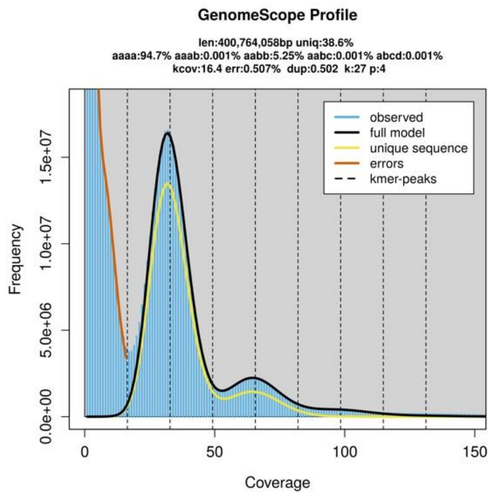


```
GenomeScope version 2.0
input file = /mnt/1TBSSD/Eukmers/numsE025
output directory = E025
p = 4
k = 27
initial kmercov estimate = 16

property                      min               max
Homozygous (aaaa)             91.7601%          94.9911%
Heterozygous (not aaaa)       5.00888%          8.23991%
aaab                          0%                1.08841%
aabb                          5.00888%          5.48638%
aabc                          0%                0.678665%
abcd                          0%                0.986456%
Genome Haploid Length         395,889,466 bp    400,764,058 bp
Genome Repeat Length          243,095,268 bp    246,088,503 bp
Genome Unique Length          152,794,198 bp    154,675,555 bp
Model Fit                     53.763%           93.1246%
Read Error Rate               0.5072%           0.5072%
```

Smudgeplot

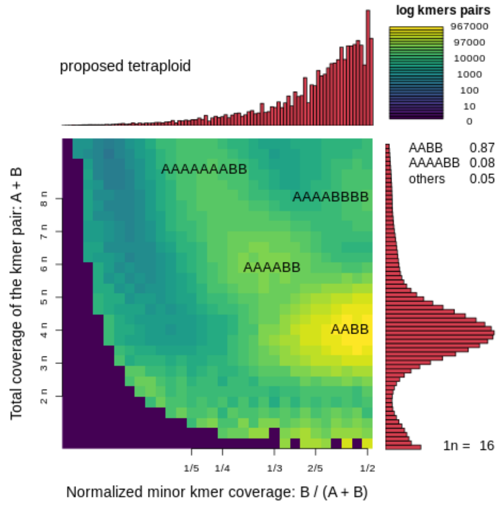

Tetmer

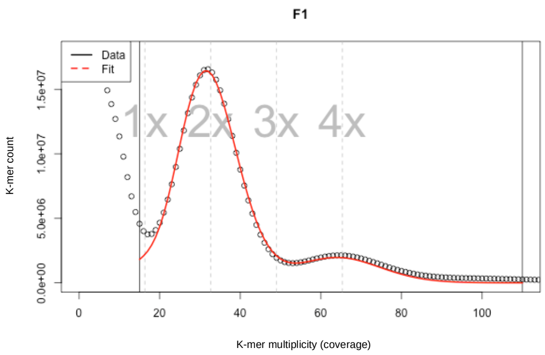


```
ALLOTETRAPLOID MODEL, AUTO FITTED
     haploid k-mer cov: 16.3
       per k-mer theta: 0.0244
                     T: 55.55
hapl non-rep GS (Mbp): 204.1
     bias (peak width): 0.6
      per k-mer diverg: 1.357

STARTING RANGES (MIN MAX)
     haploid k-mer cov: 10 24
log10 per k-mer theta: -3.3 0.6
                     T: 0.001 100
hapl non-rep GS (Mbp): 61 317
     bias (peak width): 0.1 3
              x range: 15 110
```

EUPHRASIA FOULAENSIS (F2, TETRAPLOID)

GENOMESCPE2.0

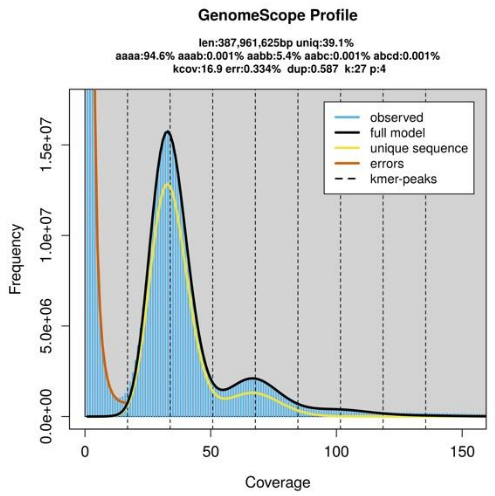


```
GenomeScope version 2.0
input file = /mnt/1TBSSD/Eukmers/numsE024
output directory = E024
p = 4
k = 27
initial kmercov estimate = 16

property                    min                 max
Homozygous (aaaa)           92.3039%            94.9094%
Heterozygous (not aaaa)     5.09063%            7.69614%
aaab                        0%                  0.888598%
aabb                        5.09063%            5.70509%
aabc                        0%                  0.428555%
abcd                        0%                  0.673904%
Genome Haploid Length       384,406,674 bp      387,961,625 bp
Genome Repeat Length        234,199,161 bp      236,365,009 bp
Genome Unique Length        150,207,513 bp      151,596,616 bp
Model Fit                   55.237%             95.2557%
Read Error Rate             0.334242%           0.334242%
```

SMUDGEPLOT

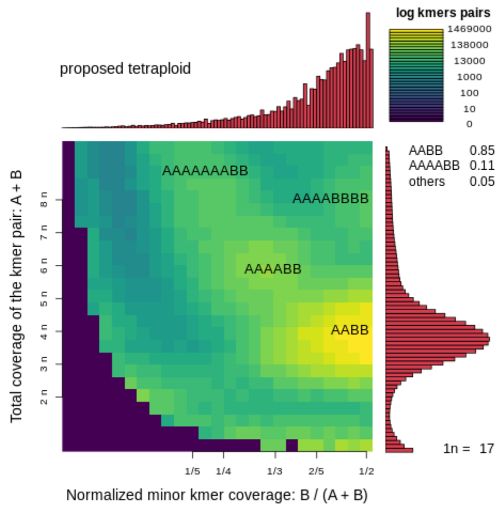

TETMER

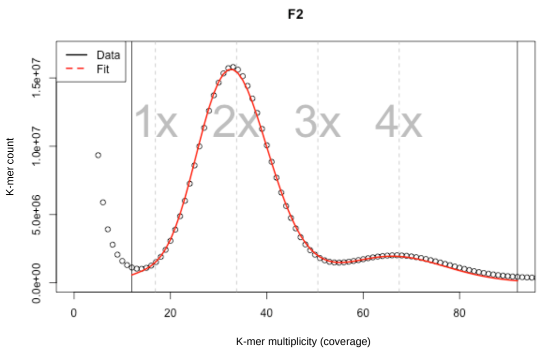


```
ALLOTETRAPLOID MODEL, AUTO FITTED
     haploid k-mer cov: 16.9
       per k-mer theta: 0.0133
                     T: 100
hapl non-rep GS (Mbp): 199.4
     bias (peak width): 0.7
      per k-mer diverg: 1.334

STARTING RANGES (MIN MAX)
     haploid k-mer cov: 11 21
log10 per k-mer theta: -3.7 0.2
                     T: 0.001 100
hapl non-rep GS (Mbp): 130 331
     bias (peak width): 0.1 1.36
              x range: 12 92
```

Euphrasia foulaensis (F3, tetraploid)

GenomeScpe2.0

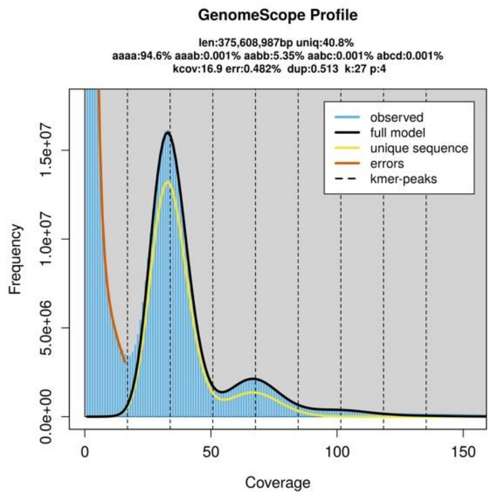


```
GenomeScope version 2.0
input file = /mnt/1TBSSD/Eukmers/numsE038
output directory = E038
p = 4
k = 27
initial kmercov estimate = 16

property                      min               max
Homozygous (aaaa)             91.8757%          94.8902%
Heterozygous (not aaaa)       5.10979%          8.12434%
aaab                          0%                1.0092%
aabb                          5.10979%          5.59446%
aabc                          0%                0.577994%
abcd                          0%                0.942694%
Genome Haploid Length         371,448,295 bp    375,608,987 bp
Genome Repeat Length          220,027,222 bp    222,491,806 bp
Genome Unique Length          151,421,073 bp    153,117,181 bp
Model Fit                     56.4736%          93.702%
Read Error Rate               0.482457%         0.482457%
```

Smudgeplot

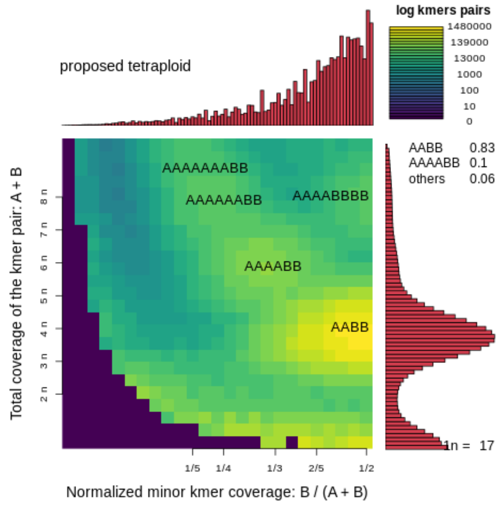

Tetmer

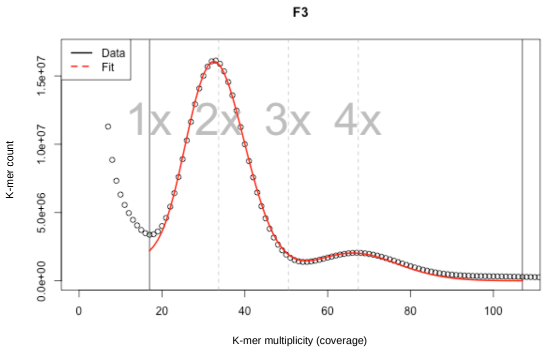


```
ALLOTETRAPLOID MODEL, AUTO FITTED
     haploid k-mer cov: 16.8
       per k-mer theta: 0.0262
                     T: 50
hapl non-rep GS (Mbp): 205.1
     bias (peak width): 0.6
      per k-mer diverg: 1.312

STARTING RANGES (MIN MAX)
     haploid k-mer cov: 10 34
log10 per k-mer theta: -2 0.6
                     T: 0.001 100
hapl non-rep GS (Mbp): 20 2000
     bias (peak width): 0.1 3
               x range: 17 107
```

Euphrasia foulaensis (F4, tetraploid)

GenomeScpe2.0

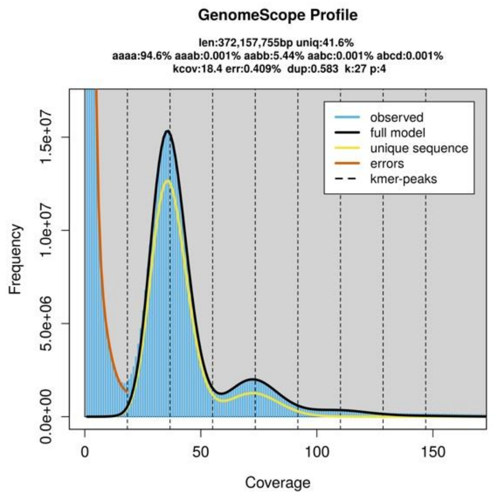


```
GenomeScope version 2.0
input file = /mnt/1TBSSD/Eukmers/numsE037
output directory = E037
p = 4
k = 27
initial kmercov estimate = 18

property                      min               max
Homozygous (aaaa)             92.3052%          94.815%
Heterozygous (not aaaa)       5.18496%          7.69476%
aaab                          0%                0.859625%
aabb                          5.18496%          5.70374%
aabc                          0%                0.416018%
abcd                          0%                0.715371%
Genome Haploid Length         369,079,264 bp    372,157,755 bp
Genome Repeat Length          215,414,425 bp    217,211,197 bp
Genome Unique Length          153,664,839 bp    154,946,558 bp
Model Fit                     57.621%           94.1044%
Read Error Rate               0.408706%         0.408706%
```

Smudgeplot

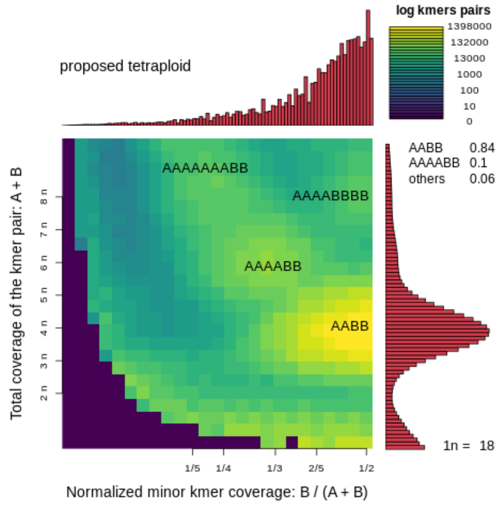

Tetmer

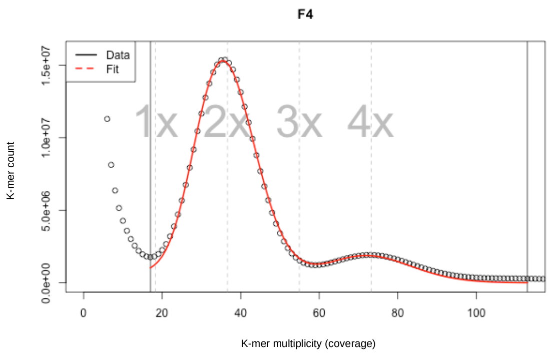


```
ALLOTETRAPLOID MODEL, AUTO FITTED
     haploid k-mer cov: 18.3
       per k-mer theta: 0.0134
                     T: 100
hapl non-rep GS (Mbp): 203.8
     bias (peak width): 0.7
      per k-mer diverg: 1.343

STARTING RANGES (MIN MAX)
     haploid k-mer cov: 17 31
log10 per k-mer theta: -2.65 0.6
                     T: 0.001 100
hapl non-rep GS (Mbp): 20 2000
     bias (peak width): 0.1 3
              x range: 17 113
```

EUPHRASIA MICRANTHA (M0, TETRAPLOID)

GENOMESCPE2.0

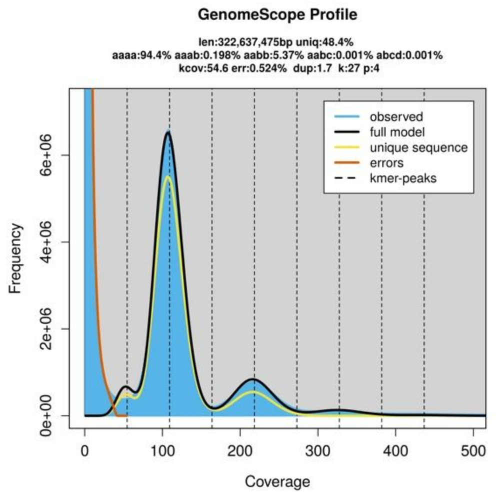


```
GenomeScope version 2.0
input file = /mnt/1TBSSD/Eukmers/numsE033
output directory = E033
p = 4
k = 27
initial kmercov estimate = 54

property                    min                  max
Homozygous (aaaa)           92.7157%             94.7738%
Heterozygous (not aaaa)     5.22624%             7.28431%
aaab                        0%                   0.851321%
aabb                        5.22624%             5.52321%
aabc                        0%                   0.406022%
abcd                        0%                   0.503752%
Genome Haploid Length       322,049,625 bp       322,637,475 bp
Genome Repeat Length        166,242,735 bp       166,546,184 bp
Genome Unique Length        155,806,890 bp       156,091,291 bp
Model Fit                   63.1628%             91.6415%
Read Error Rate             0.524025%            0.524025%
```

SMUDGEPLOT

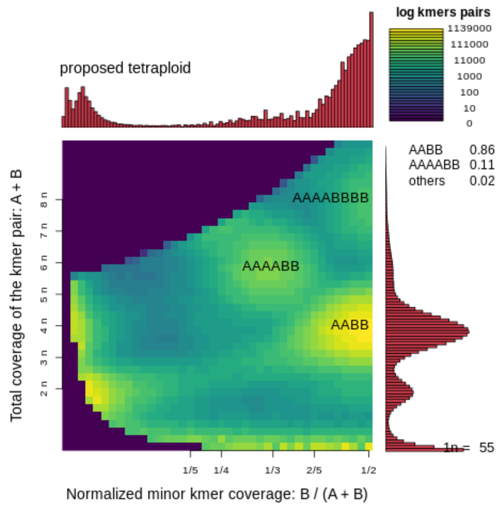

TETMER

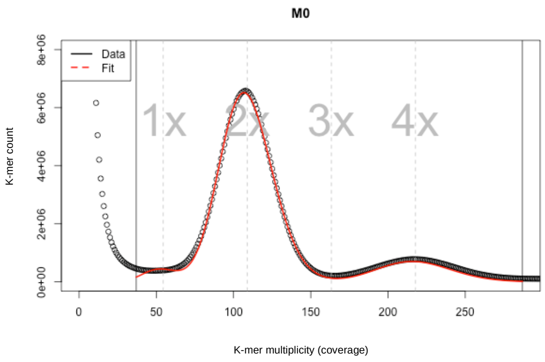


```
ALLOTETRAPLOID MODEL, AUTO FITTED
    haploid k-mer cov: 54.4
      per k-mer theta: 0.0205
                    T: 70.13
hapl non-rep GS (Mbp): 185.1
    bias (peak width): 1.7
       per k-mer diverg: 1.437

STARTING RANGES (MIN MAX)
    haploid k-mer cov: 34 63
log10 per k-mer theta: -3.85 -1.55
                    T: 0.001 100
hapl non-rep GS (Mbp): 117 345
```

```
      bias (peak width): 0.1 3
               x range: 37 287
```

EUPHRASIA MICRANTHA (M1, TETRAPLOID)

GENOMESCPE2.0

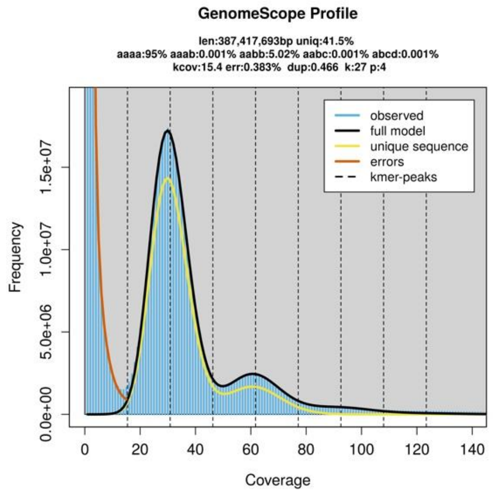


```
GenomeScope version 2.0
input file = /mnt/1TBSSD/Eukmers/numsE023
output directory = E023
p = 4
k = 27
initial kmercov estimate = 15

property                      min               max
Homozygous (aaaa)             92.6876%          95.1105%
Heterozygous (not aaaa)       4.88955%          7.31239%
aaab                          0%                0.732638%
aabb                          4.88955%          5.14633%
aabc                          0%                0.651672%
abcd                          0%                0.781753%
Genome Haploid Length         382,918,749 bp    387,417,693 bp
Genome Repeat Length          223,967,285 bp    226,598,695 bp
Genome Unique Length          158,951,464 bp    160,818,998 bp
Model Fit                     56.8672%          95.5142%
Read Error Rate               0.383123%         0.383123%
```

SMUDGEPLOT

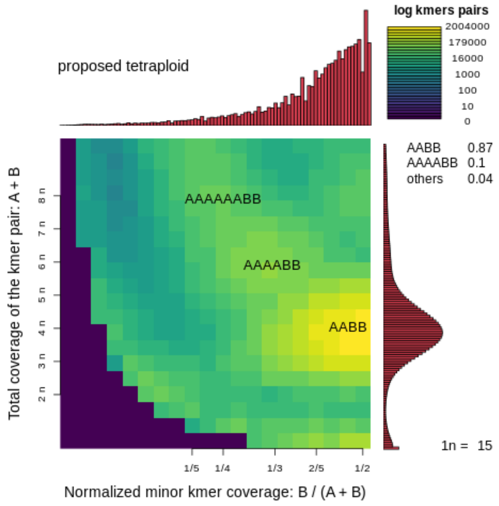

TETMER

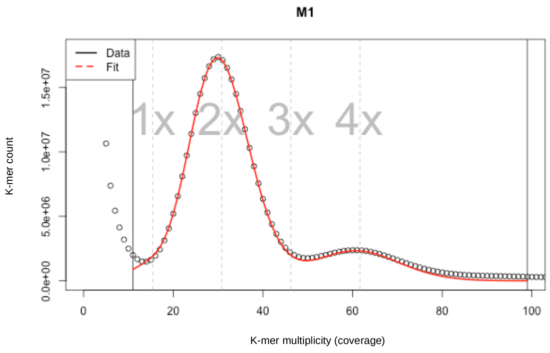


```
ALLOTETRAPLOID MODEL, AUTO FITTED
      haploid k-mer cov: 15.4
         per k-mer theta: 0.0196
                      T: 64.79
hapl non-rep GS (Mbp): 201.7
      bias (peak width): 0.4
        per k-mer diverg: 1.272

STARTING RANGES (MIN MAX)
      haploid k-mer cov: 10 18
log10 per k-mer theta: -4 0.6
                      T: 0.001 80
hapl non-rep GS (Mbp): 20 400
```

bias (peak width): 0.1 1.66
x range: 11 99

EUPHRASIA MICRANTHA (M2, TETRAPLOID)

GENOMESCPE2.0

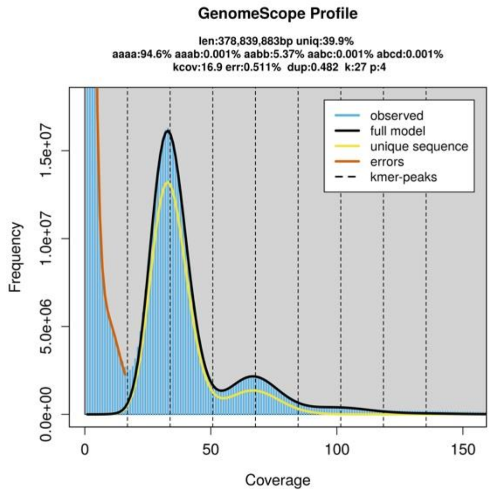


```
GenomeScope version 2.0
input file = /mnt/1TBSSD/Eukmers/numsE022
output directory = E022
p = 4
k = 27
initial kmercov estimate = 17

property                      min                 max
Homozygous (aaaa)             91.796%             94.9132%
Heterozygous (not aaaa)       5.08677%            8.20403%
aaab                          0%                  1.05657%
aabb                          5.08677%            5.64331%
aabc                          0%                  0.583533%
abcd                          0%                  0.920616%
Genome Haploid Length         374,837,102 bp      378,839,883 bp
Genome Repeat Length          225,133,438 bp      227,537,576 bp
Genome Unique Length          149,703,664 bp      151,302,307 bp
Model Fit                     56.0751%            94.4486%
Read Error Rate               0.510853%           0.510853%
```

SMUDGEPLOT

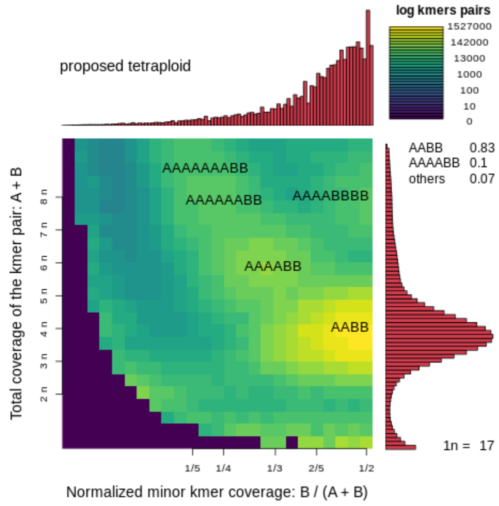

TETMER

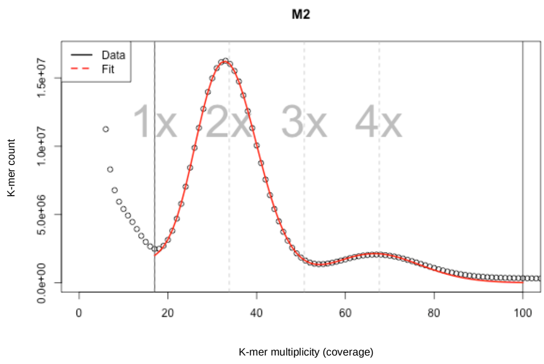


```
ALLOTETRAPLOID MODEL, AUTO FITTED
     haploid k-mer cov: 16.9
       per k-mer theta: 0.0256
                     T: 50.03
hapl non-rep GS (Mbp): 202.7
     bias (peak width): 0.5
      per k-mer diverg: 1.283

STARTING RANGES (MIN MAX)
     haploid k-mer cov: 15 24
log10 per k-mer theta: -3.75 0.6
```

```
                  T: 0.001 100
hapl non-rep GS (Mbp): 20 2000
    bias (peak width): 0.1 3
             x range: 17 100
```

Euphrasia micrantha (M3, tetraploid)

GenomeScpe2.0

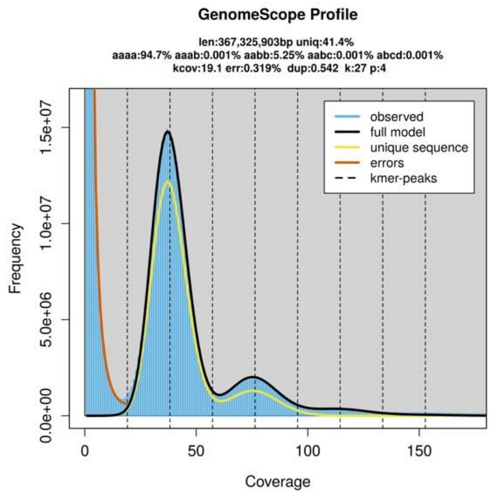


```
GenomeScope version 2.0
input file = /mnt/1TBSSD/Eukmers/numsE035
output directory = E035
p = 4
k = 27
initial kmercov estimate = 19

property                      min                 max
Homozygous (aaaa)             92.8567%            94.9359%
Heterozygous (not aaaa)       5.06408%            7.14332%
aaab                          0%                  0.7246%
aabb                          5.06408%            5.4417%
aabc                          0%                  0.397073%
abcd                          0%                  0.579947%
Genome Haploid Length         365,165,005 bp      367,325,903 bp
Genome Repeat Length          213,881,262 bp      215,146,924 bp
Genome Unique Length          151,283,743 bp      152,178,979 bp
Model Fit                     57.1831%            93.7891%
Read Error Rate               0.318804%           0.318804%
```

Smudgeplot

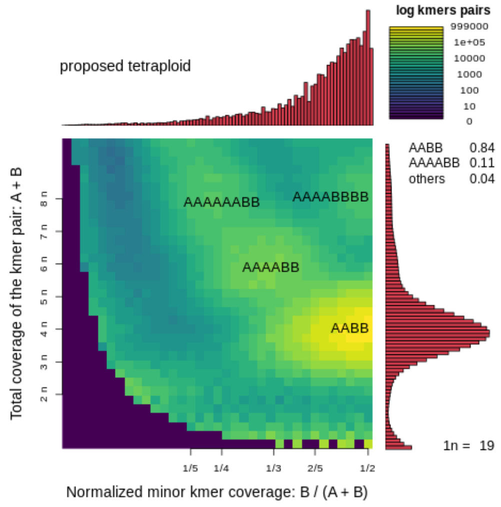

Tetmer

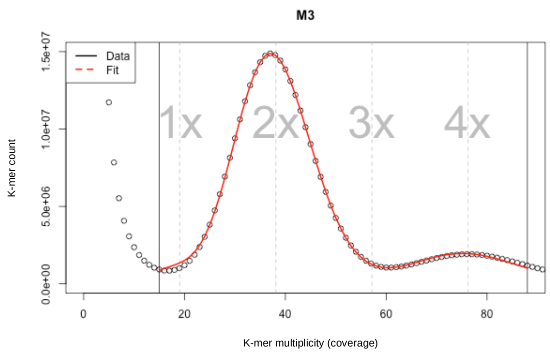


```
ALLOTETRAPLOID MODEL, AUTO FITTED
    haploid k-mer cov: 19.1
      per k-mer theta: 0.0198
                    T: 65.13
hapl non-rep GS (Mbp): 198.8
    bias (peak width): 0.5
     per k-mer diverg: 1.288

STARTING RANGES (MIN MAX)
    haploid k-mer cov: 15 21
log10 per k-mer theta: -3.85 0.6
                    T: 30 100
hapl non-rep GS (Mbp): 148 279
    bias (peak width): 0.1 3
              x range: 15 88
```

EUPHRASIA SP. (X1, TETRAPLOID)

GENOMESCPE2.0

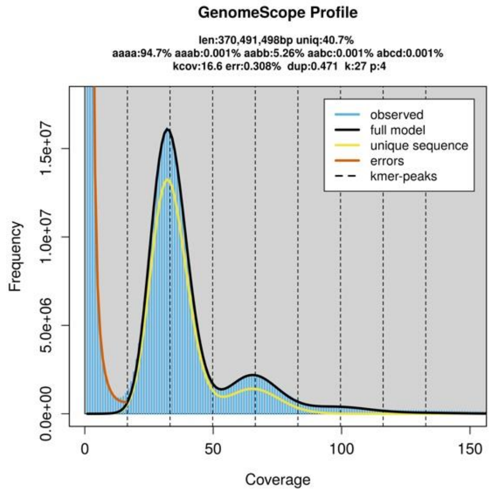


```
GenomeScope version 2.0
input file = /mnt/1TBSSD/Eukmers/numsE034
output directory = E034
p = 4
k = 27
initial kmercov estimate = 16

property                     min                max
Homozygous (aaaa)            92.6014%           94.9449%
Heterozygous (not aaaa)      5.05514%           7.39865%
aaab                         0%                 0.79699%
aabb                         5.05514%           5.46933%
aabc                         0%                 0.445348%
abcd                         0%                 0.686974%
Genome Haploid Length        367,332,163 bp     370,491,498 bp
Genome Repeat Length         217,729,861 bp     219,602,503 bp
Genome Unique Length         149,602,302 bp     150,888,995 bp
Model Fit                    56.602%            94.8849%
Read Error Rate              0.307797%          0.307797%
```

SMUDGEPLOT

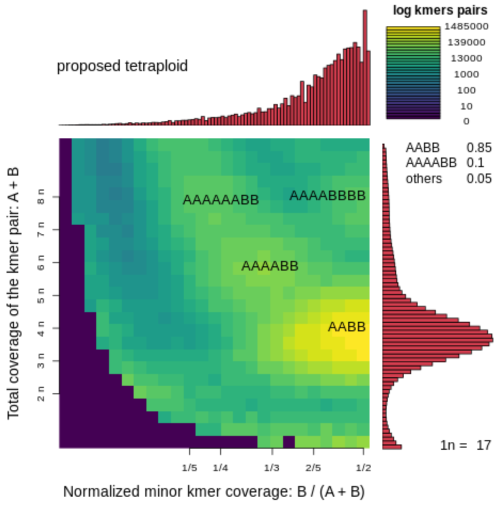

TETMER

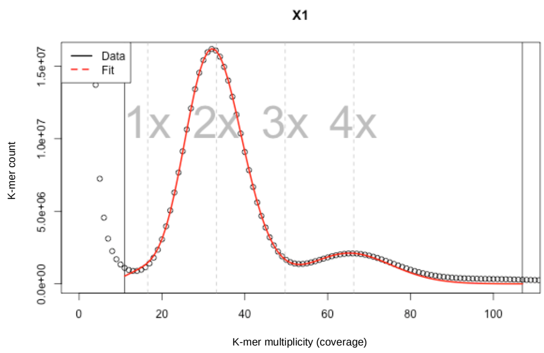


```
ALLOTETRAPLOID MODEL, AUTO FITTED
     haploid k-mer cov: 16.6
       per k-mer theta: 0.0158
                     T: 81.86
hapl non-rep GS (Mbp): 196
     bias (peak width): 0.5
      per k-mer diverg: 1.297

STARTING RANGES (MIN MAX)
     haploid k-mer cov: 15 19
log10 per k-mer theta: -4 -1.8
                     T: 0.001 100
hapl non-rep GS (Mbp): 20 235
     bias (peak width): 0.1 0.46
              x range: 11 107
```

Euphrasia sp. (X2, tetraploid)

GenomeScpe2.0

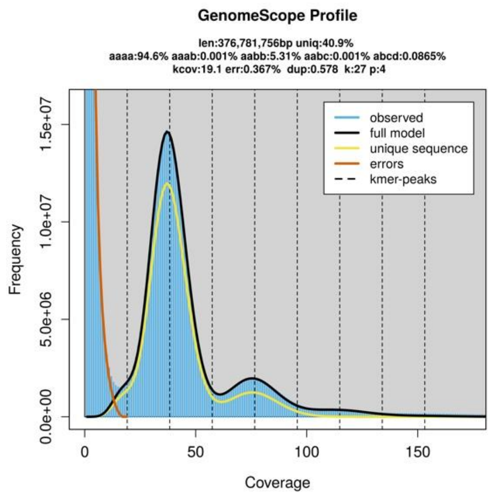


```
GenomeScope version 2.0
input file = /mnt/1TBSSD/Eukmers/numsE039
output directory = E039
p = 4
k = 27
initial kmercov estimate = 19

property                    min              max
Homozygous (aaaa)           92.6551%         94.9255%
Heterozygous (not aaaa)     5.0745%          7.3449%
aaab                        0%               0.745196%
aabb                        5.0745%          5.54892%
aabc                        0%               0.405733%
abcd                        0%               0.645054%
Genome Haploid Length       374,819,298 bp   376,781,756 bp
Genome Repeat Length        221,669,233 bp   222,829,836 bp
Genome Unique Length        153,150,065 bp   153,951,920 bp
Model Fit                   56.9085%         94.3288%
Read Error Rate             0.367329%        0.367329%
```

Smudgeplot

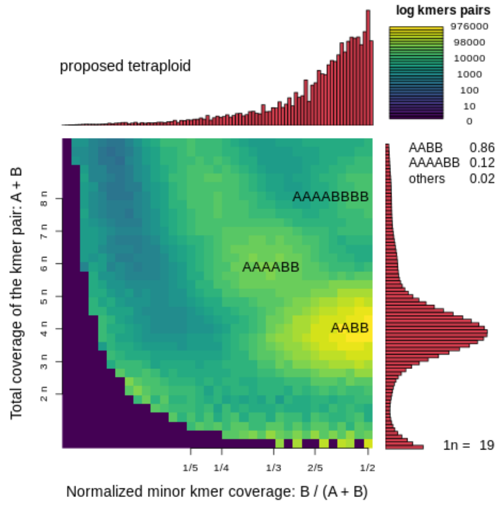

Tetmer

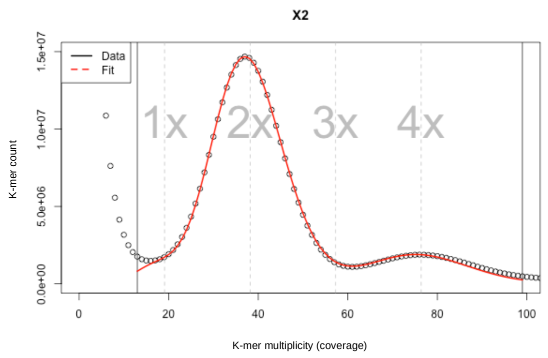


```
ALLOTETRAPLOID MODEL, AUTO FITTED
    haploid k-mer cov: 19.1
      per k-mer theta: 0.0259
                    T: 50.03
hapl non-rep GS (Mbp): 202.6
    bias (peak width): 0.6
     per k-mer diverg: 1.298

STARTING RANGES (MIN MAX)
    haploid k-mer cov: 13 20
log10 per k-mer theta: -2 0.6
                    T: 0.001 100
hapl non-rep GS (Mbp): 20 2000
    bias (peak width): 0.1 3
              x range: 13 99
```
